# Brønsted acid-mediated cyclization–dehydrosulfonylation/reduction sequences: An easy access to pyrazinoisoquinolines and pyridopyrazines

**DOI:** 10.3762/bjoc.13.46

**Published:** 2017-03-07

**Authors:** Ramana Sreenivasa Rao, Chinnasamy Ramaraj Ramanathan

**Affiliations:** 1 Department of Chemistry, Pondicherry University, Puducherry – 605 014, India

**Keywords:** Brønsted acid, piperazine-2,6-diones, praziquantel, pyrazinoisoquinoline, pyridopyrazine

## Abstract

An efficient and alternative synthetic approach has been developed to prepare various *N*-(arylethyl)piperazine-2,6-diones from 4-benzenesulfonyliminodiacetic acid and primary amines using carbonyldiimidazole in the presence of a catalytic amount of DMAP at ambient temperature. Piperazine-2,6-diones are successfully transformed to pharmaceutically useful pyridopyrazines or pyrazinoisoquinolines and ene-diamides via an imide carbonyl group activation strategy using a Brønsted acid. Subsequent dehydrosulfonylation reactions of the ene-diamides, in a one pot manner, smoothly transformed them to substituted pyrazinones. A concise synthesis of praziquantel (**1**) has also been achieved through this method.

## Introduction

**Figure 1:**
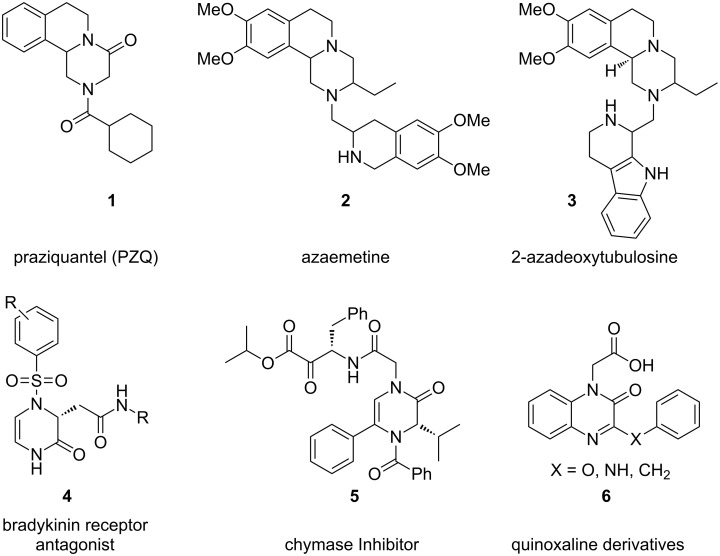
Selected active pyrazinoisoquinolines, 2-oxopiperazines and aldose reductase inhibitors (ALR2).

Piperazine is an important structural core found in many biologically active natural products [1]. Piperazines are also useful intermediates in the synthesis of a variety of drug molecules having important biological activities such as anticancer [2–3], antifungal [4], amoebiasis, trypanosomiasis, bilharziasis [5–6], and schistosomiasis [7–8]. Some are cell adhesion inhibitors [9] brandykinin receptor antagonists [10–11] and chymase inhibitors [12]. Quinoxaline derivatives are known to act as aldose reductase (ALR_2_) inhibitors and are active towards chronic diabetic complications including neuropathy, nephropathy, cataracts and retinopathy [13–14]. On the other hand, piperazinone condensed tetrahydroisoquinolines (THIQs) are widespread in nature with interesting biological activities [15]. They are also found to be building blocks for the synthesis of many alkaloids (Figure 1) [16].

To synthesize pyrazinoisoquinoline and its derivatives, various approaches such as the Ugi multicomponent reaction [17] amidoalkylation [18–19], *N*-acyliminium ion cyclization [20] and a radical cyclization [21] have been reported. To this end, recently, we have reported the activation of an imide carbonyl group with TfOH, for the synthesis of tetrahydroisoquinoline (THIQ) and tetrahydro-β-carboline (THBC) skeletons and related alkaloids [22–26].

The present study, describes the synthesis of 4-benzenesulfonylpiperazine-2,6-dione derivatives by using the combination of carbonyldiimidazole/4-dimethylaminopyridine (CDI/DMAP). Even though there are an adequate number of reports available in the literature for the synthesis of piperazine-2,6-dione derivatives [27]. To the best of our knowledge, this is the first report describing the synthesis of 4-benzenesulfonylpiperazine-2,6-diones at ambient temperature. The advantage of this methodology is that the more reactive and acid labile groups can be installed to the piperazine-2,6-diones at N-4 position, which would then be utilized for synthetically useful transformations to yield the corresponding products.

Further, these 4-benzenesulfonylpiperazine-2,6-diones were subjected to an imide carbonyl group activation strategy, to develop a practical approach to synthesize pyrazinoisoquinoline and pyridopyrazines via Brønsted acid assisted *6-exo-trig* cyclization of arylethylpiperazine-2,6-diones.

## Results and Discussion

**Scheme 1:**
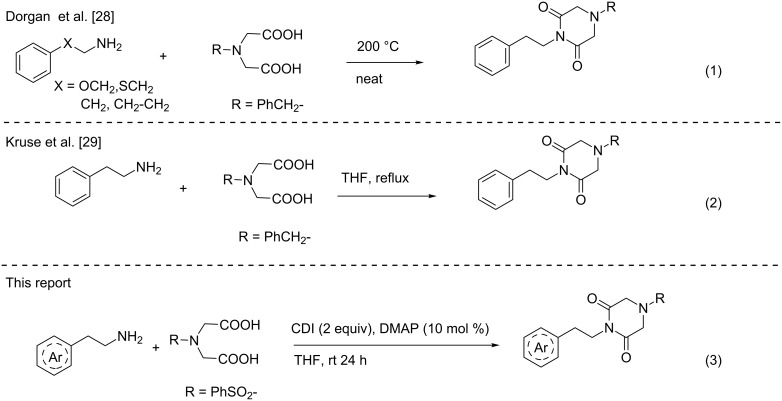
Comparison of previous reports with present work for piperazine-2,6-dione synthesis.

Piperazine-2,6-diones are usually synthesized through an Ugi [30] multicomponent approach. The condensation of primary amines with benzyliminodiacetic acid at high temperatures or CDI/THF at reflux leads also to the formation of piperazine-2,6-diones (Scheme 1). Using CDI/THF under reflux conditions, the piperazine-2,6-dione **7a** from 4-benzenesulfonyliminodiacetic acid was obtained only in 50% yield along with the formation of benzenesulfonic acid (Table 1, entry 1). The formation of benzenesulfonic acid may be due to the labile nature of the benzenesulfonyl protecting group. Therefore, the reactions were performed at temperatures below 70 °C and a higher yield of piperazine-2,6-dione **7a** was observed at 60 °C (Table 1, entry 2). Further lowering of the reaction temperature did not improve the yield of **7a** (Table 1, entry 3). Hence, there is a need to develop a method for the preparation of piperazine-2,6-dione derivatives from *N*-benzenesulfonyliminodiacetic acid at room temperature. After careful reviewing of reagents for amide bond formation, the reagent CDI proved to be a very successful reagent for the preparation of imides, amides, esters and thioesters. Therefore, the reaction was carried out with *N*-benzenesulfonyliminodiacetic acid, a primary amine and CDI (2 equiv) in THF, the corresponding piperazine-2,6-dione **7a** was obtained in 21% yield at room temperature (Table 1, entry 4).

**Table 1:** Standardization for the preparation of piperazine-2,6-dione.^a^

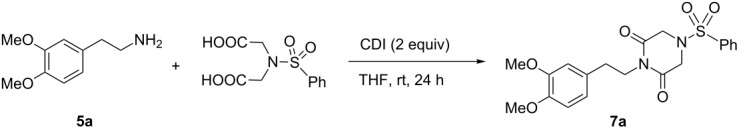					

Entry	Reagents	Equivalent	Temp (°C )	Time (h)	Yield (%)^b^

1	CDI	2	70	24	50
2	CDI	2	60	24	61
3	CDI	2	40	24	42
4	CDI	2	rt	48	21
5	CDI/DMAP	2/1	rt	24	35
6	CDI/DMAP	2/0.5	rt	24	50
7	CDI/DMAP	2/0.25	rt	24	57
8	CDI/DMAP	2/0.1	rt	24	72

^a^Reaction conditions: amine **5a** (1 mmol), benzenesulfonyl substituted iminodiacetic acid (1 mmol), THF (15 mL), rt. ^b^Yields of isolated products.

**Scheme 2:**
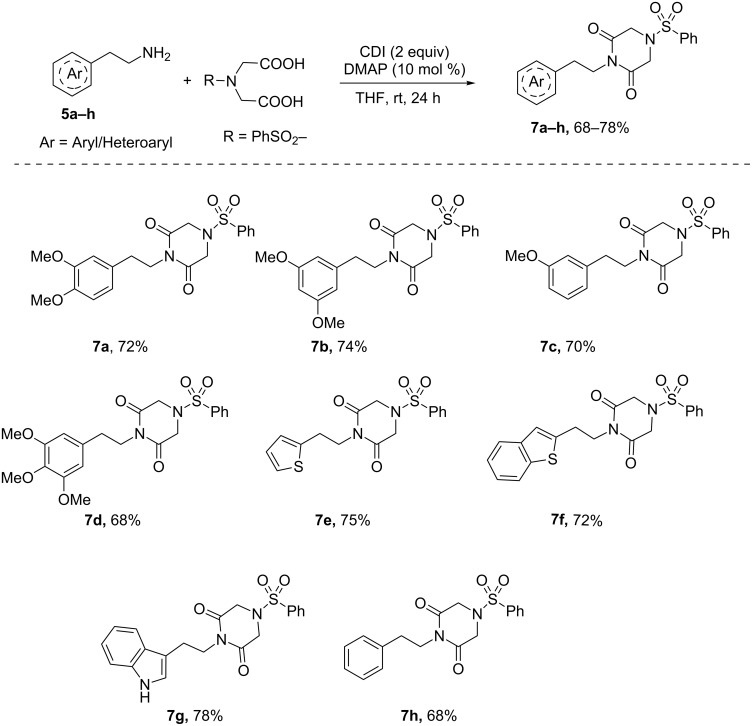
Coupling of *N*-benzenesulfonyliminodiacetic acid with primary amines using CDI/DMAP.

To facilitate the peptide bond formation DMAP has been used in conjuncture with coupling reagents such as DCC. We presumed that the use of DMAP along with CDI may facilitate the imide bond formation at room temperature. Hence, the reaction of **5a** with *N*-benzenesulfonyliminodiacetic acid was carried out using CDI in the presence of DMAP (Table 1, entries 5–8). Piperazine-2,6-dione **7a** was obtained in 72% yield at room temperature in the presence of 10 mol % of DMAP. Similarly, this strategy has been extended to couple various arylethylamines with *N*-benzenesulfonyliminodiacetic acid at room temperature to furnish expected imides **7a**–**h** in good yields (Scheme 2).

The successful development of this methodology for the synthesis of 1-arylethylpiperazine-2,6-diones intrigued us to examine them in *6-exo-trig* cyclization using TfOH followed by reduction with NaBH_4_/MeOH to accomplish the synthesis of tetrahydropyrazinoisoquinoline. Surprisingly, the reaction of **7a** furnished a mixture of ene-diamide **9a** and substituted pyrazinone **10a** in 90:10 ratio. Generally, the syntheses of these types of units are very limited in the literature [31]. Under controlled experimental conditions, in the absence of NaBH_4_/MeOH, the ene-diamide **9a** was successfully generated in 90% yield using 4 equivalents of TfOH in 30 minutes from **7a**.

**Scheme 3:**
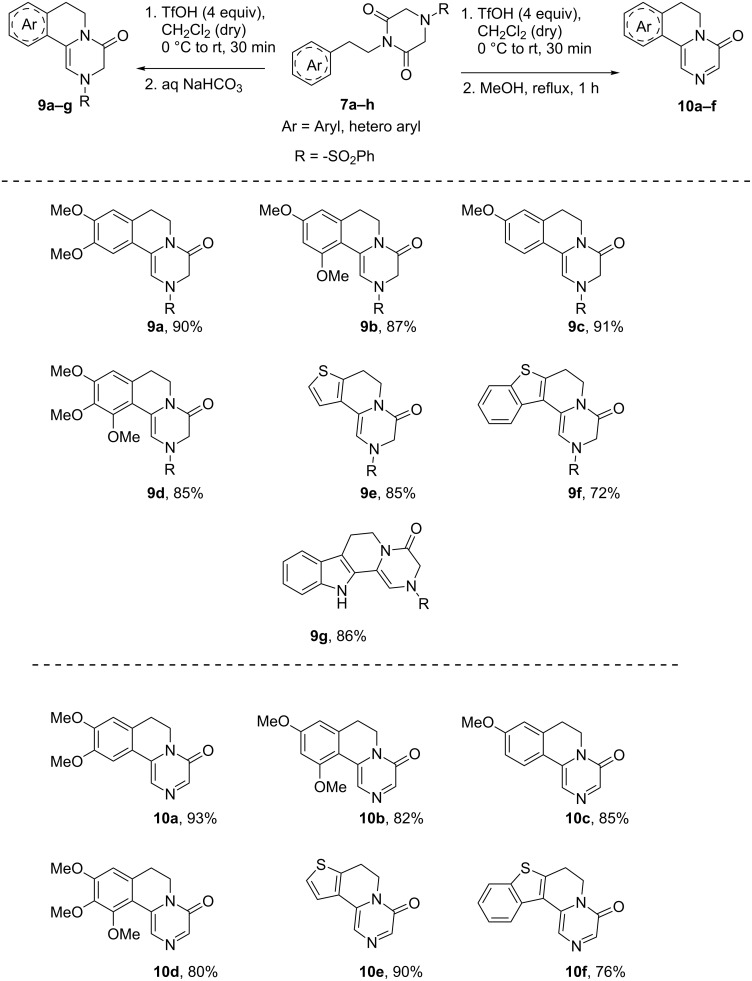
Formation of ene-diamides **9a**–**g** and pyrazinones **10a**–**f**.

While increasing the reaction time from 30 minutes to 2 h, the formation of biologically active pyrazinone **10a** has been realized as a major product along with ene-diamide **9a**. A literature survey revealed that sulfonamides are known to undergo hydrolysis in the presence of a Brønsted acid [32]. Sulfonamides also participate in amide hydrolysis with external nucleophiles such as phosphide anions [33] or phenyldimethylsilyllithium [34]. The combinations of thiophenol/K_2_CO_3_ [35] or NaOH/MeOH [36] are also known to hydrolyse sulfonamides. These methods lead to the formation of the corresponding free amines. Such desulfonylation of sulfonamides has been less utilized to make an unsaturated bond, for example, imine. Hence, attention has been paid to find suitable conditions for the formation of pyrazinones directly from piperazine-2,6-diones via cyclization followed by dehydrosulfonylation (Scheme 3).

**Scheme 4:**
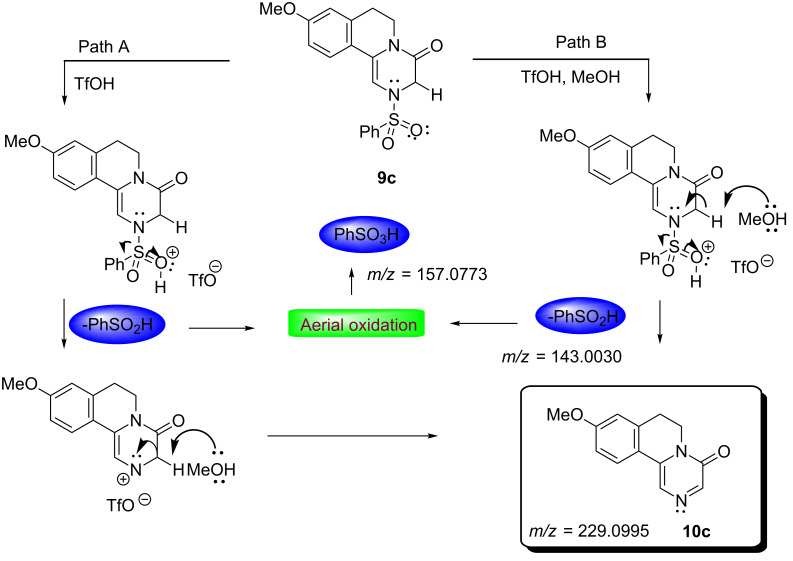
Mechanism for the formation of substituted pyrazinones.

**Figure 2:**
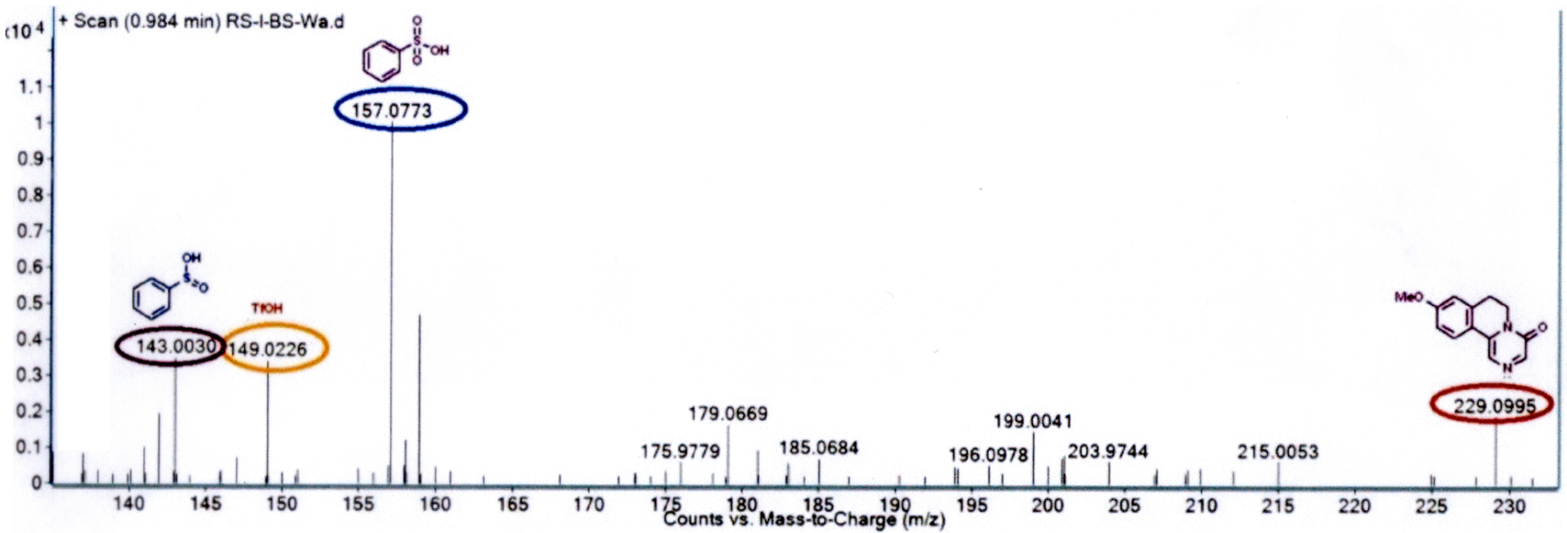
HRMS spectra of aliquot generated from cyclization reaction of **7c**.

Following extensive optimization, it was realized that after cyclization, addition of MeOH followed by reflux proved to be suitable conditions to generate the substituted pyrazinones. The formation of substituted pyrazinones through dehydrosulfonylation (1,2-elimination) may be facilitated by 3-factors, such as, (i) extended conjugation in pyrazinones, (ii) good leaving capacity of the benzenesulfonyl group and (iii) the presence of an acidic proton which is α to the amide carbonyl group. Hence, we believe that the selective elimination of the sulfonyl group in refluxing methanol would serve as a simple and novel alternate methodology to the reported oxidative strategy [37] for the syntheses of substituted pyrazinones. To demonstrate the generality of this procedure various methoxy/methyl substituted phenethyl and heterocyclic ethylpiperazine-2,6-diones **7a**–**g** have been successfully converted to the substituted pyrazinones **10a**–**f** in excellent yields as shown in Scheme 3. To elucidate the mechanism involved in the formation of substituted pyrazinones, the aliquot obtained from the reaction of **7c** with TfOH followed by reflux in MeOH, was analyzed through ESI–HRMS technique. The appearance of peaks at *m*/*z* = 143.0030 and *m*/*z* = 159.0773 indicates the formation of benzenesulfinic acid and benzenesulfonic acid, respectively. The formation of benzenesulfinic acid could be explained through a desulfonylization reaction via a 1,2-elimination process (Scheme 4 and Figure 2).

**Figure 3:**
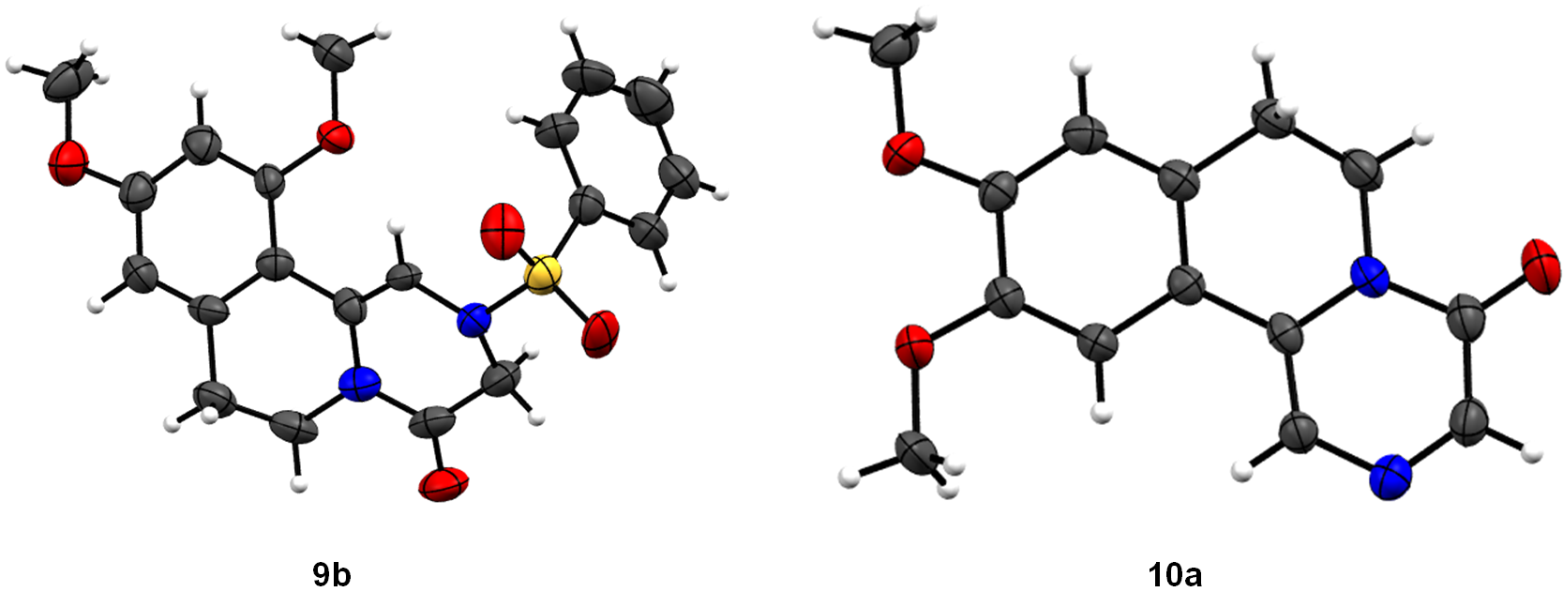
ORTEP diagrams of compound **9b** and **10a**.

The structural evidence for cyclized compounds **9b** and **10a** was supported by single-crystal X-ray diffraction analysis (Figure 3) in addition to IR, NMR and HRMS data.

**Scheme 5:**
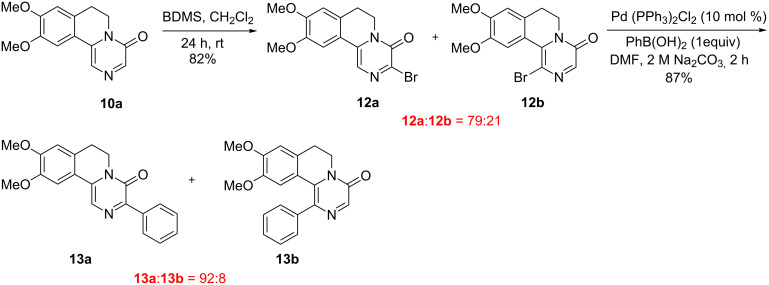
Synthesis of 3-phenylpyrazinone.

To show the synthetic utility of substituted pyrazinones, we introduce a phenyl group at the C-3 position in pyrazinone derivative **10a**. To begin with, the phenyl group was proposed to introduce at the 3-position in substituted pyrazinone via regioselective bromination followed by Suzuki coupling with phenylboronic acid. Accordingly, the bromination of **10a** was successfully carried out to furnish regioisomers **12a** and **12b** in 82% yield upon treatment with bromodimethylsulfonium bromide (BDMS) [38] in dichloromethane at 0 °C to room temperature. The conventional Suzuki coupling of the regioisomers **12a** and **12b** with phenylboronic acid furnished the corresponding arylated products **13a** and **13b** in excellent yield (Scheme 5).

**Scheme 6:**
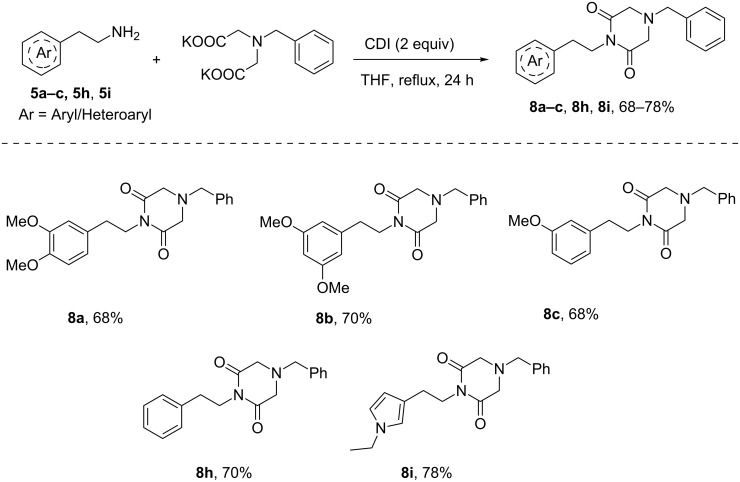
Synthesis of 4-*N*-benzyl-1-*N*-(aryl/heteroarylethyl)piperazine-2,6-dione.

To our dismay, the imide **7h** did not participate in the cyclization reaction in the presence of TfOH at room temperature to generate a potential precursor for the synthesis of praziquantel. To realize the cyclization, the imide **7h** was treated with TfOH under neat conditions at 70 °C, quite unfortunately to witness the decomposition of imide **7h**. This may be due to the labile nature of the benzenesulfonyl group in **7h**. To avoid this decomposition problem, the amino group in iminodiacetic acid was protected as a *N*-benzyl group instead of a *N*-benzenesulfonyl group. Accordingly, the potassium salt of *N*-benzyliminodiacetic acid was synthesized following the reported procedure [39]. The *N*-protected *N*-phenethylpiperazine-2,6-dione **8h** was formed, while treating the potassium salt of *N*-benzyliminodiacetic acid with phenethylamine in presence of CDI in THF under reflux conditions. Similarly, this strategy has been extended to couple arylethylamines with the potassium salt of *N*-benzyliminodiacetic acid in THF at reflux to furnish the expected imides **8a**–**c**, **8h**, and **8i** in good yields (Scheme 6).

**Scheme 7:**
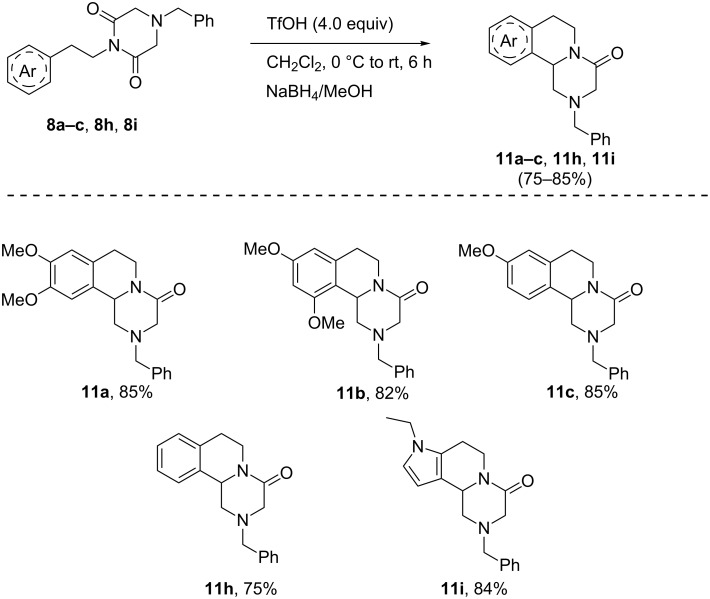
Cyclization of pyrazinoisoquinolines.

The imide **8h** was subjected to the cyclization reaction conditions using 6 equivalents of TfOH under neat conditions at 70 °C followed by reduction using NaBH_4_/MeOH at room temperature, which successfully furnished *N*-benzylpraziquantel **11h** in 75% yield. The electron rich phenethyl group containing imides **8a**–**c**, electron rich heteroaryl ethyl imide **8i**, smoothly delivered the cyclized products **11a**–**c** and **11i** in excellent yields through the cyclization followed by the reduction sequence at room temperature (Scheme 7).

**Scheme 8:**
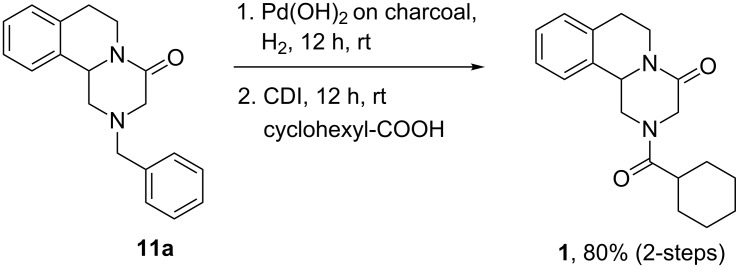
Synthesis of the drug praziquantel **1**.

The synthesis of praziquantel from intermediate **11h** was accomplished through *N*-debenzylation at 80 °C under 1 atmosphere of H_2_ in the presence of Pd/C [40]. The present synthetic protocol involves the debenzylation reaction under milder conditions using Pd(OH)_2_ on charcoal/H_2_ at room temperature followed by coupling the secondary amine with cyclohexanecarboxylic acid using CDI. Praziquantel (**1**) was obtained in 80% overall yield from **11h** (Scheme 8).

## Conclusion

In conclusion, an efficient and alternative synthetic approach has been developed to prepare various *N*-(arylethyl)piperazine-2,6-diones. Brønsted acid assisted *6-exo-trig* cyclization of piperazine-2,6-dione derivatives of aryl/heteroarylethylamines through carbonyl group activation to assemble the pyridoisoquinoline and pyrazinoisoquinoline derivatives have been demonstrated. The ene-diamides furnished the substituted pyrazinones through an acid–mediated dehydrosulfonylation in methanol. This strategy can be adopted to develop value added pyrazinone-based potential precursors useful to synthesize various drug targets. Further, the synthetic utility of pyrazinoisoquinoline was exemplified by the successful synthesis of fused tetrahydroisoquinoline drug molecule, praziquantel. Extending this strategy towards the stereoselective reduction of pyrazinones/ene-diamides is under investigation in our laboratory.

## Experimental

### General procedure for the synthesis of 4-benzenesulfonylpiperazinone-2,6-diones **7a–h**

An oven-dried two-neck round-bottom flask that had a septum in the side arm was cooled to room temperature under a steady stream of nitrogen. The flask was charged with a stir bar, benzenesulfonyliminodiacetic acid (1 mmol), carbonyldiimidazole (2 mmol) and 10 mol % DMAP and dry THF (25 mL). Stirring was continued at room temperature to provide a clear solution. To this mixture was added aryl and heteroarylethylamine (**5a**–**h**) (1 mmol) and the resulting solution was stirred at room temperature for 24 h (monitored by TLC). The solution was concentrated to dryness using a rotary evaporator under reduced pressure. The crude reaction product was purified by chromatography on a short silica gel column using ethyl acetate/hexane, (30:70) as eluent to afford **7a**–**h** in pure form.

#### 1-(3,4-Dimethoxyphenethyl)-4-(phenylsulfonyl)piperazine-2,6-dione (**7a**)

300 mg, (72% yield) colorless solid; mp 138–139 °C; IR (KBr, cm^−1^): 3002, 2937, 2835, 1686, 1515, 1447, 1391, 1270, 1237, 1171, 1114, 1026, 960, 852, 811, 811, 690, 668, 572, 525; ^1^H NMR (400 MHz, CDCl_3_) δ 7.79–7.78 (m, 2H), 7.66–7.62 (m, 1H), 7.59–7.55 (m, 2H), 7.67 (d, *J* = 7.3 Hz, 1H), 6.69–6.66 (m, 2H), 4.11 (s, 4H), 3.86 (s, 3H), 3.84 (s, 3H), 3.6–3.65 (m, 2H), 2.43–2.47 (m, 2H); ^13^C NMR (100 MHz, CDCl_3_) δ 166.4, 149.0, 147.9, 135.6, 134.2, 130.0, 127.7, 120.9, 112.1, 111.4, 56.0, 48.9, 40.1, 33.4; HRMS–ESI (*m*/*z*): [M + H] calcd for C_20_H_22_N_2_O_6_S, 419.1277; found, 419.1268.

### General procedure for synthesis of 4-benzylpiperazinone-2,6-diones **8a–c**, **8h**, **8i**

An oven-dried two-neck round-bottom flask that had a septum in the side arm was cooled to room temperature under a steady stream of nitrogen. The flask was charged with a stir bar, the potassium salt of benzyliminodiacetic acid (1 mmol) and carbonyldiimidazole (2 mmol) were added to dry THF (25 mL). The mixture was heated at refluxed for 10 min to obtain a clear solution. To this was added the aryl and heteroarylethylamines (**5a**–**c**, **5h**, **5i**) (1 mmol) and the resulting solution was heated at reflux for 24 h, then allowed to cool to room temperature. The solution was concentrated to dryness using a rotary evaporator under reduced pressure. The crude product was purified on a short silica gel column chromatography using ethyl acetate/hexane (2:3) as eluent to afford **8a**–**c**, **8h**, and **8i** in pure form.

#### 4-Benzyl-1-(3,4-dimethoxyphenethyl)piperazine-2,6-dione (**8a**)

250 mg, (68% yield) yellow liquid; IR (KBr, cm^−1^): 2950, 2853, 1734, 1683, 1594, 1514, 1453, 1348, 1268, 1156, 1028, 806, 755, 704, 631; ^1^H NMR (400 MHz, CDCl_3_) δ 7.29–7.26 (m, 1H), 7.24–7.21 (m, 2H), 7.19–7.16 (m, 2H), 6.70 (s, 2H), 6.68 (s, 1H), 3.94–3.86 (m, 2H), 3.79 (s, 3H), 3.76 (s, 3H), 3.49 (s, 2H), 3.29 (s, 4H), 2.72-2.68 (m, 2H); ^13^C NMR (100 MHz, CDCl_3_) δ 169.8, 148.9, 147.7, 135.4, 130.8, 129.1, 128.7, 129.0, 121.0, 112.2, 111.3, 60.7, 56.3, 55.9, 40.4, 33.5; HRMS–ESI (*m*/*z*): [M + H] calcd for C_21_H_24_N_2_O_4_, 369.1814; found, 369.1808.

### General procedure for the cyclization of 4-benzenesulfonylpiperazinone-2,6-diones **7a–g**

Analogous as described in [26] an oven-dried two-neck round-bottom flask that had a septum in the side arm was cooled to room temperature under a steady stream of nitrogen. The flask was charged with a stir bar, imide **7a–g** (0.5 mmol), and dry dichloromethane (15 mL), and the resulting solution was cooled to 0 °C (by using ice). To this solution was added TfOH (0.2 mL, 2 mmol) with stirring. After 30 min, the reaction mixture was quenched with aqueous NaHCO_3_. The organic layer was separated, and the aqueous layer was extracted with dichloromethane (2 × 15 mL). The combined organic extracts were washed with brine solution, dried over anhydrous Na_2_SO_4_ and filtered. The solution was concentrated to dryness by using a rotary evaporator. The dried crude product was purified by chromatography on a short silica gel chromatography column using ethyl acetate/hexane (1:1) as eluent to afford the **9a–g** in pure form.

### 9,10-Dimethoxy-2-(phenylsulfonyl)-2,3,6,7-tetrahydro-4*H*-pyrazino[2,1-*a*]isoquinolin-4-one (**9a**)

360 mg, (90% yield) colorless liquid; IR (KBr, cm^−1^): 2927, 2854, 1679, 1513, 1406, 1389, 1355, 1272, 1209, 1163, 1030, 992, 726, 576; ^1^H NMR (400 MHz, CDCl_3_) δ 7.82–7.79 (m, 2H), 7.59–7.55 (m, 1H), 7.51–7.47 (m, 2H), 6.92 (s, 1H), 6.58 (d, *J* = 7.6 Hz, 2H), 4.18 (s, 2H), 3.91 (s, 3H), 3.86 (s, 3H), 3.48–3.45 (m, 2H), 2.58–2.55 (m, 2H); ^13^C NMR (100 MHz, CDCl_3_) δ 162.0, 149.9, 148.6, 137.0, 133.6, 129.3, 127.3, 127.0, 119.9, 110.9, 106.4, 104.0, 56.4, 56.0, 48.3, 38.1, 28.0; HRMS–ESI (*m*/*z*): [M + H] calcd for C_20_H_20_N_2_O_5_S, 401.1171; found, 401.1156.

### General procedure for the synthesis of pyrazinones **10a–f**

Similar as described in [26] an oven-dried two-neck round-bottomed flask that had septum in the side arm was cooled to room temperature under a steady stream of nitrogen. The flask was charged with a stir bar, imide **7a–f** (0.5 mmol), and dry dichloromethane (15 mL), and the resulting solution was cooled to 0 °C. To this solution was added TfOH (0.2 mL, 2 mmol) with stirring. After the stipulated time, the contents were warmed to room temperature and methanol (25 mL) was added to the crude reaction mixture, the contents were refluxed for 1 h and then the solvent was evaporated to dryness under reduced pressure. The solid residue was dissolved in water and the aqueous layer was extracted with dichloromethane (2 × 15 mL). The combined organic extracts were washed with brine solution, dried over anhydrous Na_2_SO_4_ and filtered. The solution was concentrated to dryness by using a rotary evaporator. The dried compound was purified through a short silica gel column chromatography using ethyl acetate/hexane mixture (1:1) as eluent to afford the **10a–f** in pure form.

#### 9,10-Dimethoxy-6,7-dihydro-4*H*-pyrazino[2,1-*a*]isoquinolin-4-one (**10a**)

240 mg, (93% yield) yellow solid; mp 132–133 °C; IR (KBr, cm^−1^): 2926, 2850, 1680, 1514, 1354, 1162, 1030, 993, 732; ^1^H NMR (400 MHz, CDCl_3_) δ 8.04 (s, 1H), 7.74 (s, 1H), 7.17 (s, 1H), 6.74 (s, 1H), 4.23–4.17 (m, 2H), 3.94 (s, 3H), 3.93 (s, 3H), 3.04–2.85 (m, 2H); ^13^C NMR (100 MHz, CDCl_3_) δ 155.8, 151.7, 148.9, 145.3, 135.5, 129.0, 119.8, 118.7, 110.8, 107.7, 56.3, 56.2, 38.7, 27.0; HRMS–ESI (*m*/*z*): [M + H] calcd for C_14_H_14_N_2_O_3_, 259.1083; found, 259.1073.

### General procedure for the cyclization of 4-benzylpiperazine-2,6-diones **8a–c**, **8h**, **8i**

Similar as described in [26] an oven-dried two-neck round-bottom flask that had a septum in the side arm was cooled to room temperature under a steady stream of nitrogen. The flask was charged with a stir bar, imide **8a–c**, **8h**, **8i** (0.5 mmol), and dry dichloromethane (10 mL), and the resulting solution was cooled to 0 °C (by using ice). To this solution was added TfOH (0.2 mL, 2 mmol) with stirring. After 6 h the reaction mixture was diluted with methanol (2.5 mL) followed by portion wise addition of NaBH_4_ (2 mmol). The solution was stirred until the color disappeared (Additional NaBH_4_ and MeOH were used if the color persisted for a long time). The solution was evaporated to dryness under reduced pressure. The residue was dissolved in water and extracted with dichloromethane (2 × 20 mL). The organic layer was dried over anhydrous Na_2_SO_4_ and filtered. The solvent was evaporated under vacuum, and the crude product was purified by silica gel column chromatography using ethyl acetate/hexane (1:1) as eluent to afford the **11a–c**, **11h**, and **11i** in pure form.

### 2-Benzyl-1,2,3,6,7,11b-hexahydro-4*H*-pyrazino[2,1-*a*]isoquinolin-4-one (**11h**)

219 mg, (75% yield) yellow liquid; IR (KBr, cm^−1^): 2926, 2824, 1646, 1517, 1457, 1258, 1147, 1033, 744; ^1^H NMR (400 MHz, CDCl_3_) δ 7.28–7.23 (m, 4H), 7.24–7.16 (m, 1H), 7.11–7.08 (m, 2H), 7.07–7.04 (m, 1H), 6.94–6.92 (m, 1H), 4.78 (dd, *J* = 12.0, 8.0 Hz, 1H), 4.70–4.65 (m, 1H), 3.55–3.51 (m, 2H), 3.50–3.41 (m, 2H), 2.91–2.87 (m, 2H), 2.85–2.77 (m, 2H), 2.67–2.62 (m, 1H), 2.30–1.96 (m, 1H); ^13^C NMR (100 MHz, CDCl_3_) δ 166.7, 136.5, 134.9, 132.2, 129.3, 129.2,128.6, 127.7, 127.1, 126.6, 124.7, 61.6, 57.0, 55.7, 55.5, 39.0, 28.7, 20.9; HRMS–ESI (*m*/*z*): [M + H] calcd for C_19_H_20_N_2_O, 293.1654; found, 293.1730.

### Procedure for the synthesis 9,10-dimethoxy-3-phenyl-6,7-dihydro-4*H*-pyrazino[2,1-*a*]isoquinolin-4-one (**13**)

An oven-dried two-neck round-bottom flask that had a septum in the side arm was cooled to room temperature under a steady stream of nitrogen. The flask charged with a stir bar, 3,4-dimethoxypyrazinone **10a** (1.0 mmol) and CH_2_Cl_2_ (5 mL) at 0–5 °C, bromodimethylsulfonium bromide (BDMS) (1.0 mmol, 0.223 g) was added and stirred at room temperature. After 24 h, the reaction mixture was washed with water (2 × 10 mL) and extracted with CH_2_Cl_2_ (2 × 10 mL). The organic layers were combined and dried over anhydrous Na_2_SO_4_. Solvents were removed by evaporation in a rotary evaporator. The crude reaction mixture was purified through silica gel column chromatography using ethyl acetate/hexane, 30:70 as eluent to afford **12a** and **12b** in the ratio 79:21.

The regioisomers of bromopyrazinone (**12a** and **12b**) (1.0 mmol), bis(triphenylphosphine)palladium(II) chloride (10 mol %, 7 mg) in dimethylformamide (2 mL) was added phenylboronic acid (122 mg) and 2 M aqueous sodium carbonate (0.8 mL) under a nitrogen atmosphere. The reaction mixture was heated to 90 °C and stirred for 2 h. The reaction mixture was quenched with water and the mixture was extracted with ethyl acetate. The organic phase was separated, dried over anhydrous Na_2_SO_4_, filtered and concentrated. The crude reaction mixture was purified through a short silica gel column chromatography using ethyl acetate/hexane 10:90 as eluent and afforded **13a** and **13b** in the ratio of 92:8.

### 3-Bromo-9,10-dimethoxy-6,7-dihydro-4*H*-pyrazino[2,1-*a*]isoquinolin-4-one (**12a**)

275 mg, (82% yield) orange yellow liquid; IR (KBr, cm^−1^): 2924, 2856, 1727, 1511, 1456, 1362, 1272, 845; ^1^H NMR (400 MHz, CDCl_3_) δ 7.54 (s, 1H), 7.13 (s, 1H), 6.74 (d, *J* = 9.6 Hz, 1H), 4.26 (t, *J* = 6.4 Hz, 2H), 3.94 (s, 6H), 2.96 (t, *J* = 6.4 Hz, 2H); ^13^C NMR (100 MHz, CDCl_3_) δ 152.7, 152.0, 149.0, 137.9, 136.0, 128.8, 118.5, 118.3, 110.9, 107.6, 56.4, 56.3, 40.9, 27.2; HRMS–ESI (*m*/*z*): [M + H] calcd for C_14_H_13_BrN_2_O_3_, 337.0188; found, 337.0171.

### 9,10-Dimethoxy-3-phenyl-6,7-dihydro-4*H*-pyrazino[2,1-*a*]isoquinolin-4-one (**13a**)

290 mg, (87% yield) yellow liquid ; IR (KBr, cm^−1^): 2924, 2856, 1728, 1595, 1512, 1458, 1216; ^1^H NMR (400 MHz, CDCl_3_) δ 8.35–8.30 (m, 2H), 7.89 (s, 1H), 7.46–7.39 (m, 3H), 7.22 (s, 1H), 6.76 (s, 1H), 4.30 (t, *J* = 6.4 Hz, 2H), 3.95 (s, 3H), 3.94 (s, 3H), 2.98 (t, *J* = 6.4 Hz 2H); ^13^C NMR (100 MHz, CDCl_3_) δ 154.9, 151.5, 149.1, 148.9, 136.6, 134.6, 129.5, 128.8, 128.1, 119.5, 119.3, 110.8, 107.5, 56.4, 56.2, 39.2, 27.3; HRMS–ESI (*m*/*z*): [M + H] calcd for C_20_H_18_N_2_O_3_, 335.1396; found, 335.1383.

### Procedure for the synthesis of praziquantel [2-(cyclohexanecarbonyl)-1,2,3,6,7,11b-hexahydro-4*H*-pyrazino[2,1-*a*]isoquinolin-4-one]

An oven-dried two-neck round-bottom flask that had a septum in the side arm was cooled to room temperature under a steady stream of nitrogen. The flask was charged with a stir bar, 2-benzyl-1,2,3,6,7,11b-hexahydro-4*H*-pyrazino[2,1-*a*]isoquinolin-4-one (292 mg, 1 mmol), palladium hydroxide on charcoal (30 mg) and ethyl acetate (15 mL). The reaction flask was degassed and filled with hydrogen gas twice through a rubber bladder and the contents were stirred under hydrogen atmosphere. After 24 h, the reaction mixture was filtered through a small bed of celite and washed with ethyl acetate (2 × 10 mL). The solvents were removed by evaporation using a rotary evaporator. The crude product was used in the next step without further purification

An oven-dried two-neck round-bottom flask that had a septum in the side arm was cooled to room temperature under a steady stream of nitrogen. The flask was charged with a stir bar, cyclohexanecarboxylic acid (0.14 mL, 1.1 mmol) and carbonyldiimidazole (194 mg, 1.2 mmol) and dry THF (10 mL). The contents were stirred to obtain a clear solution. To this solution was added dropwise a solution of 1,2,3,6,7,11b-hexahydro-4*H*-pyrazino[2,1-*a*]isoquinolin-4-one (200 mg, 2.32 mmol) in dry THF and the resulting solution was stirred at room temperature for 24 h. The solution was concentrated to dryness using a rotary evaporator. The crude reaction mixture was purified by a short silica gel column chromatography using ethyl acetate/hexane (10:90) as eluent to furnished praziquantel (**1**) in 249 mg, (80% yield) as a white solid; mp 129–131 °C; IR (KBr, cm^−1^): 2928, 2856, 1660, 1452, 1211, 1039, 755, 656; ^1^H NMR (400 MHz, CDCl_3_) δ 7.21–7.13 (m, 4H), 5.08 (dd, *J* = 13.2, 2.4 Hz, 1H), 4.79–4.72 (m, 2H), 4.39 (d, *J* = 17.2 Hz, 1H), 4.0 (d, *J* = 13.2 Hz, 1H), 2.91–2.69 (m, 4H), 2.40 (t, *J* = 11.6 Hz, 1H), 1.73–1.65 (m, 5H), 1.53–1.43 (m, 2H), 1.23-1.17 (m, 3H); ^13^C NMR (100 MHz, CDCl_3_) δ 174.9, 164.6, 134.6, 132.6, 129.2, 127.4, 126.9, 125.4, 54.9, 48.9, 45.1, 42.8, 40.7, 39.1, 29.1, 28.9, 28.8, 28.6, 25.7, 25.6, 25.3, 20.8; HRMS–ESI (*m*/*z*): [M + H] calcd for C_19_H_24_N_2_O_2_, 313.1916; found, 313.1905.

## Supporting Information

File 1 ^1^H and ^13^C NMR spectra of synthesized compounds.
