# Supplementary material for: Brønsted acid-mediated cyclization–dehydrosulfonylation/reduction sequences: An easy access to pyrazinoisoquinolines and pyridopyrazines
Source: Beilstein J Org Chem. 2017 Mar 7;13:428–40. doi: 10.3762/bjoc.13.46 (PMC5355893; doi:10.3762/bjoc.13.46)
Supplement: File 1 — 1H and 13C NMR spectra of synthesized compounds. [file Beilstein_J_Org_Chem-13-428-s001.pdf]

Supporting Information

for

Brønsted acid-mediated cyclization–dehydrosulfonylation/ reduction sequences: An easy access to pyrazinoisoquinolines and pyridopyrazines

Ramana Sreenivasa Rao and Chinnasamy Ramaraj Ramanathan*

Address: Department of Chemistry, Pondicherry University, Puducherry – 605 014, India.

Email: Chinnasamy Ramaraj Ramanathan* - crrnath.che@pondiuni.edu.in

*Corresponding author

^{1}H and ^{13}C NMR spectra of synthesized compounds

S. No.	Table of contents	Page No.
1	General information	S3
2	Analytical data for compounds **7b–h**	S4–S6
3	Analytical data for compounds **8b–c**, **8h**, and **8i**	S7–S8
4	Analytical data for compounds **9b**–**g**	S8–S11
5	Analytical data for compounds **10b**–**f**	S11–S13
6	Analytical data for compounds **11a**–**c** and **11i**	S13–S15
7	X-ray crystal structure of compound **9b** and **10a**	S16
8	References	S17
9	HRMS spectra of an aliquot generated from cyclization reaction of **7c**	S17
10	Copies of ^{1}H and ^{13}C NMR spectra	S18

1. General information

Melting points reported in this paper are uncorrected and were determined using BUCHI M-560, Buchi Labortechnik AG, Switzerland. Infrared spectra were recorded on Thermo Nicolet 6700 FT-IR Spectrophotometer and are reported in frequency of absorption (cm^{-1}). Mass spectra were measured with Agilent-6530 B Q-TOF (ESI-HRMS), ^{1}H and ^{13}C NMR were recorded on Bruker AVANCE 400 spectrometer. NMR spectra for all the samples were measured in $CDCl_3$ using TMS as an internal standard. The chemical shifts are expressed in δ ppm down field from the signal of internal TMS.

Trifluoromethanesulfonic acid and iminodiacetic acid were purchased from Sigma-Aldrich and used without further purification. Aromatic/hetero aldehydes were also purchased from Sigma-Aldrich and purified (liquid sample) by distillation under reduced pressure. β-Arylethylamines were prepared from corresponding aromatic/hetero aldehydes using reported procedures.[1-5] Solvents used for the reactions were dried using standard procedures. Analytical thin layer chromatographic tests were carried out using pre-coated aluminum TLC plates. The spots were visualized by short exposure to iodine vapour or UV light. Column chromatography was carried out using Merck silica gel (100–200 mesh). All the glassware were pre-dried at 120 °C for 6 h and assembled while hot and cooled under stream of dry nitrogen gas. In all experiments, round bottom flasks of appropriate size were used.

2. Analytical data for compounds 7b–h

1-(3,5-Dimethoxyphenethyl)-4-(phenylsulfonyl)piperazine-2,6-dione (**7b**)

310 mg, (74% yield) white solid; m.p. 135-136 ºC; IR (KBr, cm^{-1}): 3003, 2937, 2835, 1740, 1687, 1590, 1515, 1447, 1354, 1268, 1171, 1114, 1026, 960, 851; ^{1}H NMR (400 MHz, $CDCl_3$): δ 7.94 (dd, *J* = 8.0, 1.2 Hz, 2H), 7.65 (s, 1H), 7.89-7.57 (m, 2H), 6.33-6.31 (m, 1H), 6.30-6.28 (m, 2H), 4.13 (s, 4H), 3.76 (s, 6H), 3.65-3.60 (m, 2H), 2.44-2.40 (m, 2H); ^{13}C (100 MHz, $CDCl_3$): 166.3, 160.9, 140.8, 135.6, 134.1, 129.9, 127.6, 107.0, 104.6, 98.8, 55.4, 48.9, 40.5, 34.0; HRMS-ESI (*m/z*): Calculated for $C_{20}H_{22}N_2O_6S$ (M+H): 419.1277, Found (M+H): 419.1271.

1-(3-Methoxyphenethyl)-4-(phenylsulfonyl)piperazine-2,6-dione (**7c**)

272 mg, (70% yield) colorless solid; m.p. 131-132 ºC; IR (KBr, cm^{-1}): 3005, 2936, 2832, 1741, 1687, 1608, 1444, 1354, 1172, 1112, 9666, 755, 575; ^{1}H NMR (400 MHz, $CDCl_3$): δ 7.78-7.79 (m, 2H), 7.66-7.62 (m, 1H), 7.59-7.55 (m, 2H), 7.17 (d, *J* = 8.0 Hz, 2H), 6.97-6.66 (m, 2H), 4.11 (s, 4H), 3.77 (s, 3H), 3.64-3.60 (m, 2H), 2.52–2.41 (m, 2H); ^{13}C NMR (100 MHz, $CDCl_3$): δ 166.3, 159.8, 139.3, 135.6, 134.1, 130.0, 129.6, 127.6, 121.1, 114.5, 112.2, 55.2, 48.8, 40.5, 33.7; HRMS-ESI (*m/z*): Calculated for $C_{19}H_{20}N_2O_5S$ (M+H): 389.1171, Found (M+H): 389.1140.

1-Phenethyl-4-(phenylsulfonyl)piperazine-2,6-dione (**7h**)

243 mg, (68% yield) colorless solid; m.p. 127-128 ºC; IR (KBr, cm^{-1}): 2937, 2835, 1741, 1687, 1608, 1443, 1354, 964, 752, 573; ^{1}H NMR (400 MHz, $CDCl_3$): δ 7.80-7.78 (m,

2H), 7.66-7.62 (m, 1H), 7.58-7.55 (m, 2H), 7.29- 7.25 (m, 2H), 7.20-7.18 (m, 1H), 7.15-7.13 (m, 2H), 4.12 (s, 4H), 3.66-3.62 (m, 2H), 2.52–2.48 (m, 2H); ^{13}C NMR (100 MHz, $CDCl_3$): δ 166.3, 137.8, 135.6, 134.1, 130.0, 129.8, 128.6, 127.6, 126.8, 48.8, 40.7, 33.7; HRMS-ESI (*m/z*): Calculated for $C_{18}H_{18}N_2O_4S$ (M+H): 359.1066, Found (M+H): 359.1072.

1-(2-(1*H*-Indol-3-yl)ethyl)-4-(phenylsulfonyl)piperazine-2,6-dione (**7g**)

309 mg, (78% yield) yellow solid; m.p. 117-118 ºC; IR (KBr, cm^{-1}): 3403, 3121, 1742, 1687, 1541, 1486, 1447, 1398, 1325, 1171, 1060, 933, 835, 741, 660, 574; ^{1}H NMR (400 MHz, $CDCl_3$): δ 7.97 (s, 1H), 7.81-7.78 (m, 2H), 7.64-7.60 (m, 2H), 7.57-7.53 (m, 2H), 7.33 (d, *J* = 8.0 Hz, 1H), 7.17 (td, *J* = 7.6, 1.2 Hz, 1H), 7.1 (td, *J* = 8.0, 7.6 Hz, 1H), 6.99 (d, *J* = 2.4 Hz, 1H), 4.11 (s, 4H), 3.73-3.69 (m, 2H), 2.69-2.65 (m, 2H); ^{13}C NMR (100 MHz, $CDCl_3$): δ 166.5, 134.1, 130.0, 127.7, 122.3, 119.7, 118.9, 111.3, 49.0, 40.2, 23.5; HRMS-ESI (*m/z*): Calculated for $C_{20}H_{19}N_3O_4S$ (M+H): 398.1175, Found (M+H): 398.1167.

1-(2-(Benzo[*b*]thiophen-2-yl)ethyl)-4-(phenylsulfonyl)piperazine-2,6-dione (**7f**)

298 mg, (72% yield) yellow liquid; IR (KBr, cm^{-1}): 2920, 1680, 1335, 1154, 960, 738, 623, 578; ^{1}H NMR (400 MHz, $CDCl_3$): δ 7.3 (d, *J* = 7.2 Hz, 2H), 7.7 (d, *J* = 7.6 Hz, 1H), 7.59 (dd, *J* = 6.8, 4.4 Hz, 2H), 7.53-7.49 (m, 2H), 7.26-7.18 (m, 2H), 6.94 (s, 1H), 4.08 (s, 4H), 3.72-3.68 (m, 2H), 2.76–2.72 (m, 2H); ^{13}C NMR (100 MHz, $CDCl_3$): δ 166.4, 140.7, 140.0, 139.6, 139.4, 135.6, 134.0, 129.8, 127.7, 124.4, 124.0, 123.2, 122.3, 49.0,

40.0, 31.1, 28.6; HRMS-ESI (*m/z*): Calculated for $C_{20}H_{18}N_2O_4S_2$ (M+H): 415.0786, Found (M+H): 415.0760.

4-(Phenylsulfonyl)-1-(3,4,5-trimethoxyphenethyl)piperazine-2,6-dione (**7d**)

304 mg, (68% yield) yellow solid; m.p. 125-126 ºC; IR (KBr, cm^{-1}): 1739, 1687, 1647, 1588, 1507, 1446, 1335, 1240, 1170, 1125, 1004, 954,763, 737, 627, 523; ^1H NMR (400 MHz, $CDCl_3$): δ 7.80 (d, *J* = 7.6 Hz, 2H), 7.66-7.67 (m, 1H), 7.60-7.56 (m, 2H), 6.36 (s, 2H), 4.12 (s, 4H), 3.84 (s, 6H), 3.80 (s, 3H), 3.67-3.63 (m, 2H), 2.56-2.39 (m, 2H); ^{13}C NMR (100 MHz, $CDCl_3$): δ 166.5, 153.4, 136.9, 135.5, 134.2, 133.5, 130.0, 127.7, 105.8, 60.9, 56.2, 48.9, 40.8, 34.2; HRMS-ESI (*m/z*): Calculated for $C_{21}H_{24}N_2O_7S$ (M+H): 449.1382, Found (M+H): 449.1372.

4-(Phenylsulfonyl)-1-(2-(thiophen-2-yl)ethyl)piperazine-2,6-dione (**7e**)

273 mg, (75% yield) yellow liquid ; IR (KBr, cm^{-1}): 3007, 1743, 1688, 1616, 1345, 1283, 1170, 970, 854, 757, 627, 574; ^1H NMR (400 MHz, $CDCl_3$): δ 7.81-7.78 (m, 2H), 7.68-7.64 (m, 1H), 7.61-7.55 (m, 2H), 7.12 (dd, *J* = 5.2, 1.2 Hz, 1H), 6.90 (dd, *J* = 5.2, 3.6 Hz, 1H), 6.78-6.77 (m, 1H), 4.13 (s, 4H), 3.71-3.67 (m, 2H), 2.74 (m, 2H); ^{13}C NMR (100 MHz, $CDCl_3$): δ 166.4, 139.7, 135.7, 134.2, 130.0, 127.7, 127.1, 125.7, 124.2, 49.0, 40.6, 27.7; HRMS-ESI (*m/z*): Calculated for $C_{16}H_{16}N_2O_4S_2$ (M+H): 365.0630, Found (M+H): 365.0627.

3. Analytical data for compounds 8b–c, 8h and 8i

4-Benzyl-1-(3,5-dimethoxyphenethyl)piperazine-2,6-dione (**8b**)

258 mg, (70% yield) yellow liquid; IR (KBr, cm^{-1}): 2942, 2833, 1736, 1683, 1589, 1470, 1442, 1351, 1267, 1168, 1083, 1007, 748, 632; ^{1}H NMR (400 MHz, $CDCl_3$): δ 7.33-7.30 (m, 1H), 7.29-7.26 (m, 2H), 7.26-7.22 (m, 2H), 6.94 (t, J = 8.0 Hz, 1H), 6.78-6.74 (m, 2H), 3.97-3.93 (m, 2H), 3.82 (s, 3H), 3.80 (s, 3H), 3.56 (s, 2H), 3.38 (s, 4H), 2.86-2.82 (m, 2H); ^{13}C NMR (100 MHz, $CDCl_3$): δ 169.9, 152.9, 147.7, 135.7, 132.4, 129.2, 128.8, 128.0, 123.8, 122.6, 111.0, 60.9, 60.8, 56.5, 55.8, 39.7, 28.2; HRMS-ESI (*m/z*): Calculated for $C_{21}H_{24}N_2O_4$ (M+H): 369.1814, Found (M+H): 369.1806.

4-Benzyl-1-(3-methoxyphenethyl)piperazine-2,6-dione (**8c**)

230 mg, (68% yield) yellow liquid; IR (KBr, cm^{-1}): 2955, 2834, 1734, 1682, 1596, 1350, 1261, 1162, 1043, 699; ^{1}H NMR (400 MHz, $CDCl_3$): δ 7.27-7.21 (m, 3H), 7.18-7.16 (m, 2H), 7.14-7.10 (m, 1H), 6.75 (d, J = 7.6 Hz, 1H), 6.70-6.66 (m, 2H), 3.91-3.87 (m, 2H), 3.70 (s, 3H), 3.48 (s, 2H), 3.28 (s, 4H), 2.75-2.71 (m, 2H); ^{13}C NMR (100 MHz, $CDCl_3$): δ 169.8, 159.7, 139.9, 135.5, 129.5, 129.0, 128.7, 128.0, 121.4, 114.5, 112.1, 60.6, 56.2, 55.1, 40.1, 33.9; HRMS-ESI (m/z): Calculated for $C_{20}H_{22}N_2O_3$ (M+H): 339.1709, Found (M+H): 339.1683.

4-Benzyl-1-phenethylpiperazine-2,6-dione (**8h**)

216 mg, (70% yield) yellow liquid; IR (KBr, cm^{-1}): 2926, 1692, 1449, 1369, 1031, 747, 698; ^{1}H NMR (400 MHz, $CDCl_3$): δ 7.31-7.27 (m, 1H), 7.27-7.22 (m, 2H), 7.22-7.17 (m, 1H), 7.17-7.12 (m, 2H), 4.02-3.83 (m, 2H), 3.51 (s, 2H), 3.30 (s, 4H), 2.88-2.68 (m, 2H);

^{13}C NMR (100 MHz, $CDCl_3$): δ 169.9, 138.4, 135.5, 129.2, 129.0, 128.8, 128.6, 128.0, 126.7, 60.6, 56.4, 40.4, 34.0; HRMS-ESI (*m/z*): Calculated for $C_{19}H_{20}N_2O_2$ (M+H): 309.1603, Found (M+H): 309.1590.

4-Benzyl-1-(2-(1-ethyl-1*H*-pyrrol-2-yl)ethyl)piperazine-2,6-dione (**8i**)

254 mg, (78% yield) yellow liquid; IR (KBr, cm^{-1}): 3098, 2973, 2780, 2247, 1743, 1684, 1439, 1351, 1148, 1068, 965, 922, 709, 629; ^{1}H NMR (400 MHz, $CDCl_3$): δ 7.38-7.35 (m, 1H), 7.34-7.31 (m, 2H), 7.30-7.28 (m, 3H), 6.63 (dd, *J* = 2.8, 2.0 Hz, 1H), 6.09-6.07 (m, 1H), 5.86 (dd, *J* = 3.6, 1.6 Hz, 1H), 3.96-3.92 (m, 4H), 3.61 (s, 2H), 3.40 (s, 4H), 2.82-2.79 (m, 2H), 1.31 (t, *J* = 7.3 Hz, 3H); ^{13}C NMR (100 MHz, CDCl3): δ 169.9, 135.4, 129.1, 128.7, 128.1, 128.0, 119.6, 107.1, 107.0, 60.8, 56.4, 41.0, 38.8, 24.5, 16.8; HRMS-ESI (*m/z*): Calculated for $C_{19}H_{23}N_3O_2$ (M+H): 326.1869, Found (M+H): 326.1863.

4. Analytical data for compounds 9b–g

9,11-Dimethoxy-2-(phenylsulfonyl)-2,3,6,7-tetrahydro-4*H*-pyrazino[2,1-*a*]isoquinolin-4-one (**9b**)

348 mg, (87% yield) colorless liquid; IR (KBr, cm^{-1}): 2926, 2845, 1680, 1514, 1356, 1162, 1032, 825, 733; ^{1}H NMR (400 MHz, $CDCl_3$): δ 7.83 (dd, *J* = 7.2, 1.6 Hz, 2H), 7.61-7.57 (m, 1H), 7.53-7.49 (m, 1H), 7.16 (s, 1H), 6.42 (d, *J* = 2.0 Hz, 1H), 6.27 (d, *J* = 2.4 Hz, 1H), 4.18 (s, 2H), 3.89 (s, 3H), 3.81 (s, 3H), 3.47-3.44 (m, 2H), 2.57-2.54 (m, 2H); ^{13}C NMR (100 MHz, $CDCl_3$): δ 162.3, 138.0, 133.4, 129.1, 127.4, 104.6, 97.8, 55.8, 55.5, 48.3, 38.1, 29.9; HRMS-ESI (*m/z*): Calculated for $C_{20}H_{20}N_2O_5S$ (M+H): 401.1171, Found (M+H): 401.1156.

9-Methoxy-2-(phenylsulfonyl)-2,3,6,7-tetrahydro-4*H*-pyrazino[2,1-*a*]isoquinolin-4-one (9c)

337 mg, (91% yield) colorless liquid; IR (KBr, cm^{-1}): 2933, 1682, 1494, 1456, 1361, 1280, 1172, 1024, 725; ^{1}H NMR (400 MHz, $CDCl_3$): δ 7.81 (dd, , *J* = 8.0, 0.8 Hz, 2H), 7.60-7.56 (m, 1H), 7.49 (td, *J* = 6.8, 1.6 Hz, 2H), 7.426 (d, *J* = 8.8 Hz, 1H), 6.85-6.79 (m, 1H), 6.63 (d, *J* = 2.78 Hz, 1H), 6.6 (d, *J* = 4.4 Hz, 1H), 4.18 (s, 2H), 3.89 (s, 3H), 3.80 (s, 4H), 3.53-3.45 (m, 2H), 2.58-2.55 (m, 2H); ^{13}C NMR (100 MHz, $CDCl_3$): δ 161.9, 152.9, 145.7, 136.9, 133.6, 129.3, 128.4, 127.3, 126.5, 121.4, 119.6, 111.4, 104.4, 60.8, 56.0, 48.3, 37.9, 21.9; HRMS-ESI (*m/z*): Calculated for $C_{19}H_{18}N_2O_4S$ (M+H): 371.1066, Found (M+H): 371.1035.

9,10,11-Trimethoxy-2-(phenylsulfonyl)-2,3,6,7-tetrahydro-4*H*-pyrazino[2,1-*a*]isoquinolin-4-one (9d)

365 mg, (85% yield) colorless liquid; IR (KBr, cm^{-1}): 2936, 2842, 1677, 1592, 1461, 1354, 1162, 1100, 1032, 726, 573; ^{1}H NMR (400 MHz, $CDCl_3$): δ 7.81-7.79 (m, 2H), 7.59-7.55 (m, 1H), 7.52-7.47 (m, 2H), 7.23 (s, 1H), 6.40 (s, 1H), 4.11 (s, 2H), 3.83 (s, 3H), 3.81 (s, 6H), 3.53-3.46 (m, 2H), 2.56-2.53 (m, 2H); ^{13}C NMR (100 MHz, $CDCl_3$): δ 162.0, 153.1, 151.3, 141.7, 136.8, 133.5, 131.1, 129.2, 127.3, 121.0, 114.4, 109.6, 107.1, 61.1, 60.4, 56.0, 48.1, 38.3, 29.4; HRMS-ESI (*m/z*): Calculated for $C_{21}H_{22}N_2O_6S$ (M+H): 431.1277, Found (M+H): 431.1269.

2-(Phenylsulfonyl)-2,3,6,7-tetrahydro-4*H*-thieno[3',2':3,4]pyrido[1,2-*a*]pyrazin-4-one (**9e**)

294 mg, (85% yield) yellow liquid; IR (KBr, cm^{-1}): 2925, 2857, 1689, 1456, 1359, 1168, 960, 726, 684; ^1H NMR (400 MHz, $CDCl_3$): δ 7.83-7.80 (m, 2H), 7.6-7.56 (m, 1H), 7.53-7.49 (m, 2H), 7.16 (d, *J* = 5.2 Hz, 1H), 7.10 (d, *J* = 5.2 Hz, 1H), 6.60 (s, 1H), 4.15 (s, 2H), 3.63 (t, *J* = 6.0 Hz, 2H), 2.83 (t, *J* = 6.0 Hz, 2H); ^{13}C NMR (100 MHz, $CDCl_3$): δ 161.9, 136.8, 134.7, 133.6, 129.3, 127.7, 127.2, 125, 124.9, 122.1, 104.9, 48.6, 38.9, 24.0; HRMS-ESI (*m/z*): Calculated for $C_{16}H_{14}N_2O_3S_2$ (M+H): 347.0524, Found (M+H): 347.0526.

2-(Phenylsulfonyl)-2,3,6,7-tetrahydro-4*H*-benzo[4',5']thieno[3',2':3,4]pyrido[1,2-*a*]pyrazin-4-one (**9f**)

285 mg, (72% yield) yellow solid; m.p. 105-106 ºC; IR (KBr, cm^{-1}):2923, 2855, 1657, 1576, 1479, 1268, 1023, 803; ^1H NMR (400 MHz, $CDCl_3$): δ 7.89 (d, *J* = 8.0 Hz, 1H), 7.8-7.43 (m, 3H), 7.51 (d, *J* = 7.2 Hz 1H), 7.46-7.43 (m, 3H), 7.34-7.31 (m, 1H), 6.95 (s, 1H), 4.17 (s, 2H), 3.64-3.61 (m, 2H), 2.84-2.81 (m, 2H); ^{13}C NMR (100 MHz, $CDCl_3$): δ 162.5, 139.1, 138.4, 136.8, 135.1, 133.7, 129.4, 127.3, 125.6, 124.8, 124.0, 123.1, 122.3, 122.2, 105.9, 48.3, 38.4, 29.8; HRMS-ESI (*m/z*): Calculated for $C_{20}H_{16}N_2O_3S_2$ (M+H): 397.0681, Found (M+H): 397.0678.

2-(Phenylsulfonyl)-2,6,7,12-tetrahydropyrazino[1',2':1,2]pyrido[3,4-*b*]indol-4(3H)-one (**9g**)

326 mg, (86% yield) yellow oily liquid; IR (KBr, cm^{-1}): 3319, 2924, 1679, 1358, 1169, 989, 737, 687, 639; ^1H NMR (400 MHz, $CDCl_3$): δ 8.46 (s, 1H), 7.89-7.87 (m, 2H), 7.62-7.58 (m, 1H), 7.55-7.50 (m, 2H), 7.45 (d, *J* = 8.0 Hz, 1H), 7.35 (dt, *J* = 8.4, 0.8 Hz, 1H), 7.24-7.22 (m, 1H), 7.26-7.21 (m, 1H), 7.12 (td, *J* = 7.2, 1.2 Hz, 1H), 4.23 (s, 2H), 3.68 (t, *J* = 6.0 Hz, 2H), 2.84 (t, *J* = 6.0 Hz, 2H); ^{13}C NMR (100 MHz, $CDCl_3$): δ 161.7, 137.6, 133.9, 129.5, 127.3, 126.4, 125.4, 124.3, 122.6, 120.5, 118.9, 112.6, 111.4, 102.6, 49.0, 39.6, 20.3; HRMS-ESI (*m/z*): Calculated for $C_{20}H_{17}N_3O_3S$ (M+H): 380.1069, Found (M+H): 380.1045.

5. Analytical data for compounds 10b–f

9,11-Dimethoxy-6,7-dihydro-4*H*-pyrazino[2,1-*a*]isoquinolin-4-one (**10b**)

211 mg, (82% yield) yellow solid; m.p. 137-138 ºC; IR (KBr, cm^{-1}): 2926, 2850, 1680, 1514, 1354, 1162, 1030, 993, 732; ^1H NMR (400 MHz, $CDCl_3$): δ 8.06 (s, 1H), 7.75 (s, 1H), 7.17 (s, 1H), 6.75 (s, 1H), 4.22-4.17 (m, 2H), 3.94 (s, 6H), 2.96-2.93 (m, 2H); ^{13}C NMR (100 MHz, $CDCl_3$): δ 155.9, 151.8, 149.0, 145.3, 135.6, 129.0, 119.9, 118.8, 110.8, 107.7, 56.4, 56.2, 38.8, 27.0; HRMS-ESI (*m/z*): Calculated for $C_{14}H_{14}N_2O_3$ (M+H): 259.1083, Found (M+H): 259.1070.

9-Methoxy-6,7-dihydro-4*H*-pyrazino[2,1-*a*]isoquinolin-4-one (**10c**)

194 mg, (85% yield) yellow solid; m.p. 120-138 ºC; IR (KBr, cm^{-1}): 2965, 2923, 2853, 1737, 1651, 1584, 1562, 1464, 1421, 1334, 1190, 825, 799, 734, 738, 552; ^1H NMR

(400 MHz, $CDCl_3$): δ 8.03 (s, 1H), 7.76 (s, 1H), 7.68 (d, *J* = 8.7 Hz, 1H), 6.88 (dd, *J* = 8.7, 2.6 Hz, 1H), 6.77 (d, *J* = 2.6 Hz, 1H), 4.21-4.18 (m, 2H), 3.84 (s, 3H), 3.00-2.96 (m, 2H); ^{13}C NMR (100 MHz, $CDCl_3$): δ 161.8, 159.9, 155.8, 145.1, 137.2, 135.7, 129.7, 129.4, 127.4, 127.0, 119.8, 119.3, 114.5, 114.1, 113.4, 55.6, 38.7, 27.7; HRMS-ESI (*m/z*): Calculated for $C_{13}H_{12}N_2O_2$ (M+H): 229.0977, Found (M+H): 229.0969.

9,10,11-Trimethoxy-6,7-dihydro-4*H*-pyrazino[2,1-*a*]isoquinolin-4-one (**10d**)

229 mg, (80% yield) yellow solid; m.p. 127-128 ºC; IR (KBr, cm^{-1}): 2926, 2853, 1661, 1596, 1338, 1190, 1116, 838, 810; ^{1}H NMR (400 MHz, $CDCl_3$): δ 8.37 (s, 1H), 8.08 (s, 1H), 6.58 (s, 1H), 4.18 (m, 2H), 3.95-3.84 (m, 9H), 2.94-2.83 (m, 2H); ^{13}C NMR (100 MHz, $CDCl_3$): δ 156.0, 155.5, 153.0, 145.2, 142.1, 133.1, 133.0, 124.6, 113.3, 107.1, 61.2, 56.2, 38.8, 29.8, 28.7; HRMS-ESI (*m/z*): Calculated for $C_{15}H_{16}N_2O_4$ (M+H): 289.1188, Found (M+H): 289.1171.

6,7-Dihydro-4*H*-thieno[3',2':3,4]pyrido[1,2-*a*]pyrazin-4-one (**10e**)

184 mg, (90% yield) yellow solid; m.p. 132-133 ºC; IR (KBr, cm^{-1}): 2923, 2855, 1656, 1580, 1457, 1269, 1097; ^{1}H NMR (400 MHz, $CDCl_3$): δ 8.02 (s, 1H), 7.58 (s, 1H), 7.26 (s, 2H), 4.27 (t, *J* = 6.8 Hz, 2H), 3.09 (t, *J* = 6.8 Hz, 2H); ^{13}C NMR (100 MHz, $CDCl_3$): δ 155.8, 146.1, 139.1, 132.5, 128.0, 125.6, 123.0, 119.5, 39.3, 29.7, 22.7; HRMS-ESI (m/z): Calculated for $C_{10}H_8N_2OS$ (M+H): 205.0436, Found (M+H): 205.0419.

6,7-Dihydro-4*H*-benzo[4',5']thieno[3',2':3,4]pyrido[1,2-*a*]pyrazin-4-one (**10f**)

193 mg, (76% yield) yellow solid; m.p. 156-157 ºC; IR (KBr, cm^{-1}): 2925, 2855, 1657, 1576, 1479, 1268, 1023, 803, 734; ^1H NMR (400 MHz, $CDCl_3$): δ 8.14 (s, 1H), 8.13-8.06 (m, 1H), 7.88 (s, 1H), 7.54-7.50 (m, 1H), 7.45-7.43 (m, 1H), 7.43-7.41 (m, 1H), 4.39-4.36 (m, 2H), 3.24-3.21 (m, 2H); ^{13}C NMR (100 MHz, $CDCl_3$): δ 156.0, 146.6, 143.3, 139.3, 134.9, 132.5, 126.0, 125.3, 123.3, 122.7, 122.5, 120.5, 38.8, 24.2; HRMS-ESI (*m/z*): Calculated for $C_{14}H_{10}N_2OS$ (M+H): 255.0592, Found (M+H): 255.0583.

6. Analytical data for compounds 11a–c and 11i

2-Benzyl-9,10-dimethoxy-1,2,3,6,7,11b-hexahydro-4*H*-pyrazino[2,1-*a*]isoquinolin-4-one (**11a**)

300 mg, (85% yield) colorless liquid; IR (KBr, cm^{-1}): 2931, 2841, 1648, 1484, 1454, 1329, 1279, 1089, 744, 697; ^1H NMR (400 MHz, $CDCl_3$): δ 7.35-7.26 (m, 4H), 7.32-7.26 (m, 1H), 6.62 (s, 1H), 6.50 (s, 1H), 4.85-4.79 (m, 2H), 3.86 (s, 3H), 3.80 (s, 3H), 3.72 (d, J = 12.8 Hz, 1H), 3.56-3.52 (m, 1H), 3.48-3.43 (m, 2H), 2.95-2.78 (m, 3H), 2.63 (dd, J = 15.2, 2.8 Hz, 1H), 2.38 (dd, J = 11.6, 10.0 Hz, 1H); ^{13}C NMR (100 MHz, $CDCl_3$): δ 165.5, 148.2, 147.9, 136.8, 129.2, 128.6, 127.7, 127.5, 126.2, 111.9, 108.0, 61.9, 57.1, 56.5, 56.3, 56.0, 55.5, 39.0, 28.5; HRMS-ESI (*m/z*): Calculated for $C_{21}H_{24}N_2O_3$ (M+H): 353.1865, Found (M+H): 353.1861.

2-Benzyl-9,11-dimethoxy-1,2,3,6,7,11b-hexahydro-4H-pyrazino[2,1-a]isoquinolin-4-one (11b)

290 mg, (82% yield) colorless. liquid ; IR (KBr, cm^{-1}): 2923, 2841, 1650, 1458, 1279, 1088, 744, 697; ^1H NMR (400 MHz, $CDCl_3$): δ 7.28-7.26 (m, 4H), 7.25-7.19 (m, 1H), 6.69 (dd, *J* = 16.8, 8.8 Hz, 2H), 4.72 (dd, *J* = 11.2, 4.4 Hz, 2H), 3.8 (s, 3H), 3.73 (s, 3H), 3.59-3.51 (m, 2H), 3.47-3.43 (m, 1H), 3.41-3.37 (m, 1H), 2.88 (d, *J* = 4.4 Hz, 1H), 2.84 (d, *J* = 6.0 Hz, 1H), 2.74-2.68 (m, 2H), 2.26 (dd, *J* = 11.2, 10.0 Hz, 1H); ^{13}C NMR (100 MHz, $CDCl_3$): δ 166.5, 151.4, 146.6, 136.9, 129.7, 129.2, 128.6, 127.8, 127.7, 120.3, 110.8, 61.9, 60.4, 57.3, 56.3, 55.9, 55.1, 38.6, 23.1; HRMS-ESI (*m/z*): Calculated for $C_{21}H_{24}N_2O_3$ (M+H): 353.1865, Found (M+H): 353.1861.

2-Benzyl-9-methoxy-1,2,3,6,7,11b-hexahydro-4H-pyrazino[2,1-a]isoquinolin-4-one (11c)

273 mg, (85% yield) colorless liquid; IR (KBr, cm^{-1}): 2928, 2840, 1648, 1501, 1457, 1294, 1246, 1157, 1034, 744, 710, 600; ^1H NMR (400 MHz, $CDCl_3$): δ 7.35 (d, *J* = 4.4 Hz, 4H), 7.32-7.29 (m, 1H), 6.94 (d, *J* = 8.8 Hz, 1H), 6.74 (dd, *J* = 8.8, 2.4 Hz, 1H), 6.67 (d, *J* = 2.4 Hz, 1H), 4.83-4.74 (m, 2H), 3.78 (s, 3H), 3.62 (d, *J* = 4.0 Hz, 2H), 3.51-3.45 (m, 2H), 2.96-2.85 (m, 3H), 2.73-2.69 (m, 1H), 2.34-2.29 (m, 1H); ^{13}C NMR (100 MHz, $CDCl_3$): δ 166.6, 158.5, 136.9, 136.5, 129.2, 128.7, 127.7, 126.7, 126.0, 113.8, 113.0, 61.9, 57.3, 56.2, 55.4, 55.3, 38.9, 29.2; HRMS-ESI (*m/z*): Calculated for $C_{20}H_{22}N_2O_2$ (M+H): 323.1760, Found (M+H): 323.1775.

2-Benzyl-8-ethyl-2,3,6,7,8,10b-hexahydropyrrolo[3',2':3,4]pyrido[1,2-a]pyrazin-4(1H)-one (**11i**)

259 mg, (84% yield) green liquid; IR (KBr, cm^{-1}): 2923, 2853, 1647, 1455, 1351, 1284, 1092 (br) 705, 477; ^{1}H NMR (400 MHz, $CDCl_3$): δ 7.27-7.26 (m, 5H), 7.19 (s, 1H), 6.50 (d, J = 2.8 Hz, 1H), 5.77 (d, J = 2.8 Hz, 1H), 4.98-4.93 (m, 1H), 4.64 (dd, J = 10.4, 4.8 Hz, 1H), 3.75-3.69 (m, 2H), 3.57-3.41 (m, 3H), 3.31-3.26 (m, 1H), 2.85-2.75 (m, 2H), 2.67-2.65 (m, 1H), 2.17 (d, J = 10.4 Hz, 1H), 1.29-1.25 (m, 3H); ^{13}C NMR (100 MHz, $CDCl_3$): δ 166.8, 129.2, 128.6, 127.6, 125.7, 119.5, 116.4, 61.9, 57.6, 56.4, 53.7, 41.1, 38.6, 29.8, 21.9, 16.5; HRMS-ESI (*m/z*): Calculated for $C_{19}H_{23}N_3O$ (M+H): 310.1919, Found (M+H): 310.1913.

7. X-ray crystal structure of compound 9b and 10a

**Crystal data:**	**9b**	**10a**
Empirical formula	$C_{41}H_{42}N_4O_{10}S_2Cl_2$	$C_{14}H_{14}N_2O_3$
Formula weight	885.80	258.27
Temperature/K	298	298(2)
Crystal system	monoclinic	orthorhombic
Space group	$P2_1$	*P*bca
a/Å	8.0605(10)	9.3599(7)
b/Å	26.933(3)	9.3599(7)
c/Å	9.4487(11)	17.9438(15)
α/°	90.00	90
β/°	92.587(12)	90
γ/°	90.00	90
Volume/Å^3	2049.2(4)	2453.1(3)
Z	2	8
ρ_{calc}mg/mm^3	1.436	1.399
m/mm^{-1}	0.324	0.100
F(000)	924.0	1088.0
Crystal size/mm^3	0.5 × 0.25 × 0.2	0.3 × 0.2 × 0.03
2Θ range for data collection	7.172 to 58.332°	7.194 to 58.37°
Index ranges	$-10 \leq h \leq 8$, $-28 \leq k \leq 36$, $-12 \leq l \leq 12$	$-18 \leq h \leq 19$, $-12 \leq k \leq 10$, $-21 \leq l \leq 24$
Reflections collected	11174	10303
Independent reflections	7170 [R_{int} = 0.0766, R_{sigma} = 0.1184]	2961 [R_{int} = 0.0359, R_{sigma} = 0.0429]
Data/restraints/parameters	7170/1/547	2961/0/174
Goodness-of-fit on F^2	1.097	1.021
Final R indexes [I>=2σ (I)]	R_1 = 0.0861, wR_2 = 0.2042	R_1 = 0.0503, wR_2 = 0.1099
Final R indexes [all data]	R_1 = 0.1508, wR_2 = 0.2538	R_1 = 0.1020, wR_2 = 0.1352
Largest diff. peak/hole / e Å^{-3}	0.40/-0.37	0.17/-0.22

8. References

1. Merchant, J. R.; Mountwala, A. J. *J. Org. Chem.* **1958**, *23*, 1774
2. Sinhababu, A. K.; Borchardt, R. T.*Tetrahedron Lett.* **1983**, *24*, 227
3. Trost, B. M. ; Yeh, V. S. C. ;.Ito, H. ; Bremeyer, N. *Org. Lett*. **2002**, *4*, 2621
4. Pei, Z. ; Li, L.; Geldern, T.W. ; Longenecker, K. ; Pireh, D.; Stewart, K. D. ; Backes, B. J. ; Lai, C.; Lubben, T. H. ;. Ballaron, S. J ; Beno, D. W. A.; Grote, A. J. ; Sham, H. L. ; Trevillyan, J. M. *J. Med. Chem.* **2007**, *50*, 1983.
5. Gigant, N.; Claveau, E.; Bouyssou, P.; Gillaizeau, I. *Org. Lett*. **2012**, *7*, 3679.

9. HRMS spectra of an aliquot generated from cyclization reaction of 7c

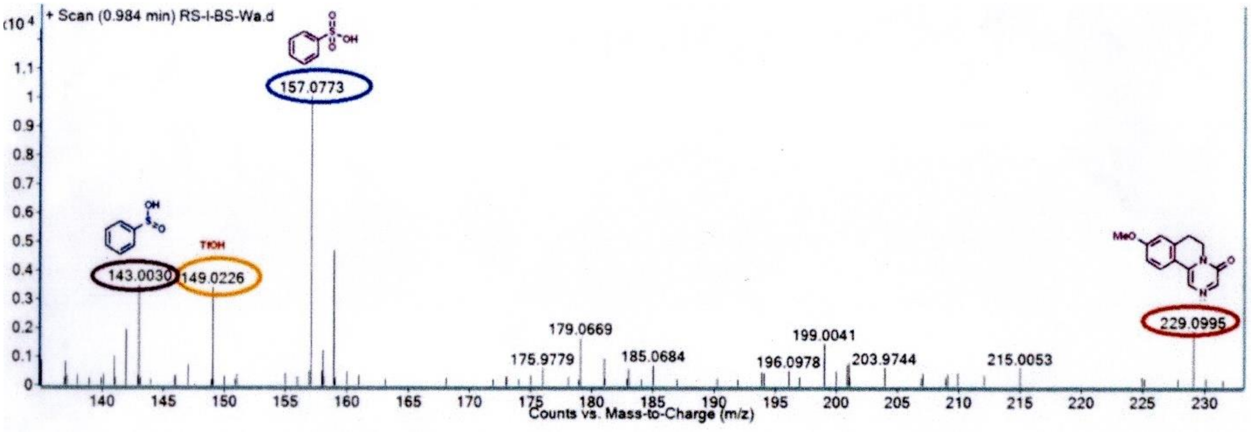

10. Copies of ^{1}H and ^{13}C NMR spectra

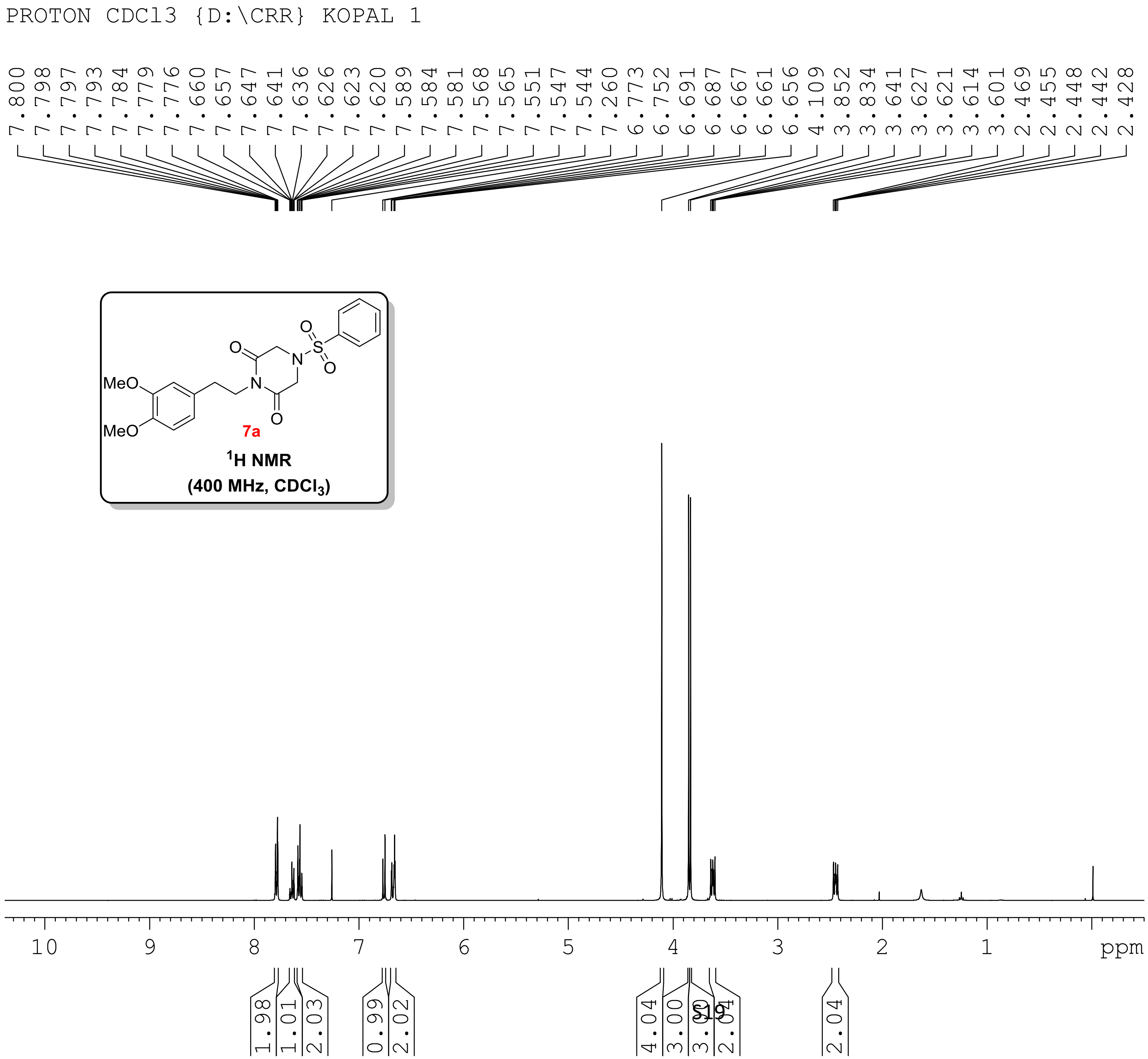

Current Data Parameters
NAME RS-I-PZQ-imide-S
EXPNO 1
PROCNO 1

F2 - Acquisition Parameters
Date_ 20130710
Time 12.05
INSTRUM spect
PROBHD 5 mm BBO BB-1H
PULPROG zg30
TD 65536
SOLVENT CDCl3
NS 16
DS 2
SWH 8223.685 Hz
FIDRES 0.125483 Hz
AQ 3.9846387 sec
RG 228
DW 60.800 usec
DE 6.00 usec
TE 296.7 K
D1 1.00000000 sec
TD0 1

======== CHANNEL f1 ========
NUC1 1H
P1 14.50 usec
PL1 -0.90 dB
SFO1 400.1324710 MHz

F2 - Processing parameters
SI 32768
SF 400.1300056 MHz
WDW EM
SSB 0
LB 0.30 Hz
GB 0
PC 1.00

C13CPD CDCl3 {D:\CRR} KOPAL 1

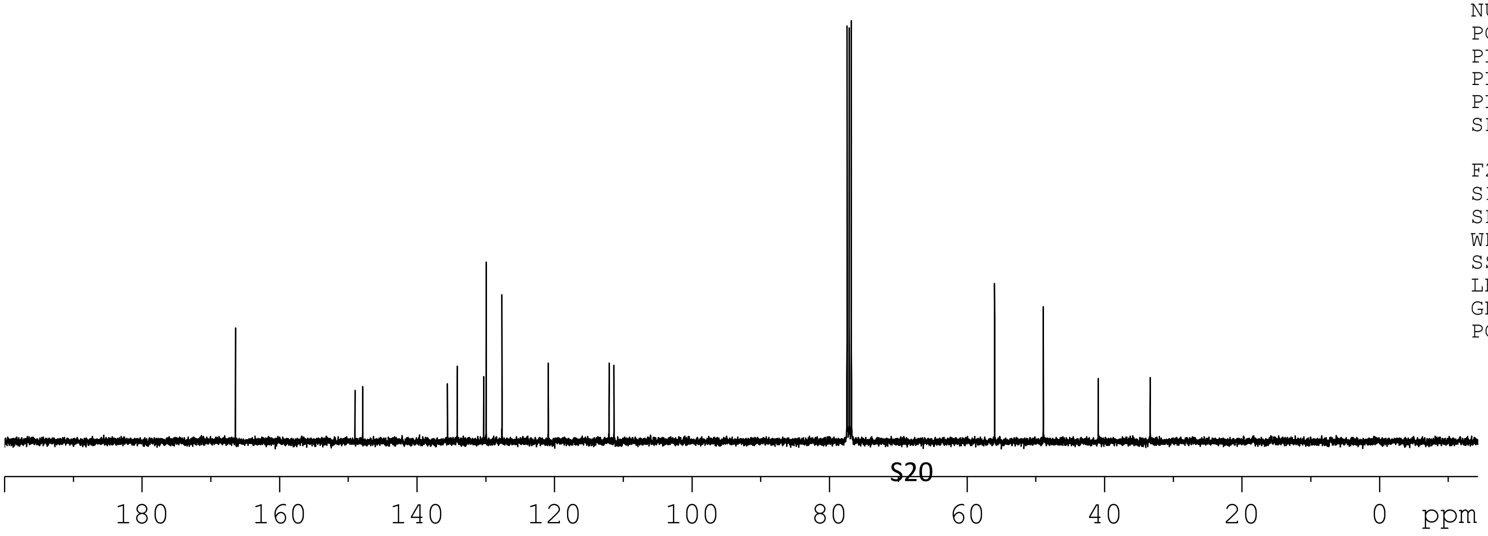

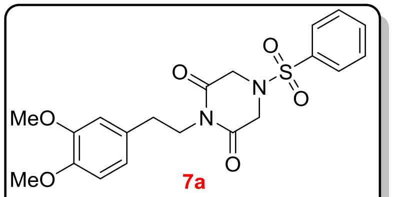

7a

^{13}C NMR

(100 MHz, $CDCl_3$)

```
Current Data Parameters
NAME     RS-I-PZQ-imide-S
EXPNO                   2
PROCNO                  1

F2 - Acquisition Parameters
Date_            20130710
Time                12.21
INSTRUM             spect
PROBHD   5 mm BBO BB-1H
PULPROG           zgpg30
TD                 65536
SOLVENT            CDCl3
NS                   256
DS                     4
SWH            24038.461 Hz
FIDRES          0.366798 Hz
AQ             1.3631988 sec
RG                   724
DW                20.800 usec
DE                  6.00 usec
TE                 297.0 K
D1            2.00000000 sec
d11           0.03000000 sec
DELTA         1.89999998 sec
TD0                    1

======== CHANNEL f1 ========
NUC1                 13C
P1                  9.50 usec
PL1                -0.60 dB
SFO1         100.6228298 MHz

======== CHANNEL f2 ========
CPDPRG2          waltz16
NUC2                  1H
PCPD2              90.00 usec
PL12               14.96 dB
PL13               15.60 dB
PL2                -0.90 dB
SFO2         400.1316005 MHz

F2 - Processing parameters
SI                 32768
SF           100.6127573 MHz
WDW                   EM
SSB                    0
LB                  1.00 Hz
GB                     0
PC                  1.40
```

PROTON CDCl3 {D:\CRR} KOPAL 1

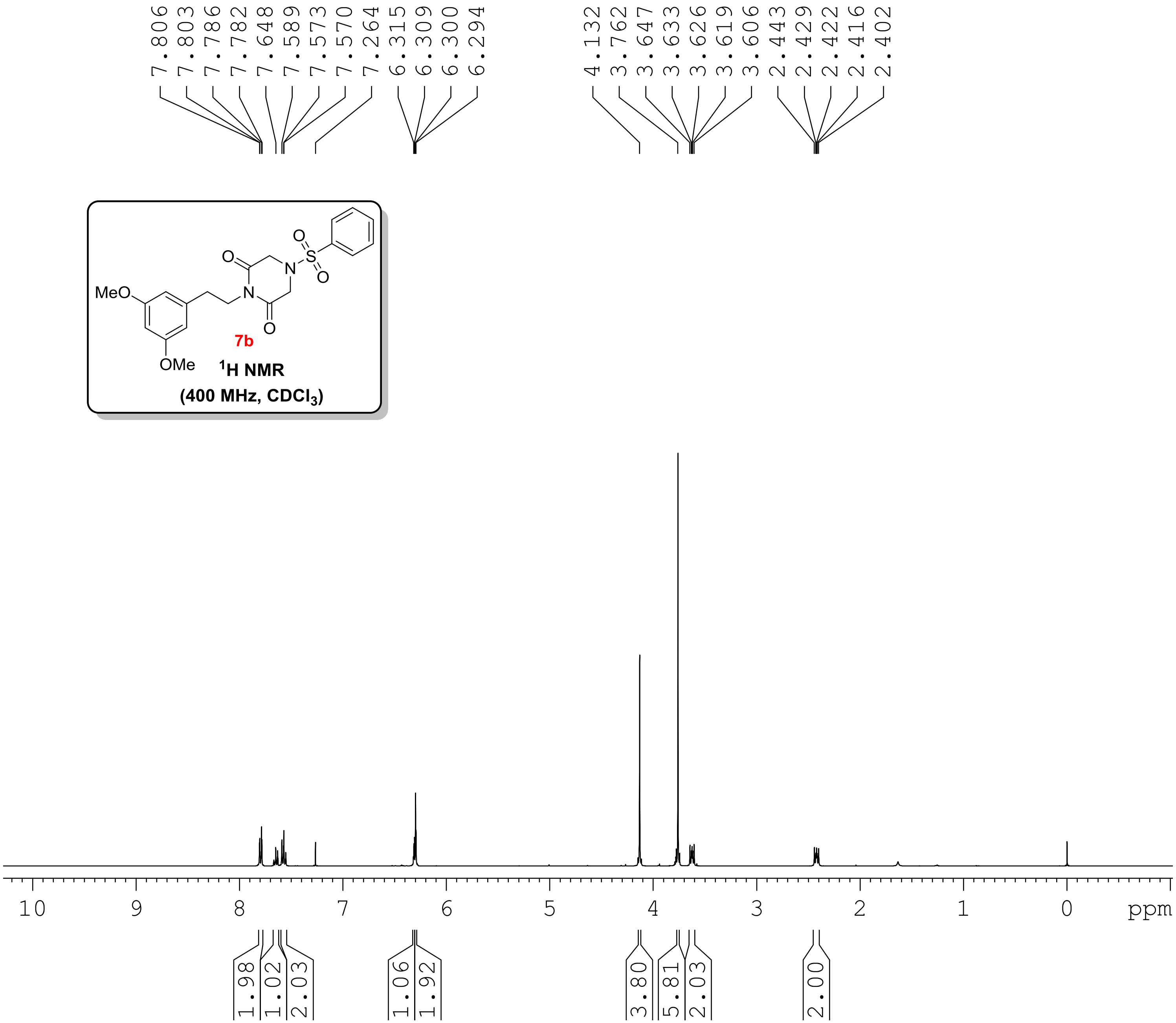


```
Current Data Parameters
NAME       RS-I-3,5-PZQ-IM
EXPNO                    1
PROCNO                   1

F2 - Acquisition Parameters
Date_             20130904
Time                 10.44
INSTRUM              spect
PROBHD     5 mm BBO BB-1H
PULPROG               zg30
TD                   65536
SOLVENT              CDCl3
NS                      16
DS                       2
SWH               8223.685 Hz
FIDRES            0.125483 Hz
AQ               3.9846387 sec
RG                     228
DW                  60.800 usec
DE                    6.00 usec
TE                   295.0 K
D1              1.00000000 sec
TD0                      1

======== CHANNEL f1 ========
NUC1                    1H
P1                   14.50 usec
PL1                  -0.90 dB
SFO1           400.1324710 MHz

F2 - Processing parameters
SI                   32768
SF             400.1300039 MHz
WDW                     EM
SSB                      0
LB                    0.30 Hz
GB                       0
PC                    1.00
```

C13CPD CDCl3 {D:\CRR} KOPAL 1

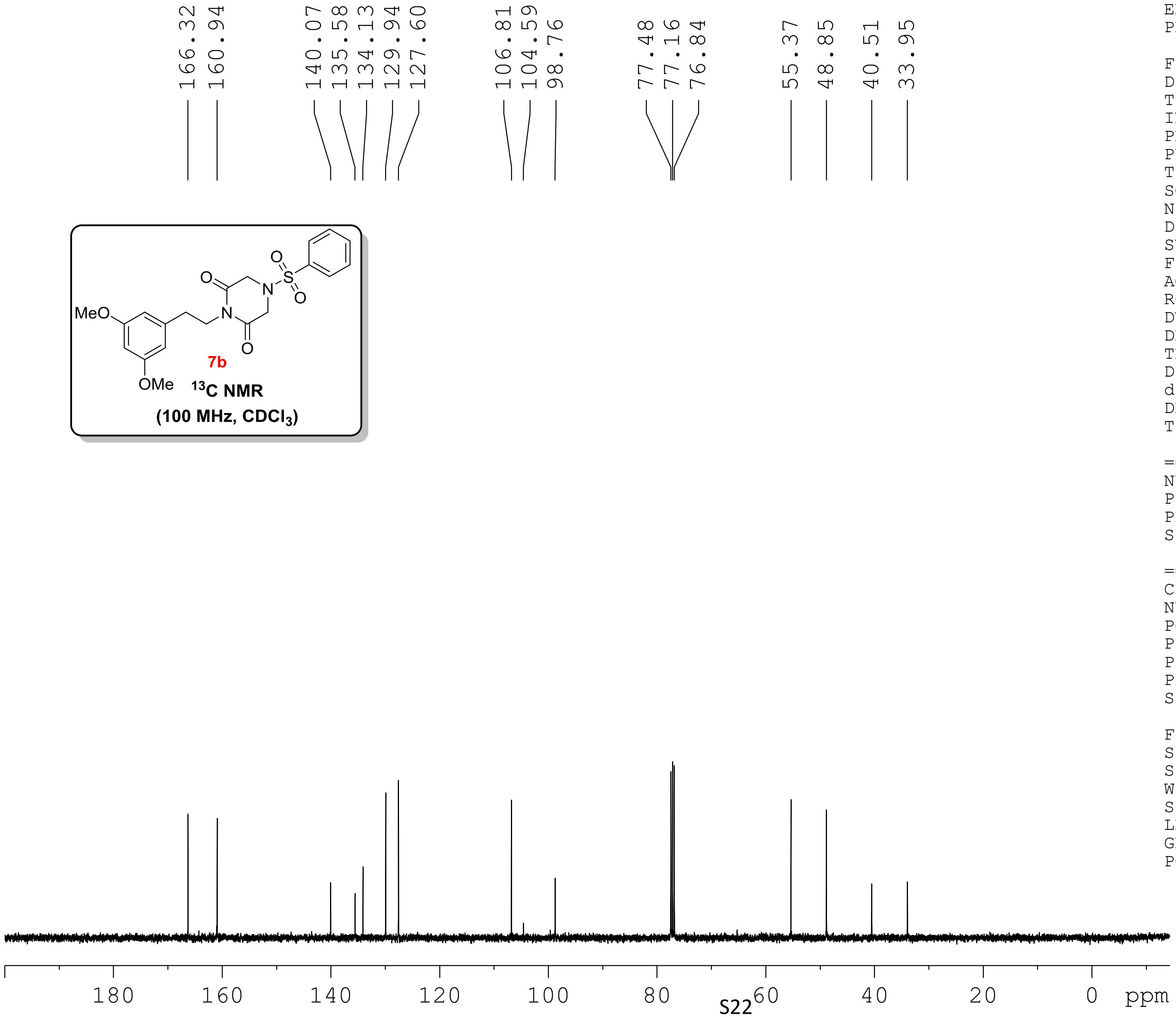

Current Data Parameters
NAME RS-I-3,5-PZQ-IMIDE
EXPNO 3
PROCNO 1

F2 - Acquisition Parameters
Date_ 20130902
Time 15.09
INSTRUM spect
PROBHD 5 mm BBO BB-1H
PULPROG zgpg30
TD 65536
SOLVENT CDCl3
NS 68
DS 4
SWH 24038.461 Hz
FIDRES 0.366798 Hz
AQ 1.3631988 sec
RG 50.8
DW 20.800 usec
DE 6.00 usec
TE 296.4 K
D1 2.00000000 sec
d11 0.03000000 sec
DELTA 1.89999998 sec
TD0 1

======== CHANNEL f1 ========
NUC1 13C
P1 9.50 usec
PL1 -0.60 dB
SFO1 100.6228298 MHz

======== CHANNEL f2 ========
CPDPRG2 waltz16
NUC2 1H
PCPD2 90.00 usec
PL12 14.96 dB
PL13 15.60 dB
PL2 -0.90 dB
SFO2 400.1316005 MHz

F2 - Processing parameters
SI 32768
SF 100.6122841 MHz
WDW EM
SSB 0
LB 1.00 Hz
GB 0
PC 1.40

PROTON CDCl3 {D:\CRR} guest 1

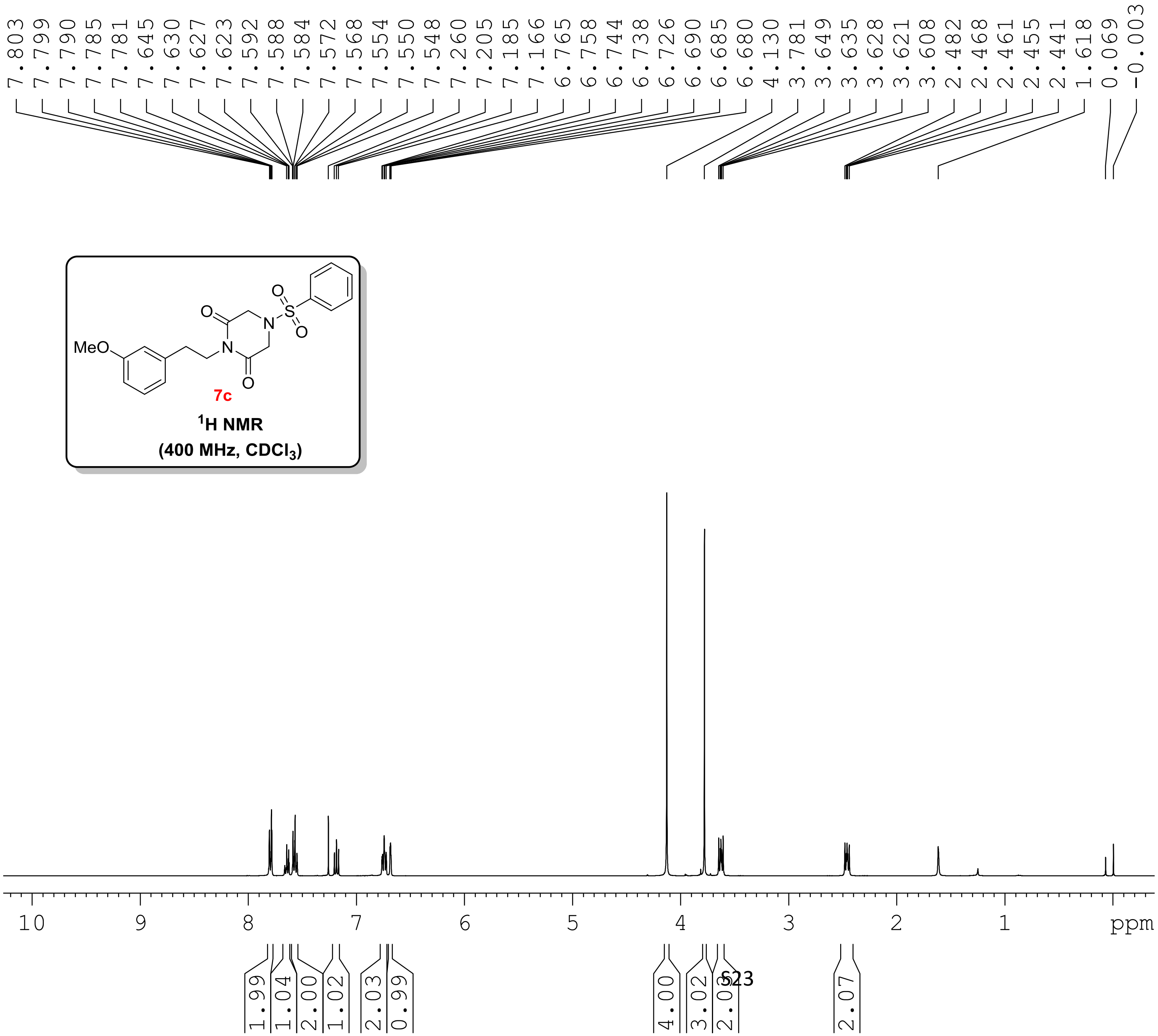

Current Data Parameters
NAME RS-I-3-OME-PZQ-IN
EXPNO 2
PROCNO 1

F2 - Acquisition Parameters
Date_ 20150215
Time 10.33
INSTRUM spect
PROBHD 5 mm DUL 13C-1
PULPROG zg30
TD 65536
SOLVENT CDCl3
NS 12
DS 2
SWH 8223.685 Hz
FIDRES 0.125483 Hz
AQ 3.9846387 sec
RG 161
DW 60.800 usec
DE 6.00 usec
TE 293.0 K
D1 1.00000000 sec
TD0 1

======== CHANNEL f1 ========
NUC1 1H
P1 11.42 usec
PL1 -3.00 dB
SFO1 400.1324710 MHz

F2 - Processing parameters
SI 32768
SF 400.1300051 MHz
WDW EM
SSB 0
LB 0.30 Hz
GB 0
PC 1.00

C13CPD CDCl3 {D:\CRR} guest 1

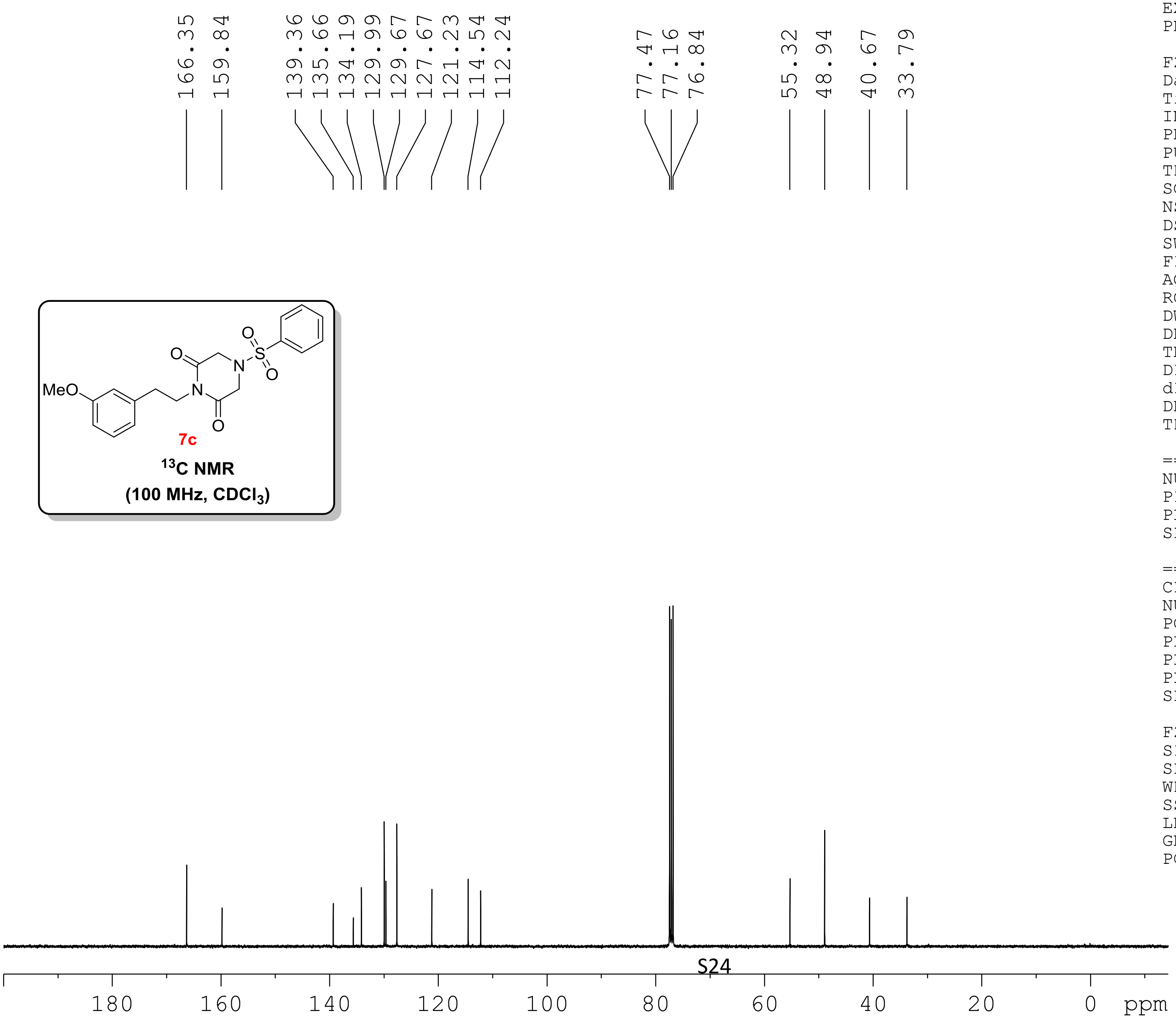

Current Data Parameters
NAME RS-I-3-OME-PZQ-IN
EXPNO 1
PROCNO 1

F2 - Acquisition Parameters
Date_ 20150215
Time 11.50
INSTRUM spect
PROBHD 5 mm DUL 13C-1
PULPROG zgpg30
TD 65536
SOLVENT CDCl3
NS 1024
DS 4
SWH 24038.461 Hz
FIDRES 0.366798 Hz
AQ 1.3631988 sec
RG 32
DW 20.800 usec
DE 6.00 usec
TE 294.4 K
D1 2.00000000 sec
d11 0.03000000 sec
DELTA 1.89999998 sec
TD0 1

======== CHANNEL f1 ========
NUC1 13C
P1 9.15 usec
PL1 0.00 dB
SFO1 100.6228298 MHz

======== CHANNEL f2 ========
CPDPRG2 waltz16
NUC2 1H
PCPD2 90.00 usec
PL12 14.90 dB
PL13 14.90 dB
PL2 -3.00 dB
SFO2 400.1316005 MHz

F2 - Processing parameters
SI 32768
SF 100.6127567 MHz
WDW EM
SSB 0
LB 1.00 Hz
GB 0
PC 1.40

PROTON CDCl3 {D:\CRR} KOPAL 1

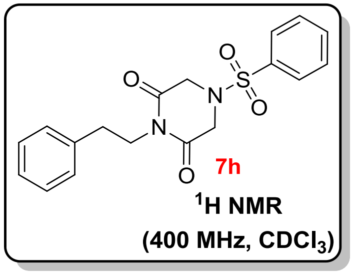

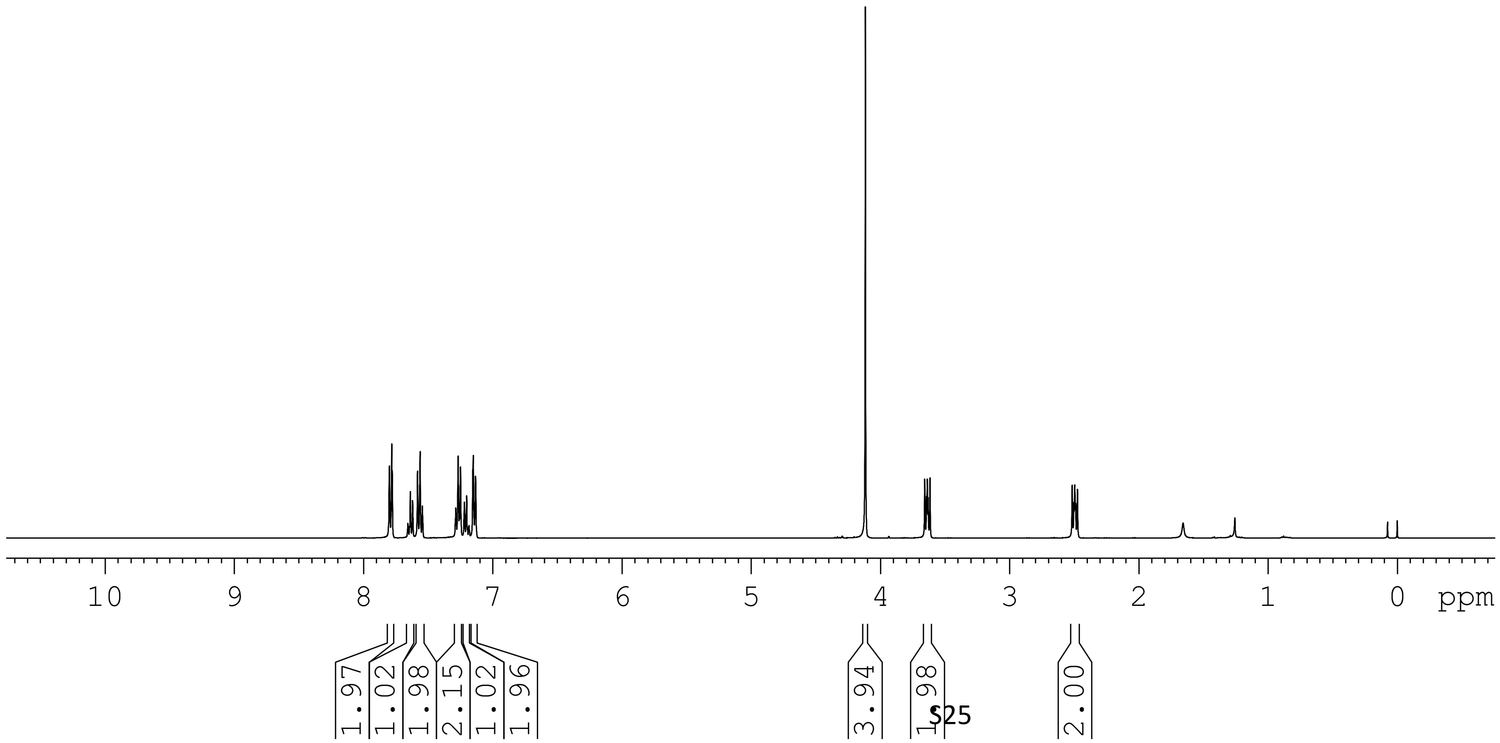

Current Data Parameters
NAME RS-I-UN-PZQ-IMIDE
EXPNO 1
PROCNO 1

F2 - Acquisition Parameters
Date_ 20130821
Time 14.55
INSTRUM spect
PROBHD 5 mm BBO BB-1H
PULPROG zg30
TD 65536
SOLVENT CDCl3
NS 16
DS 2
SWH 8223.685 Hz
FIDRES 0.125483 Hz
AQ 3.9846387 sec
RG 128
DW 60.800 usec
DE 6.00 usec
TE 297.6 K
D1 1.00000000 sec
TD0 1

======== CHANNEL f1 ========
NUC1 1H
P1 14.50 usec
PL1 -0.90 dB
SFO1 400.1324710 MHz

F2 - Processing parameters
SI 32768
SF 400.1300056 MHz
WDW EM
SSB 0
LB 0.30 Hz
GB 0
PC 1.00

C13CPD CDCl3 {D:\CRR} KOPAL 1

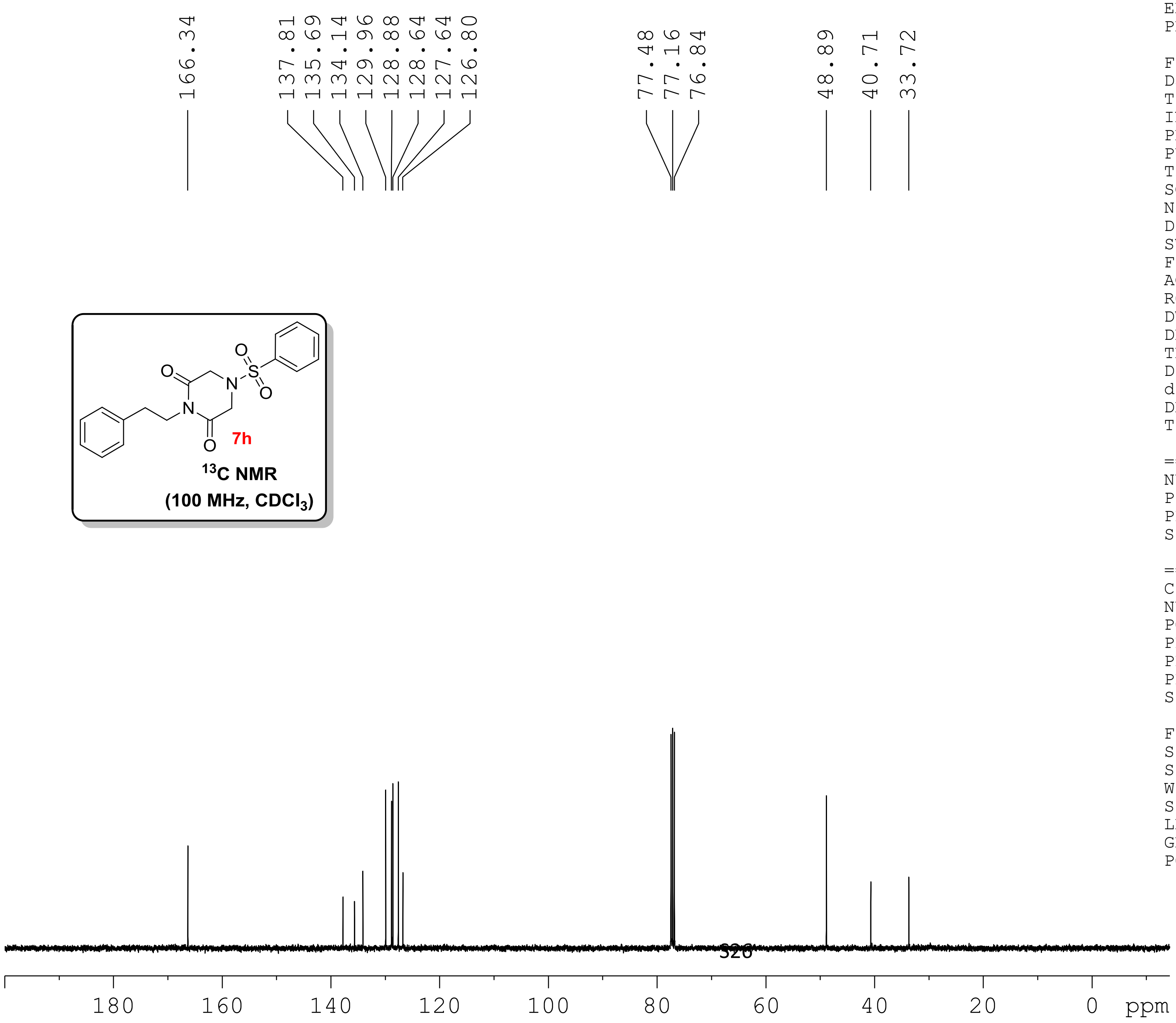

7h

^{13}C NMR

(100 MHz, $CDCl_3$)

Current Data Parameters
NAME RS-I-UN-PZQ-IMIDE
EXPNO 2
PROCNO 1

F2 - Acquisition Parameters
Date_ 20130821
Time 15.01
INSTRUM spect
PROBHD 5 mm BBO BB-1H
PULPROG zgpg30
TD 65536
SOLVENT CDCl3
NS 120
DS 4
SWH 24038.461 Hz
FIDRES 0.366798 Hz
AQ 1.3631988 sec
RG 57
DW 20.800 usec
DE 6.00 usec
TE 298.0 K
D1 2.00000000 sec
d11 0.03000000 sec
DELTA 1.89999998 sec
TD0 1

======== CHANNEL f1 ========
NUC1 13C
P1 9.50 usec
PL1 -0.60 dB
SFO1 100.6228298 MHz

======== CHANNEL f2 ========
CPDPRG2 waltz16
NUC2 1H
PCPD2 90.00 usec
PL12 14.96 dB
PL13 15.60 dB
PL2 -0.90 dB
SFO2 400.1316005 MHz

F2 - Processing parameters
SI 32768
SF 100.6127596 MHz
WDW EM
SSB 0
LB 1.00 Hz
GB 0
PC 1.40

PROTON CDCl3 {D:\CRR} KOPAL 1

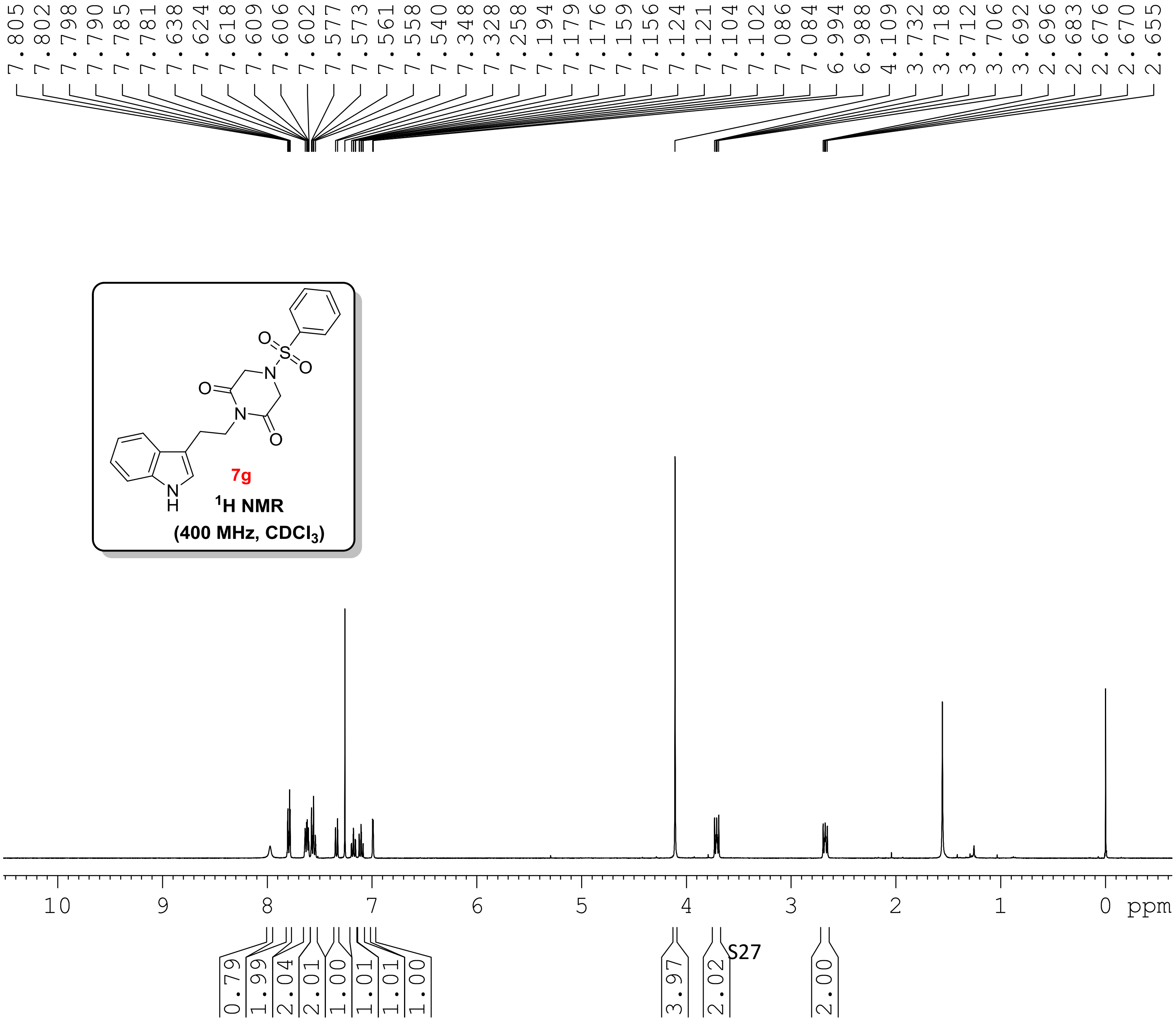

Current Data Parameters
NAME RS-I-TRY-PZQ
EXPNO 1
PROCNO 1

F2 - Acquisition Parameters
Date_ 20130802
Time 11.10
INSTRUM spect
PROBHD 5 mm BBO BB-1H
PULPROG zg30
TD 65536
SOLVENT CDCl3
NS 16
DS 2
SWH 8223.685 Hz
FIDRES 0.125483 Hz
AQ 3.9846387 sec
RG 1150
DW 60.800 usec
DE 6.00 usec
TE 297.8 K
D1 1.00000000 sec
TD0 1

======== CHANNEL f1 ========
NUC1 1H
P1 14.50 usec
PL1 -0.90 dB
SFO1 400.1324710 MHz

F2 - Processing parameters
SI 32768
SF 400.1300063 MHz
WDW EM
SSB 0
LB 0.30 Hz
GB 0
PC 1.00

C13CPD CDCl3 {D:\CRR} KOPAL 1

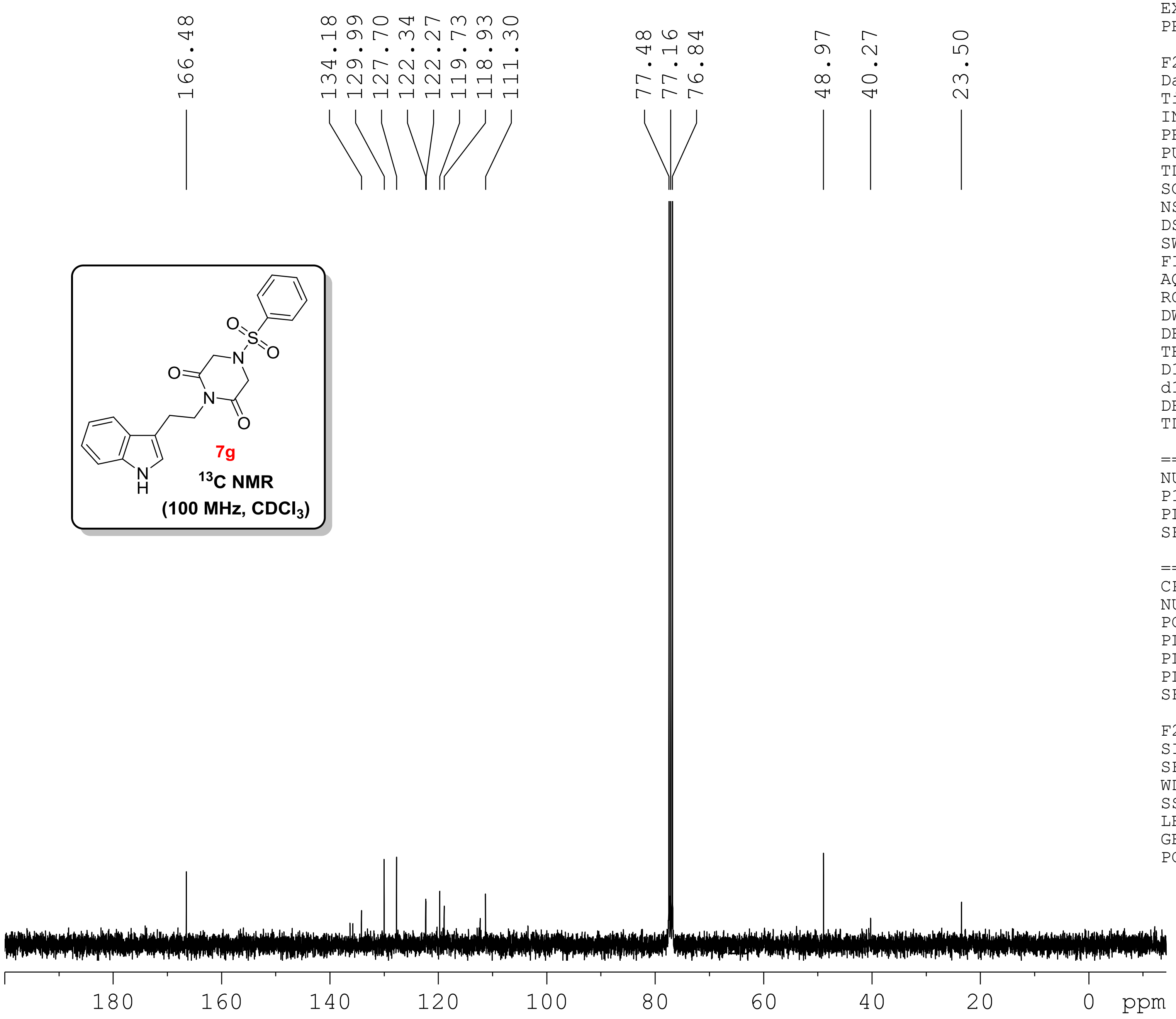


```
Current Data Parameters
NAME           RS-I-TRY-PZQ
EXPNO                     2
PROCNO                    1

F2 - Acquisition Parameters
Date_              20130802
Time                  11.25
INSTRUM               spect
PROBHD    5 mm BBO BB-1H
PULPROG             zgpg30
TD                    65536
SOLVENT               CDCl3
NS                      256
DS                        4
SWH            24038.461 Hz
FIDRES          0.366798 Hz
AQ             1.3631988 sec
RG                      575
DW                20.800 usec
DE                  6.00 usec
TE                  298.2 K
D1            2.00000000 sec
d11           0.03000000 sec
DELTA         1.89999998 sec
TD0                       1

======== CHANNEL f1 ========
NUC1                    13C
P1                  9.50 usec
PL1                -0.60 dB
SFO1         100.6228298 MHz

======== CHANNEL f2 ========
CPDPRG2             waltz16
NUC2                     1H
PCPD2              90.00 usec
PL12               14.96 dB
PL13               15.60 dB
PL2                -0.90 dB
SFO2         400.1316005 MHz

F2 - Processing parameters
SI                    32768
SF           100.6127531 MHz
WDW                      EM
SSB                       0
LB                  1.00 Hz
GB                        0
PC                     1.40
```

PROTON CDCl3 {D:\CRR} KOPAL 1

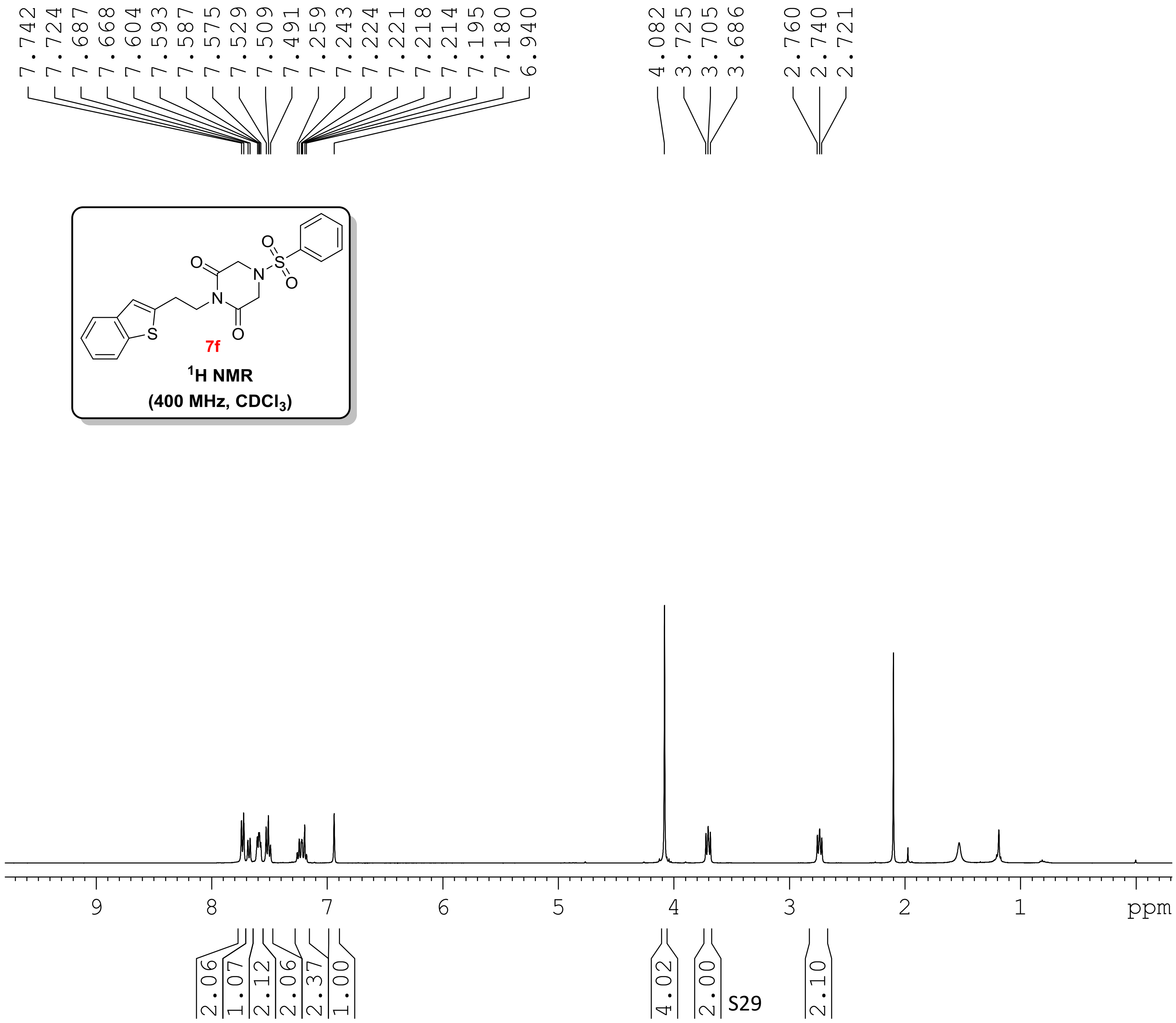


```
Current Data Parameters
NAME       RS-I-BT-PZQ-IM
EXPNO                   1
PROCNO                  1

F2 - Acquisition Parameters
Date_            20150724
Time                11.32
INSTRUM             spect
PROBHD   5 mm DUL 13C-1
PULPROG              zg30
TD                  65536
SOLVENT             CDCl3
NS                     16
DS                      2
SWH              8223.685 Hz
FIDRES           0.125483 Hz
AQ              3.9846387 sec
RG                    228
DW                 60.800 usec
DE                   6.00 usec
TE                  296.2 K
D1             1.00000000 sec
TD0                     1

======== CHANNEL f1 ========
NUC1                   1H
P1                  11.42 usec
PL1                 -3.00 dB
SFO1          400.1324710 MHz

F2 - Processing parameters
SI                  32768
SF            400.1300314 MHz
WDW                    EM
SSB                     0
LB                   0.30 Hz
GB                      0
PC                   1.00
```

C13CPD CDCl3 {D:\CRR} KOPAL 1

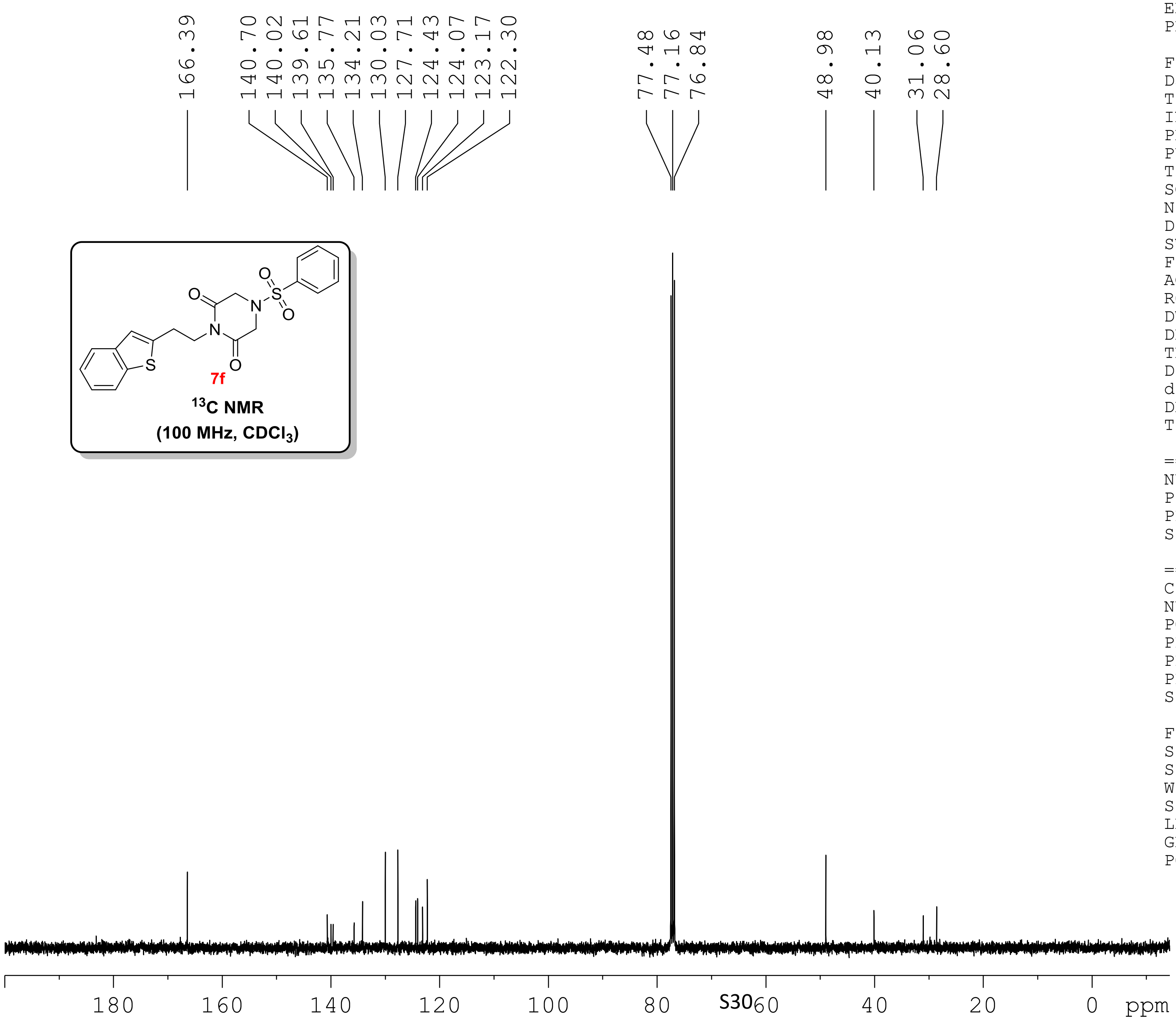

Current Data Parameters
NAME RS-I-BT-PZQ-IM
EXPNO 2
PROCNO 1

F2 - Acquisition Parameters
Date_ 20150724
Time 11.37
INSTRUM spect
PROBHD 5 mm DUL 13C-1
PULPROG zgpg30
TD 65536
SOLVENT CDCl3
NS 223
DS 4
SWH 24038.461 Hz
FIDRES 0.366798 Hz
AQ 1.3631988 sec
RG 40.3
DW 20.800 usec
DE 6.00 usec
TE 296.9 K
D1 2.00000000 sec
d11 0.03000000 sec
DELTA 1.89999998 sec
TD0 1

======== CHANNEL f1 ========
NUC1 13C
P1 9.15 usec
PL1 0.00 dB
SFO1 100.6228298 MHz

======== CHANNEL f2 ========
CPDPRG2 waltz16
NUC2 1H
PCPD2 90.00 usec
PL12 14.90 dB
PL13 14.90 dB
PL2 -3.00 dB
SFO2 400.1316005 MHz

F2 - Processing parameters
SI 32768
SF 100.6127543 MHz
WDW EM
SSB 0
LB 1.00 Hz
GB 0
PC 1.40

PROTON CDCl3 {D:\CRR} KOPAL 1

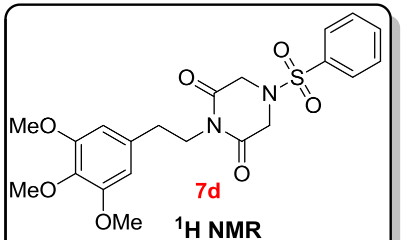

7d

^{1}H NMR

(400 MHz, $CDCl_3$)

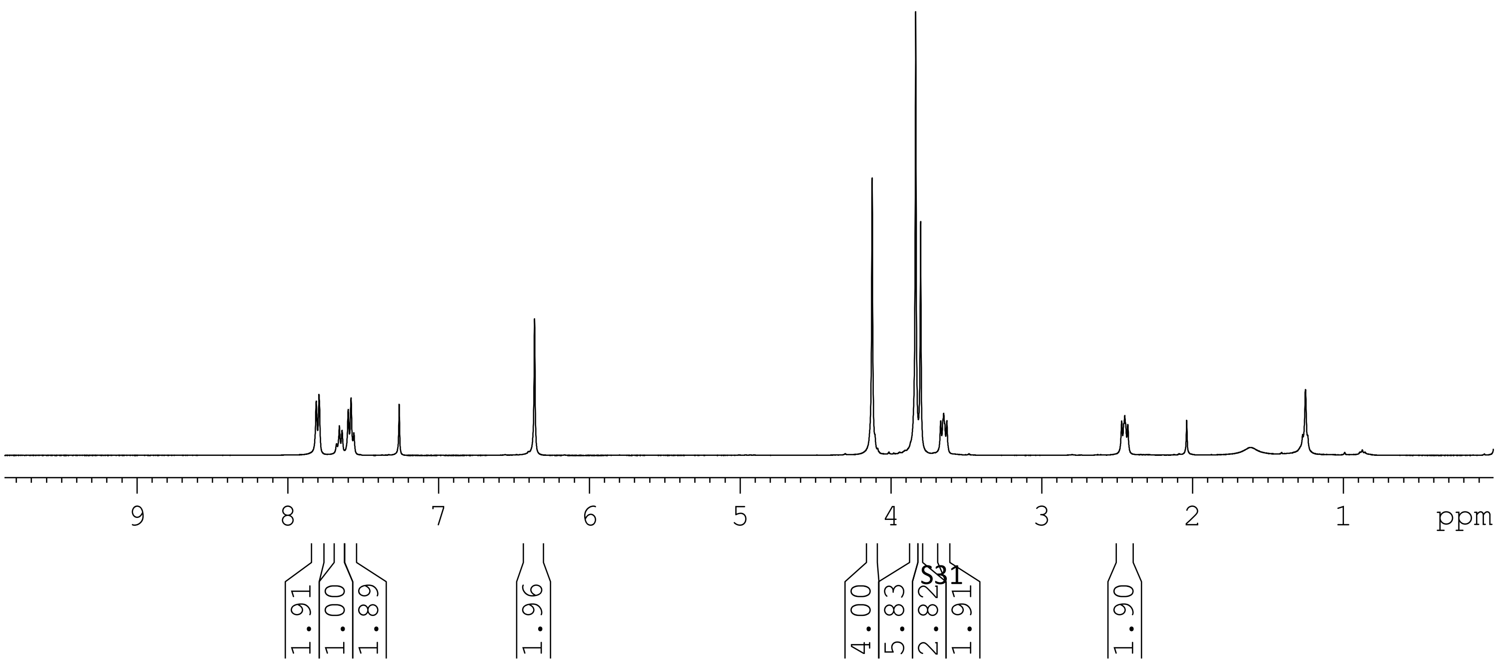

Current Data Parameters
NAME RS-I-3,4,5-PZQ-Im
EXPNO 2
PROCNO 1

F2 - Acquisition Parameters
Date_ 20150203
Time 10.37
INSTRUM spect
PROBHD 5 mm DUL 13C-1
PULPROG zg30
TD 65536
SOLVENT CDCl3
NS 16
DS 2
SWH 8223.685 Hz
FIDRES 0.125483 Hz
AQ 3.9846387 sec
RG 203
DW 60.800 usec
DE 6.00 usec
TE 295.9 K
D1 1.00000000 sec
TD0 1

======== CHANNEL f1 ========
NUC1 1H
P1 11.42 usec
PL1 -3.00 dB
SFO1 400.1324710 MHz

F2 - Processing parameters
SI 32768
SF 400.1300050 MHz
WDW EM
SSB 0
LB 0.30 Hz
GB 0
PC 1.00

C13CPD CDCl3 {D:\CRR} KOPAL 1

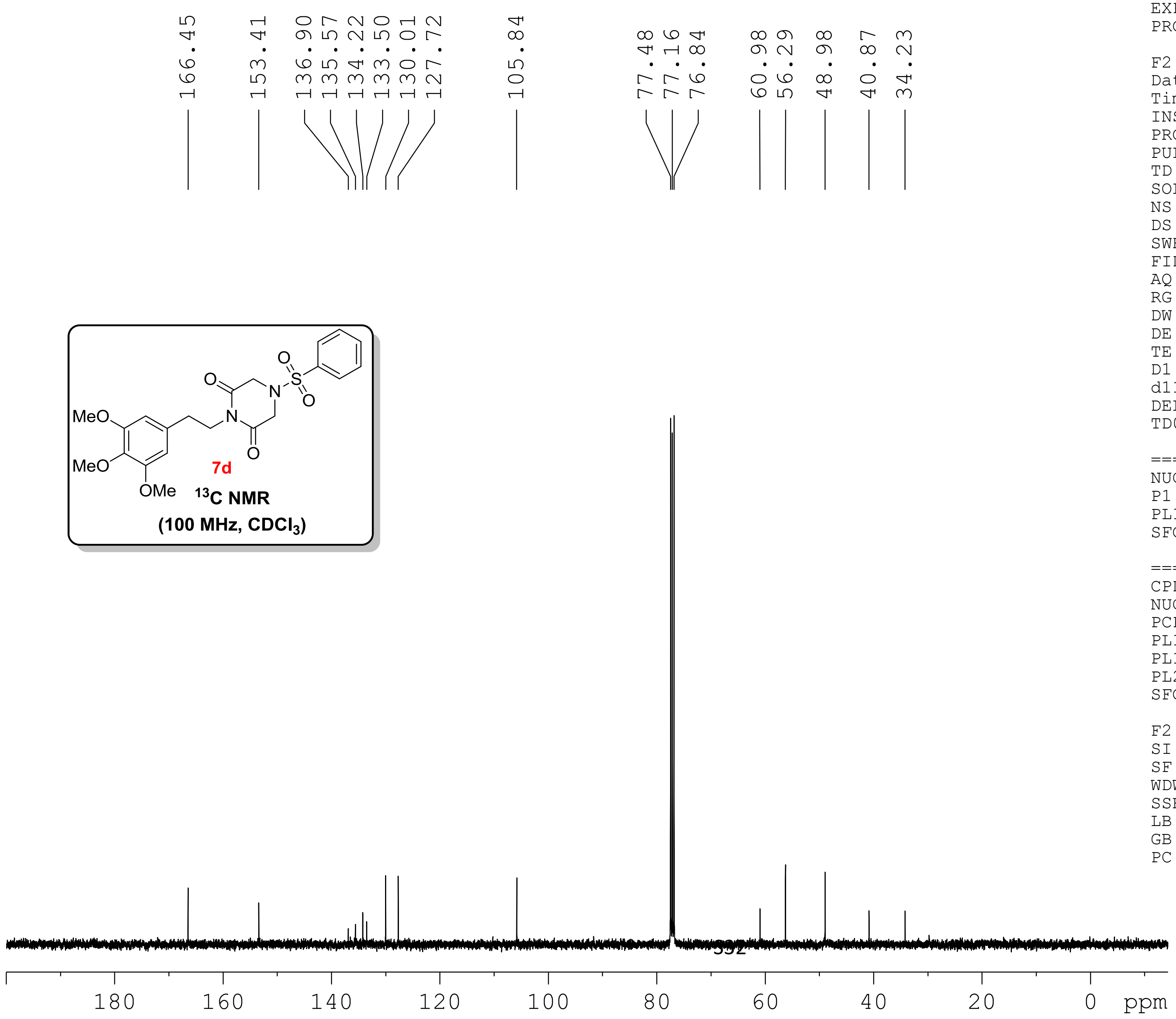

Current Data Parameters
NAME RS-I-3,4,5-PZQ-Im
EXPNO 3
PROCNO 1

F2 - Acquisition Parameters
Date_ 20150203
Time 10.44
INSTRUM spect
PROBHD 5 mm DUL 13C-1
PULPROG zgpg30
TD 65536
SOLVENT CDCl3
NS 229
DS 4
SWH 24038.461 Hz
FIDRES 0.366798 Hz
AQ 1.3631988 sec
RG 32
DW 20.800 usec
DE 6.00 usec
TE 296.7 K
D1 2.00000000 sec
d11 0.03000000 sec
DELTA 1.89999998 sec
TD0 1

======== CHANNEL f1 ========
NUC1 13C
P1 9.15 usec
PL1 0.00 dB
SFO1 100.6228298 MHz

======== CHANNEL f2 ========
CPDPRG2 waltz16
NUC2 1H
PCPD2 90.00 usec
PL12 14.90 dB
PL13 14.90 dB
PL2 -3.00 dB
SFO2 400.1316005 MHz

F2 - Processing parameters
SI 32768
SF 100.6127543 MHz
WDW EM
SSB 0
LB 1.00 Hz
GB 0
PC 1.40

PROTON CDCl3 {D:\CRR} KOPAL 1

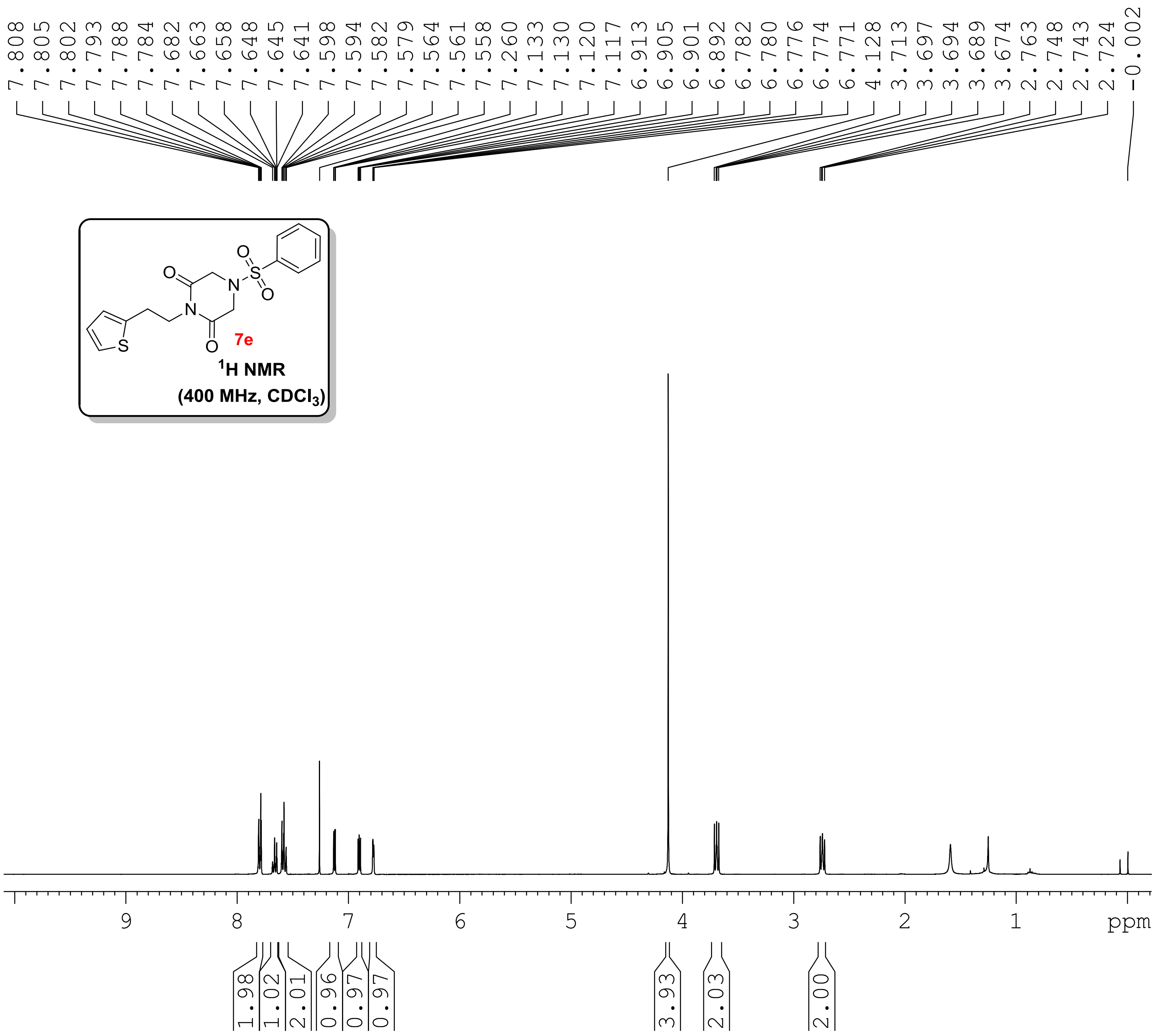

Current Data Parameters
NAME RS-I-THIO-PZQ-IM
EXPNO 1
PROCNO 1

F2 - Acquisition Parameters
Date_ 20150225
Time 16.05
INSTRUM spect
PROBHD 5 mm DUL 13C-1
PULPROG zg30
TD 65536
SOLVENT CDCl3
NS 16
DS 2
SWH 8223.685 Hz
FIDRES 0.125483 Hz
AQ 3.9846387 sec
RG 203
DW 60.800 usec
DE 6.00 usec
TE 295.7 K
D1 1.00000000 sec
TD0 1

======== CHANNEL f1 ========
NUC1 1H
P1 11.42 usec
PL1 -3.00 dB
SFO1 400.1324710 MHz

F2 - Processing parameters
SI 32768
SF 400.1300051 MHz
WDW EM
SSB 0
LB 0.30 Hz
GB 0
PC 1.00

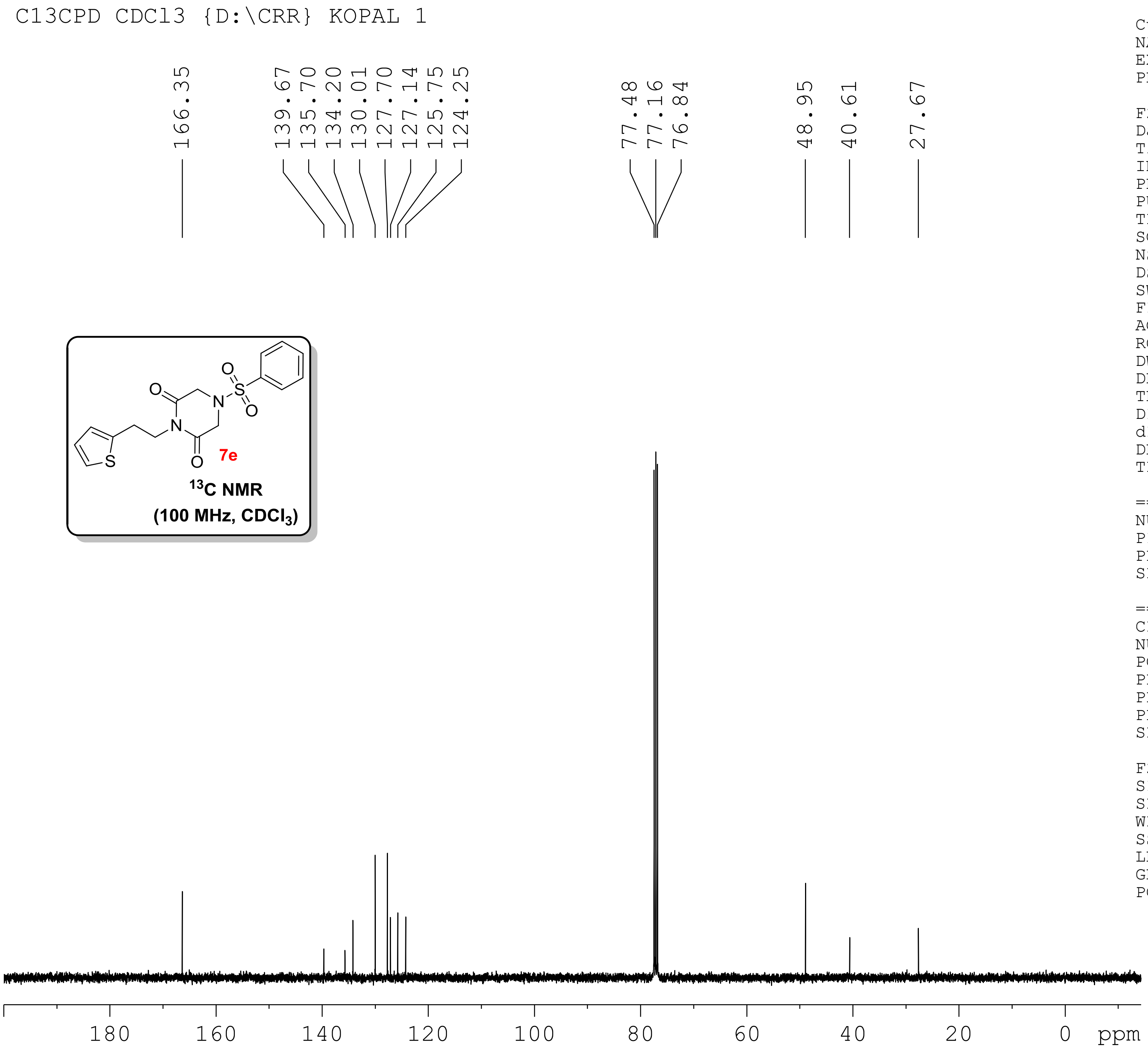


```
Current Data Parameters
NAME        RS-I-THIO-PZQ-IM
EXPNO                      2
PROCNO                     1

F2 - Acquisition Parameters
Date_               20150226
Time                   12.22
INSTRUM                spect
PROBHD    5 mm DUL 13C-1
PULPROG              zgpg30
TD                     65536
SOLVENT                CDCl3
NS                       162
DS                         4
SWH              24038.461 Hz
FIDRES            0.366798 Hz
AQ               1.3631988 sec
RG                      28.5
DW                    20.800 usec
DE                      6.00 usec
TE                     295.1 K
D1                2.00000000 sec
d11               0.03000000 sec
DELTA             1.89999998 sec
TD0                        1

======== CHANNEL f1 ========
NUC1                     13C
P1                      9.15 usec
PL1                     0.00 dB
SFO1             100.6228298 MHz

======== CHANNEL f2 ========
CPDPRG2              waltz16
NUC2                      1H
PCPD2                  90.00 usec
PL12                   14.90 dB
PL13                   14.90 dB
PL2                    -3.00 dB
SFO2             400.1316005 MHz

F2 - Processing parameters
SI                     32768
SF               100.6127553 MHz
WDW                       EM
SSB                        0
LB                      1.00 Hz
GB                         0
PC                      1.40
```

PROTON CDCl3 {D:\CRR} KOPAL 1

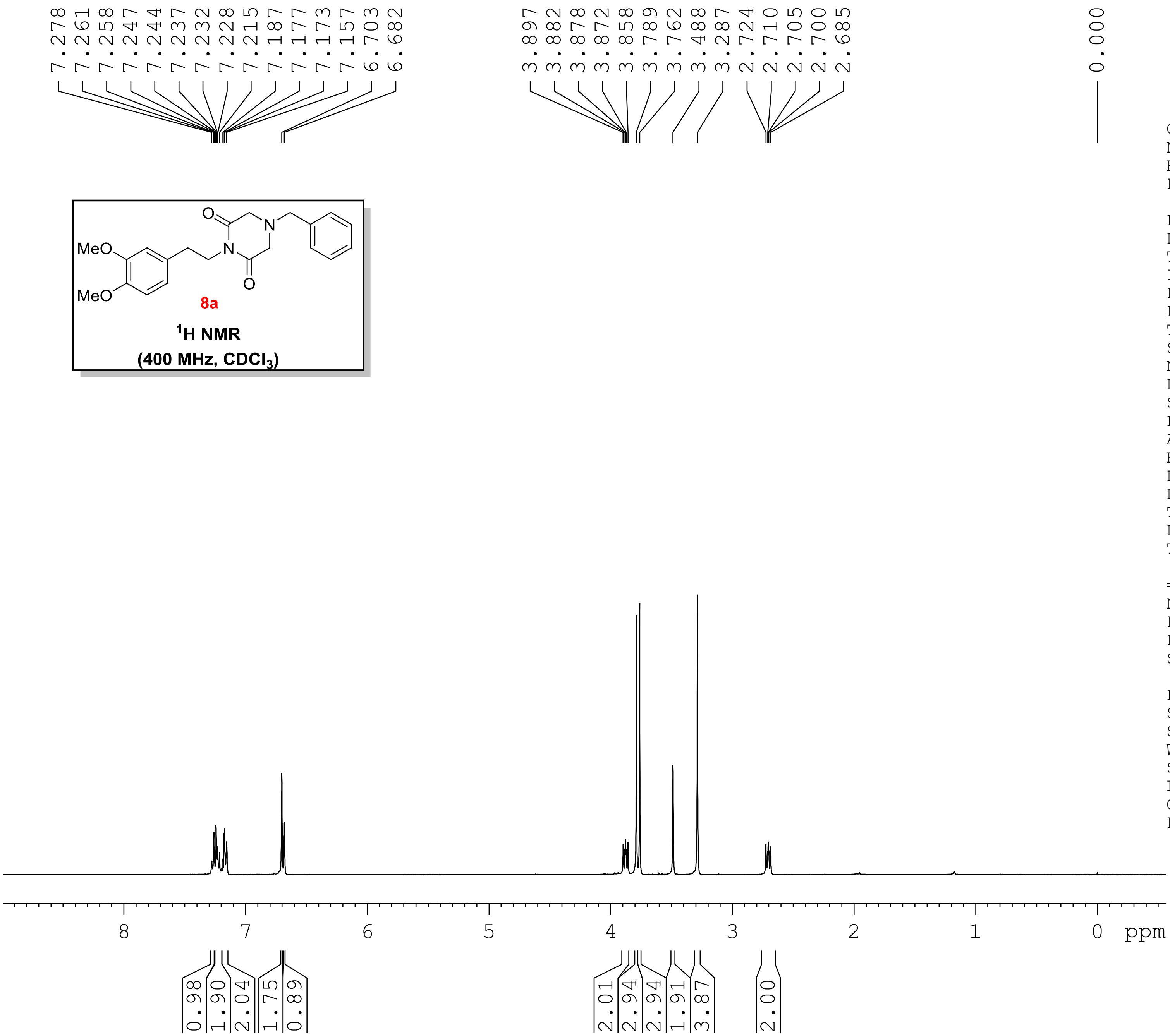

Current Data Parameters
NAME RS-I-Bn-3,4-PZQ-Im
EXPNO 3
PROCNO 1

F2 - Acquisition Parameters
Date_ 20150831
Time 11.39
INSTRUM spect
PROBHD 5 mm DUL 13C-1
PULPROG zg30
TD 65536
SOLVENT CDCl3
NS 16
DS 2
SWH 8223.685 Hz
FIDRES 0.125483 Hz
AQ 3.9846387 sec
RG 71.8
DW 60.800 usec
DE 6.00 usec
TE 295.7 K
D1 1.00000000 sec
TD0 1

======== CHANNEL f1 ========
NUC1 1H
P1 11.42 usec
PL1 -3.00 dB
SFO1 400.1324710 MHz

F2 - Processing parameters
SI 32768
SF 400.1300342 MHz
WDW EM
SSB 0
LB 0.30 Hz
GB 0
PC 1.00

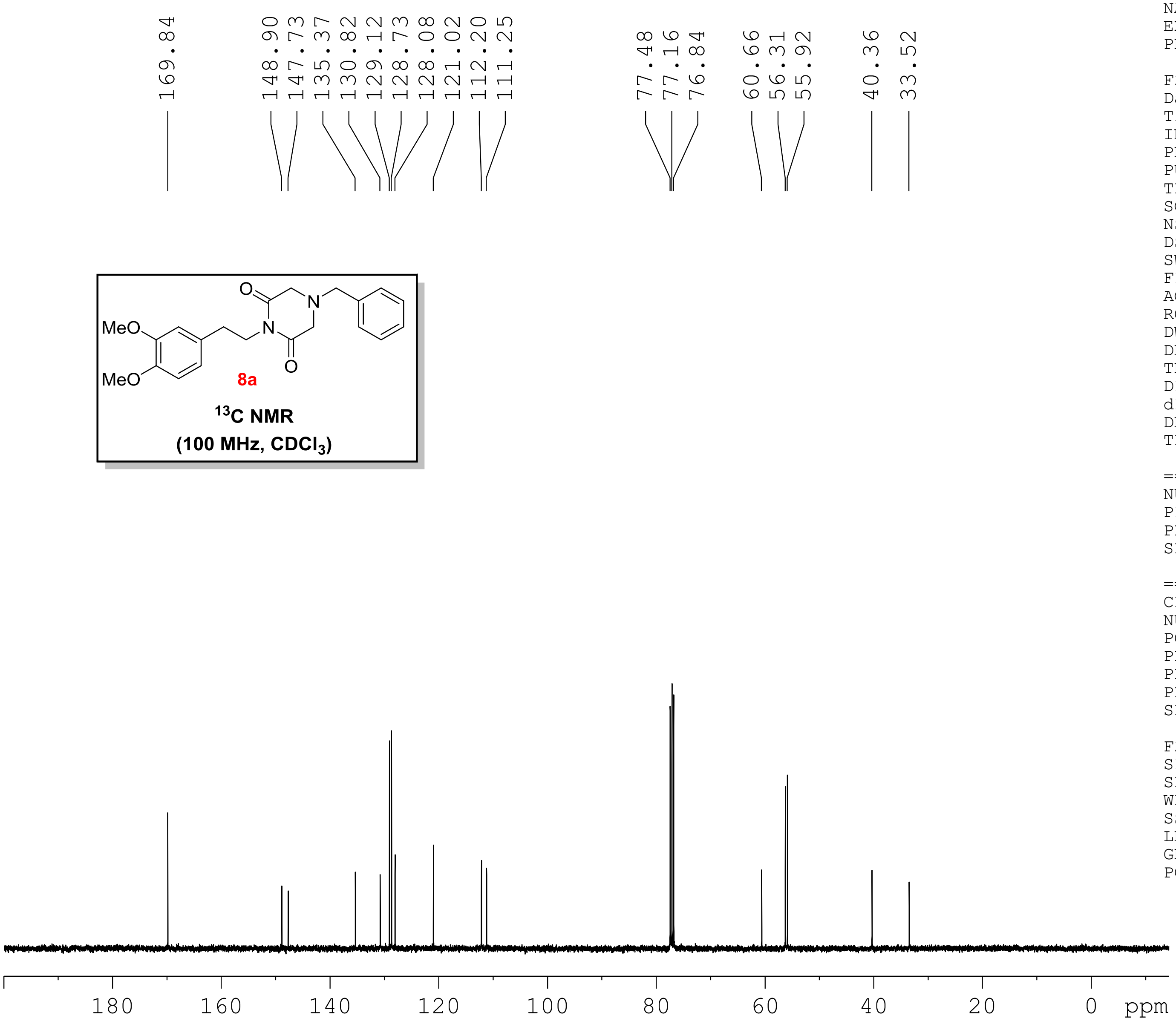


```
Current Data Parameters
NAME      RS-I-Bn-3,4-PZQ-Im
EXPNO                      2
PROCNO                     1

F2 - Acquisition Parameters
Date_               20150831
Time                   11.34
INSTRUM                spect
PROBHD    5 mm DUL 13C-1
PULPROG              zgpg30
TD                     65536
SOLVENT                CDCl3
NS                        70
DS                         4
SWH              24038.461 Hz
FIDRES            0.366798 Hz
AQ               1.3631988 sec
RG                      45.2
DW                    20.800 usec
DE                      6.00 usec
TE                     296.2 K
D1              2.00000000 sec
d11             0.03000000 sec
DELTA           1.89999998 sec
TD0                        1

======== CHANNEL f1 ========
NUC1                     13C
P1                      9.15 usec
PL1                     0.00 dB
SFO1             100.6228298 MHz

======== CHANNEL f2 ========
CPDPRG2              waltz16
NUC2                      1H
PCPD2                  90.00 usec
PL12                   14.90 dB
PL13                   14.90 dB
PL2                    -3.00 dB
SFO2             400.1316005 MHz

F2 - Processing parameters
SI                     32768
SF               100.6122859 MHz
WDW                       EM
SSB                        0
LB                      1.00 Hz
GB                         0
PC                      1.40
```

PROTON CDCl3 {D:\CRR} KOPAL 1

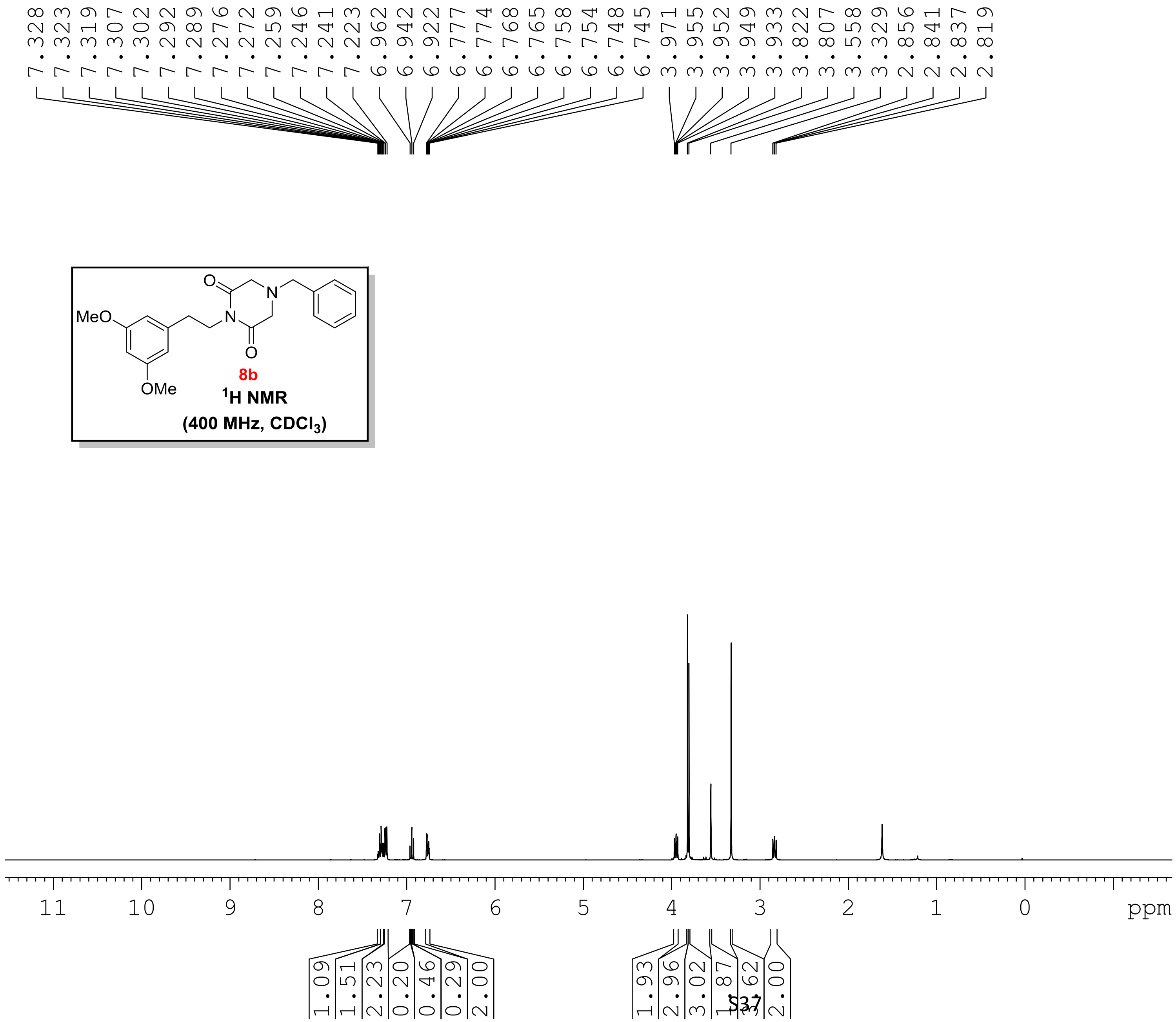

Current Data Parameters
NAME RS-I-3,5-BN-PZQ-IM
EXPNO 1
PROCNO 1

F2 - Acquisition Parameters
Date_ 20151004
Time 17.08
INSTRUM spect
PROBHD 5 mm DUL 13C-1
PULPROG zg30
TD 65536
SOLVENT CDCl3
NS 16
DS 2
SWH 8223.685 Hz
FIDRES 0.125483 Hz
AQ 3.9846387 sec
RG 161
DW 60.800 usec
DE 6.00 usec
TE 294.1 K
D1 1.00000000 sec
TD0 1

======== CHANNEL f1 ========
NUC1 1H
P1 11.42 usec
PL1 -3.00 dB
SFO1 400.1324710 MHz

F2 - Processing parameters
SI 32768
SF 400.1300198 MHz
WDW EM
SSB 0
LB 0.30 Hz
GB 0
PC 1.00

C13CPD CDCl3 {D:\CRR} KOPAL 1

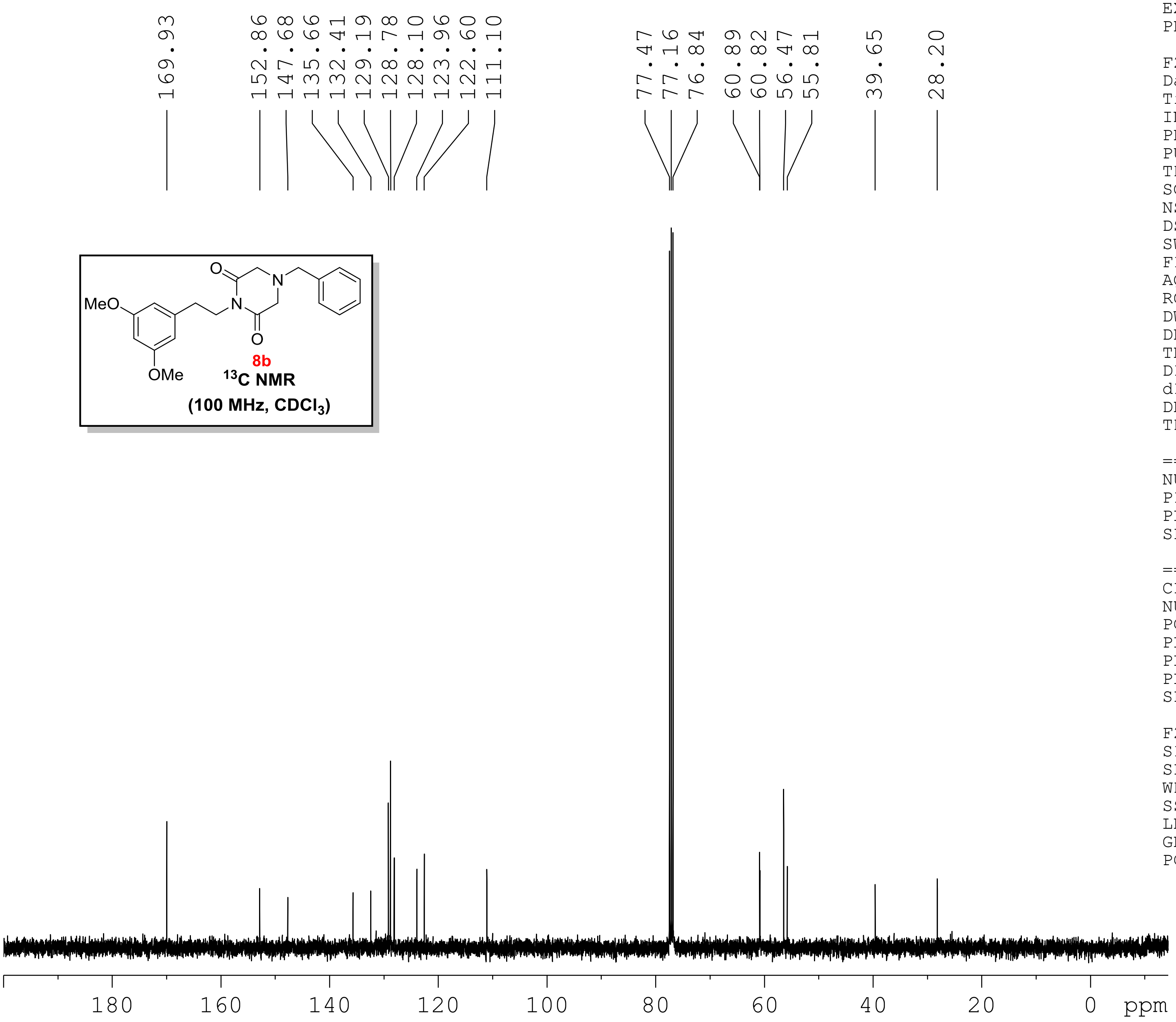

Current Data Parameters
NAME RS-I-3,5-BN-PZQ-IM
EXPNO 2
PROCNO 1

F2 - Acquisition Parameters
Date_ 20151004
Time 17.28
INSTRUM spect
PROBHD 5 mm DUL 13C-1
PULPROG zgpg30
TD 65536
SOLVENT CDCl3
NS 83
DS 4
SWH 24038.461 Hz
FIDRES 0.366798 Hz
AQ 1.3631988 sec
RG 32
DW 20.800 usec
DE 6.00 usec
TE 294.4 K
D1 2.00000000 sec
d11 0.03000000 sec
DELTA 1.89999998 sec
TD0 1

======== CHANNEL f1 ========
NUC1 13C
P1 9.15 usec
PL1 0.00 dB
SFO1 100.6228298 MHz

======== CHANNEL f2 ========
CPDPRG2 waltz16
NUC2 1H
PCPD2 90.00 usec
PL12 14.90 dB
PL13 14.90 dB
PL2 -3.00 dB
SFO2 400.1316005 MHz

F2 - Processing parameters
SI 32768
SF 100.6127562 MHz
WDW EM
SSB 0
LB 1.00 Hz
GB 0
PC 1.40

PROTON CDCl3 {D:\CRR} KOPAL 1

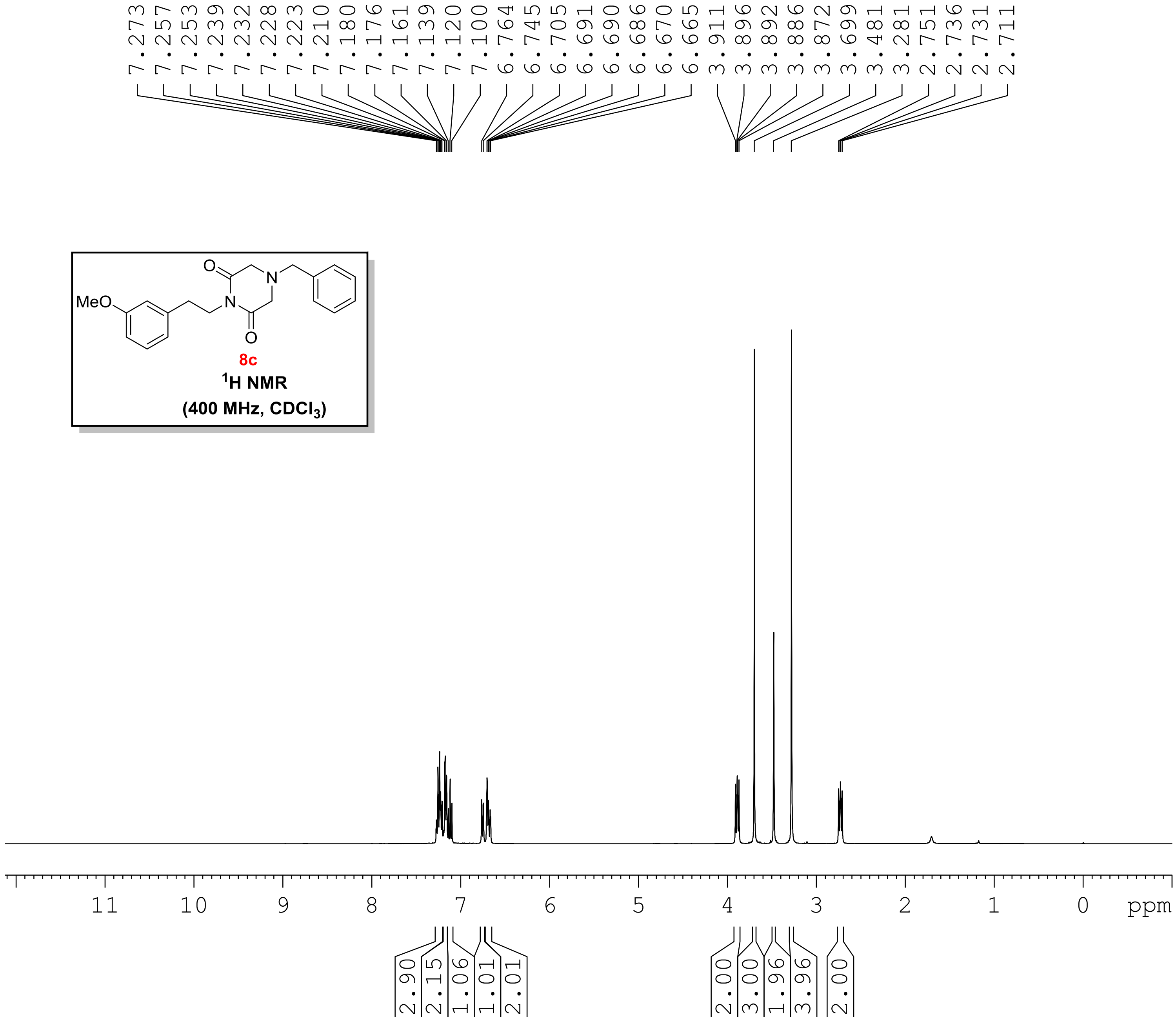


```
Current Data Parameters
NAME      RS-I-3-BN-PZQ-IM
EXPNO                    1
PROCNO                   1

F2 - Acquisition Parameters
Date_             20150924
Time                 14.09
INSTRUM              spect
PROBHD    5 mm DUL 13C-1
PULPROG               zg30
TD                   65536
SOLVENT              CDCl3
NS                      16
DS                       2
SWH               8223.685 Hz
FIDRES            0.125483 Hz
AQ               3.9846387 sec
RG                      64
DW                  60.800 usec
DE                    6.00 usec
TE                   294.5 K
D1              1.00000000 sec
TD0                      1

======== CHANNEL f1 ========
NUC1                    1H
P1                   11.42 usec
PL1                  -3.00 dB
SFO1           400.1324710 MHz

F2 - Processing parameters
SI                   32768
SF             400.1300441 MHz
WDW                     EM
SSB                      0
LB                    0.30 Hz
GB                       0
PC                    1.00
```

C13CPD CDCl3 {D:\CRR} KOPAL 1

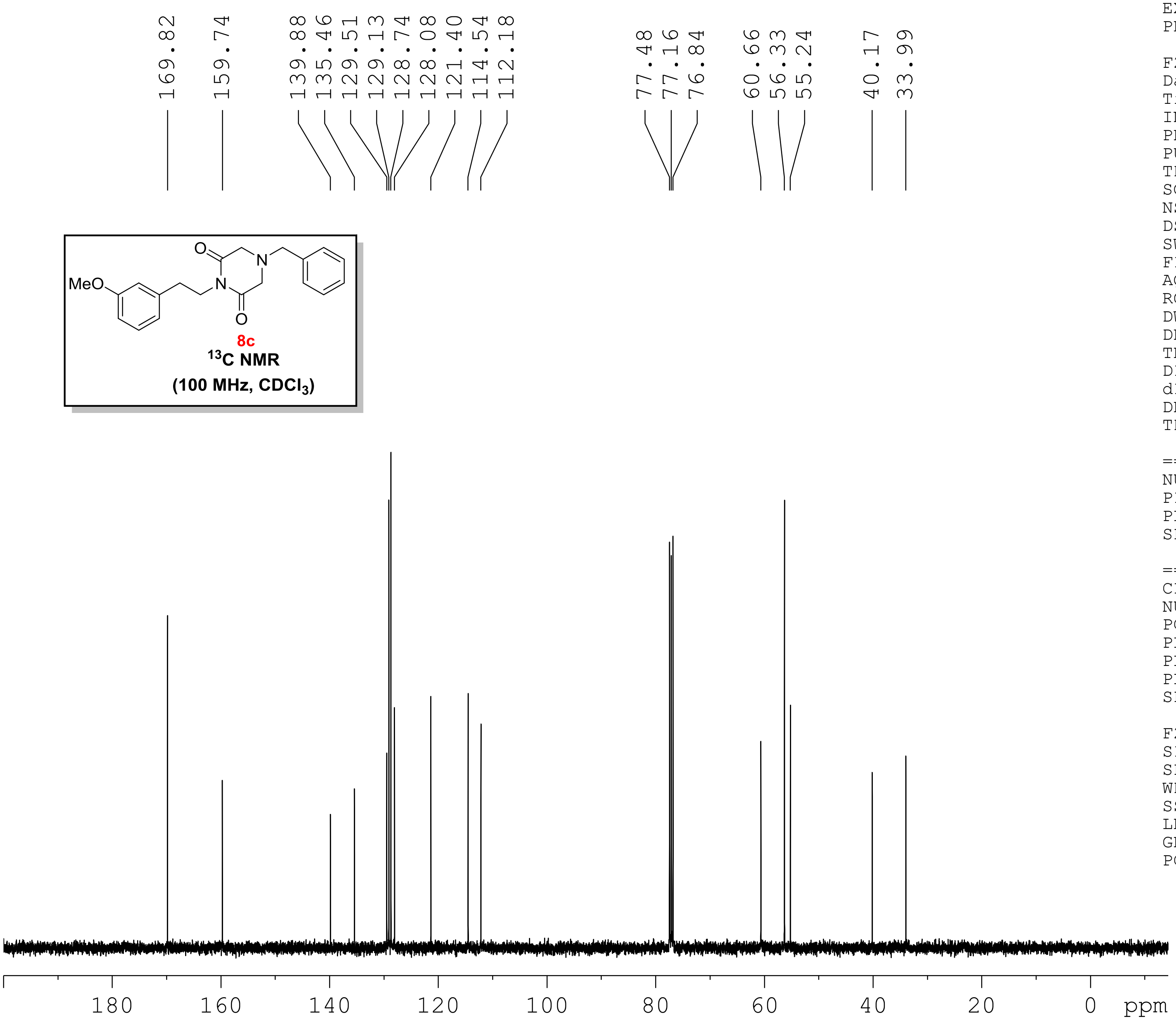

8c

^{13}C NMR

(100 MHz, $CDCl_3$)

Current Data Parameters
NAME RS-I-3-BN-PZQ-IM
EXPNO 2
PROCNO 1

F2 - Acquisition Parameters
Date_ 20150924
Time 14.12
INSTRUM spect
PROBHD 5 mm DUL 13C-1
PULPROG zgpg30
TD 65536
SOLVENT CDCl3
NS 46
DS 4
SWH 24038.461 Hz
FIDRES 0.366798 Hz
AQ 1.3631988 sec
RG 64
DW 20.800 usec
DE 6.00 usec
TE 295.0 K
D1 2.00000000 sec
d11 0.03000000 sec
DELTA 1.89999998 sec
TD0 1

======== CHANNEL f1 ========
NUC1 13C
P1 9.15 usec
PL1 0.00 dB
SFO1 100.6228298 MHz

======== CHANNEL f2 ========
CPDPRG2 waltz16
NUC2 1H
PCPD2 90.00 usec
PL12 14.90 dB
PL13 14.90 dB
PL2 -3.00 dB
SFO2 400.1316005 MHz

F2 - Processing parameters
SI 32768
SF 100.6127646 MHz
WDW EM
SSB 0
LB 1.00 Hz
GB 0
PC 1.40

PROTON CDCl3 {D:\CRR} KOPAL 1

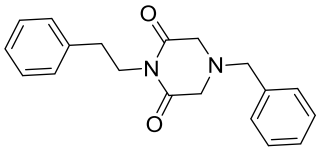

8h

^{1}H NMR

(400 MHz, $CDCl_3$)

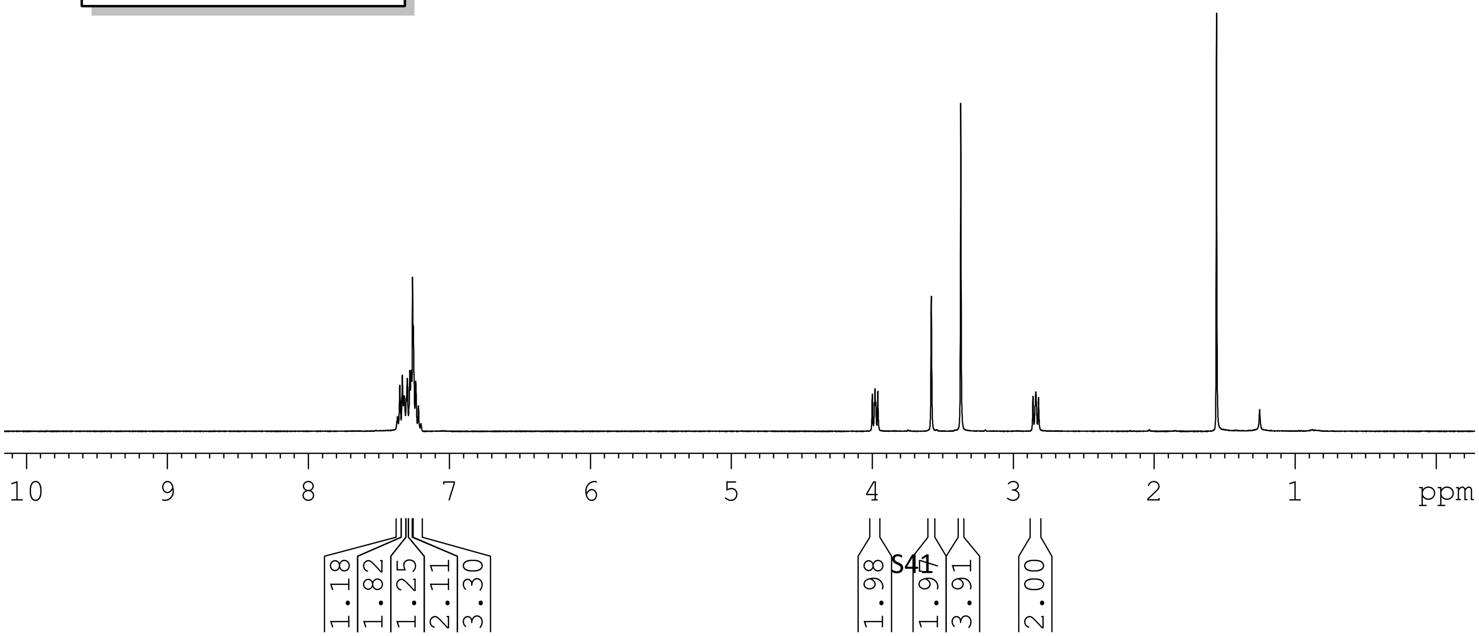


```
Current Data Parameters
NAME     RS-I-Bn-UN-SUB -PZQ-IM
EXPNO                    1
PROCNO                   1

F2 - Acquisition Parameters
Date_             20150825
Time                 11.19
INSTRUM              spect
PROBHD   5 mm DUL 13C-1
PULPROG               zg30
TD                   65536
SOLVENT              CDCl3
NS                      16
DS                       2
SWH               8223.685 Hz
FIDRES            0.125483 Hz
AQ               3.9846387 sec
RG                     256
DW                  60.800 usec
DE                    6.00 usec
TE                   295.4 K
D1              1.00000000 sec
TD0                      1

======== CHANNEL f1 ========
NUC1                    1H
P1                   11.42 usec
PL1                  -3.00 dB
SFO1           400.1324710 MHz

F2 - Processing parameters
SI                   32768
SF             400.1300048 MHz
WDW                     EM
SSB                      0
LB                    0.30 Hz
GB                       0
PC                    1.00
```

C13CPD CDCl3 {D:\CRR} KOPAL 1

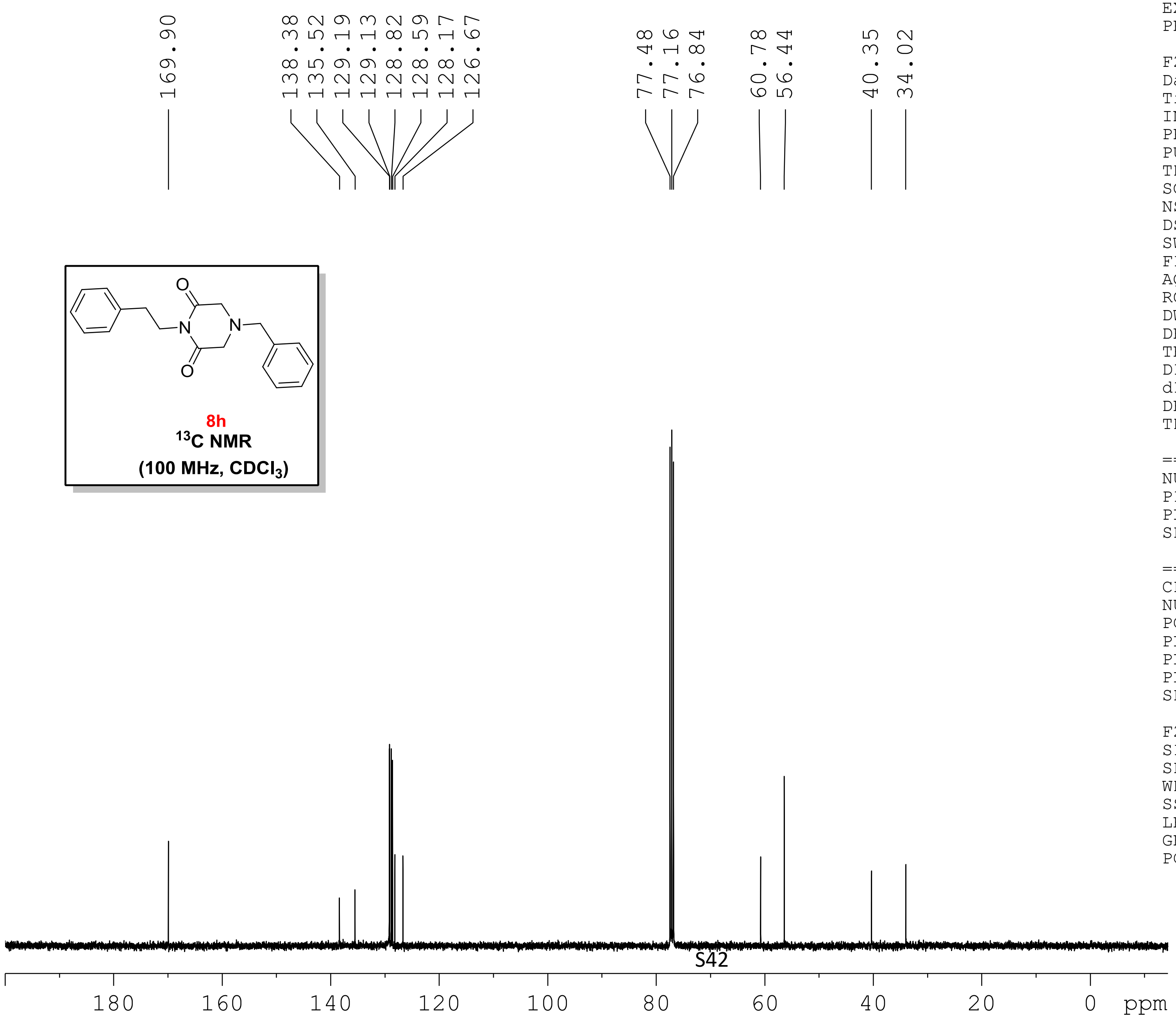

Current Data Parameters
NAME RS-I-Bn-PZQ-Im
EXPNO 1
PROCNO 1

F2 - Acquisition Parameters
Date_ 20150825
Time 12.09
INSTRUM spect
PROBHD 5 mm DUL 13C-1
PULPROG zgpg30
TD 65536
SOLVENT CDCl3
NS 256
DS 4
SWH 24038.461 Hz
FIDRES 0.366798 Hz
AQ 1.3631988 sec
RG 40.3
DW 20.800 usec
DE 6.00 usec
TE 295.8 K
D1 2.00000000 sec
d11 0.03000000 sec
DELTA 1.89999998 sec
TD0 1

======== CHANNEL f1 ========
NUC1 13C
P1 9.15 usec
PL1 0.00 dB
SFO1 100.6228298 MHz

======== CHANNEL f2 ========
CPDPRG2 waltz16
NUC2 1H
PCPD2 90.00 usec
PL12 14.90 dB
PL13 14.90 dB
PL2 -3.00 dB
SFO2 400.1316005 MHz

F2 - Processing parameters
SI 32768
SF 100.6127561 MHz
WDW EM
SSB 0
LB 1.00 Hz
GB 0
PC 1.40

PROTON CDCl3 {D:\CRR} KOPAL 1

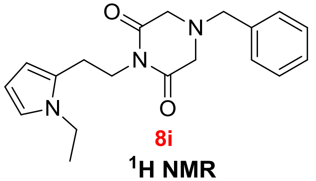

8i

^{1}H NMR

(400 MHz, $CDCl_3$)

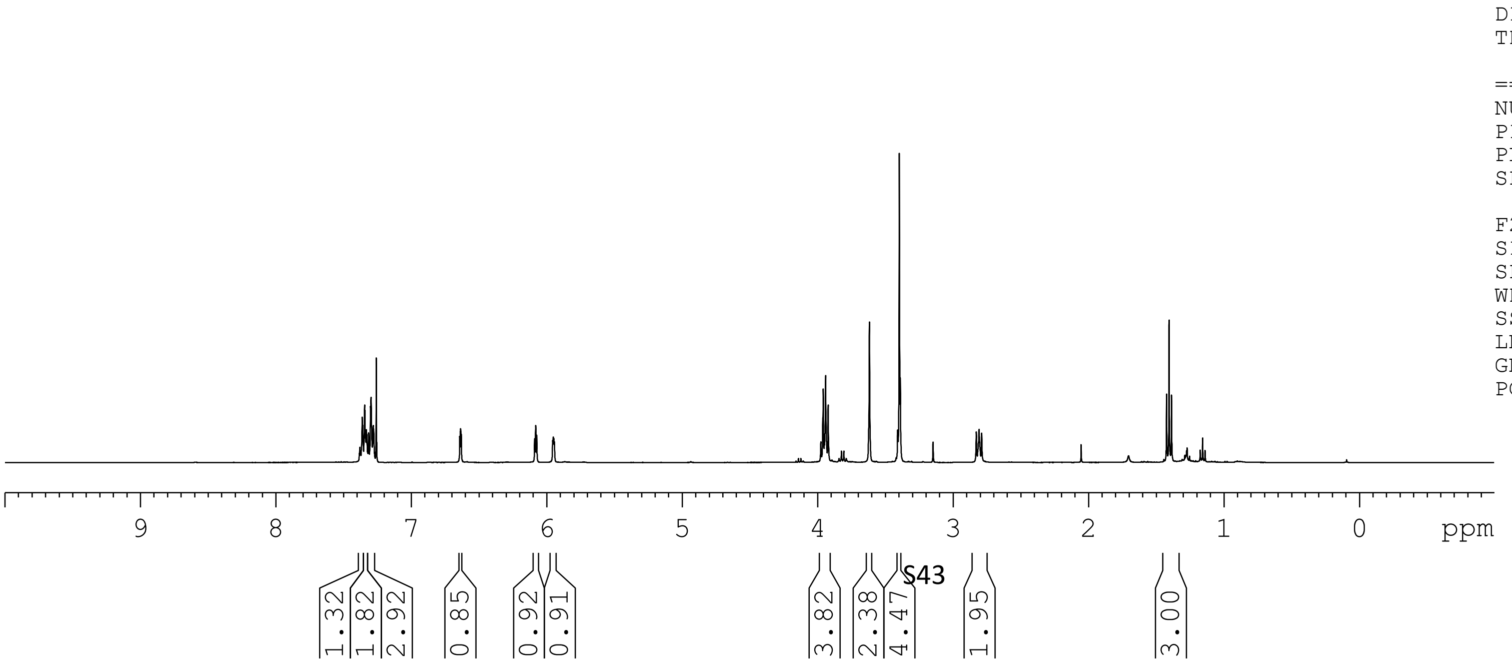

Current Data Parameters
NAME RS-I-BN-NET-PY-PZQ
EXPNO 1
PROCNO 1

F2 - Acquisition Parameters
Date_ 20150831
Time 10.27
INSTRUM spect
PROBHD 5 mm DUL 13C-1
PULPROG zg30
TD 65536
SOLVENT CDCl3
NS 16
DS 2
SWH 8223.685 Hz
FIDRES 0.125483 Hz
AQ 3.9846387 sec
RG 71.8
DW 60.800 usec
DE 6.00 usec
TE 295.1 K
D1 1.00000000 sec
TD0 1

======== CHANNEL f1 ========
NUC1 1H
P1 11.42 usec
PL1 -3.00 dB
SFO1 400.1324710 MHz

F2 - Processing parameters
SI 32768
SF 400.1300050 MHz
WDW EM
SSB 0
LB 0.30 Hz
GB 0
PC 1.00

C13CPD CDCl3 {D:\CRR} KOPAL 1

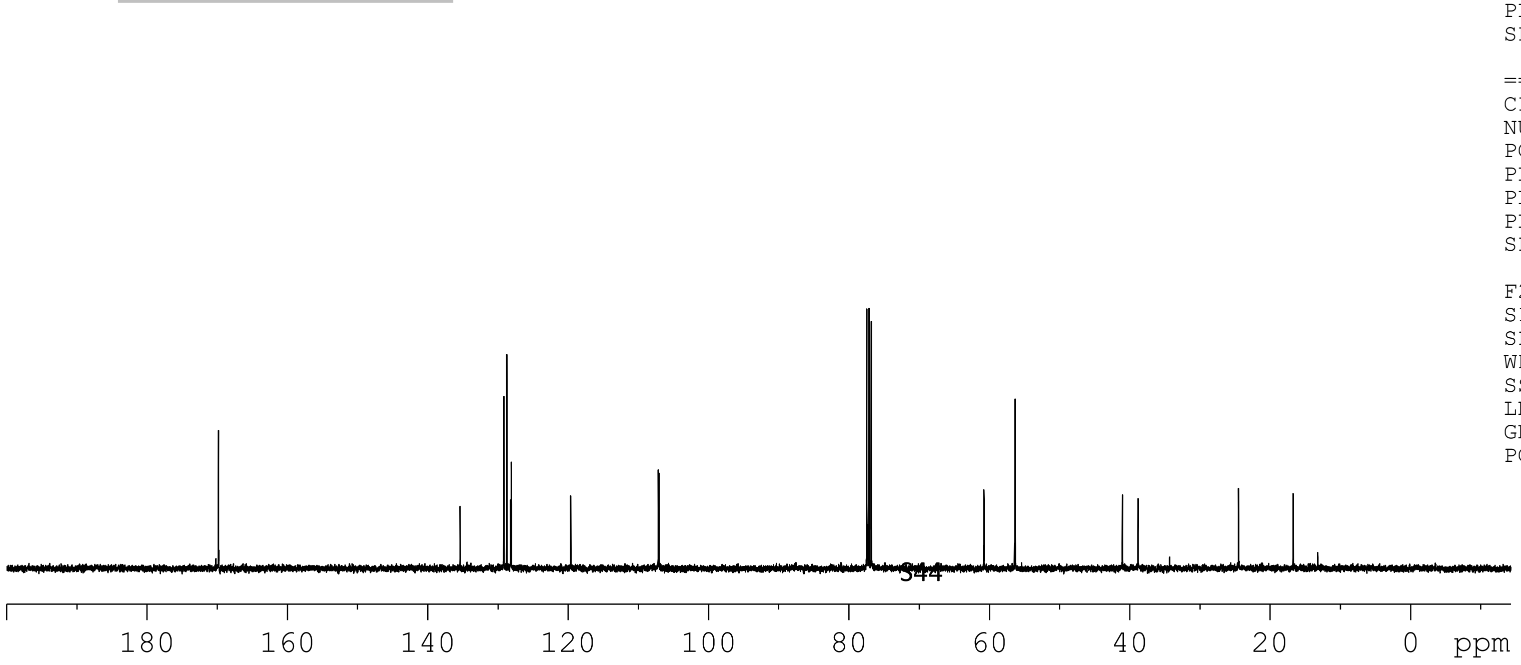

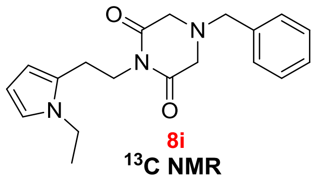

8i

^{13}C NMR

(100 MHz, $CDCl_3$)

```
Current Data Parameters
NAME      RS-I-BN-NET-PY-PZQ
EXPNO                      2
PROCNO                     1

F2 - Acquisition Parameters
Date_               20150831
Time                   10.32
INSTRUM                spect
PROBHD    5 mm DUL 13C-1
PULPROG              zgpg30
TD                     65536
SOLVENT                CDCl3
NS                        80
DS                         4
SWH             24038.461 Hz
FIDRES           0.366798 Hz
AQ              1.3631988 sec
RG                        36
DW                20.800 usec
DE                  6.00 usec
TE                  295.8 K
D1            2.00000000 sec
d11           0.03000000 sec
DELTA         1.89999998 sec
TD0                        1

======== CHANNEL f1 ========
NUC1                     13C
P1                  9.15 usec
PL1                  0.00 dB
SFO1          100.6228298 MHz

======== CHANNEL f2 ========
CPDPRG2              waltz16
NUC2                      1H
PCPD2              90.00 usec
PL12                14.90 dB
PL13                14.90 dB
PL2                 -3.00 dB
SFO2          400.1316005 MHz

F2 - Processing parameters
SI                     32768
SF            100.6127626 MHz
WDW                       EM
SSB                        0
LB                   1.00 Hz
GB                         0
PC                      1.40
```

PROTON CDCl3 {D:\CRR} KOPAL 1

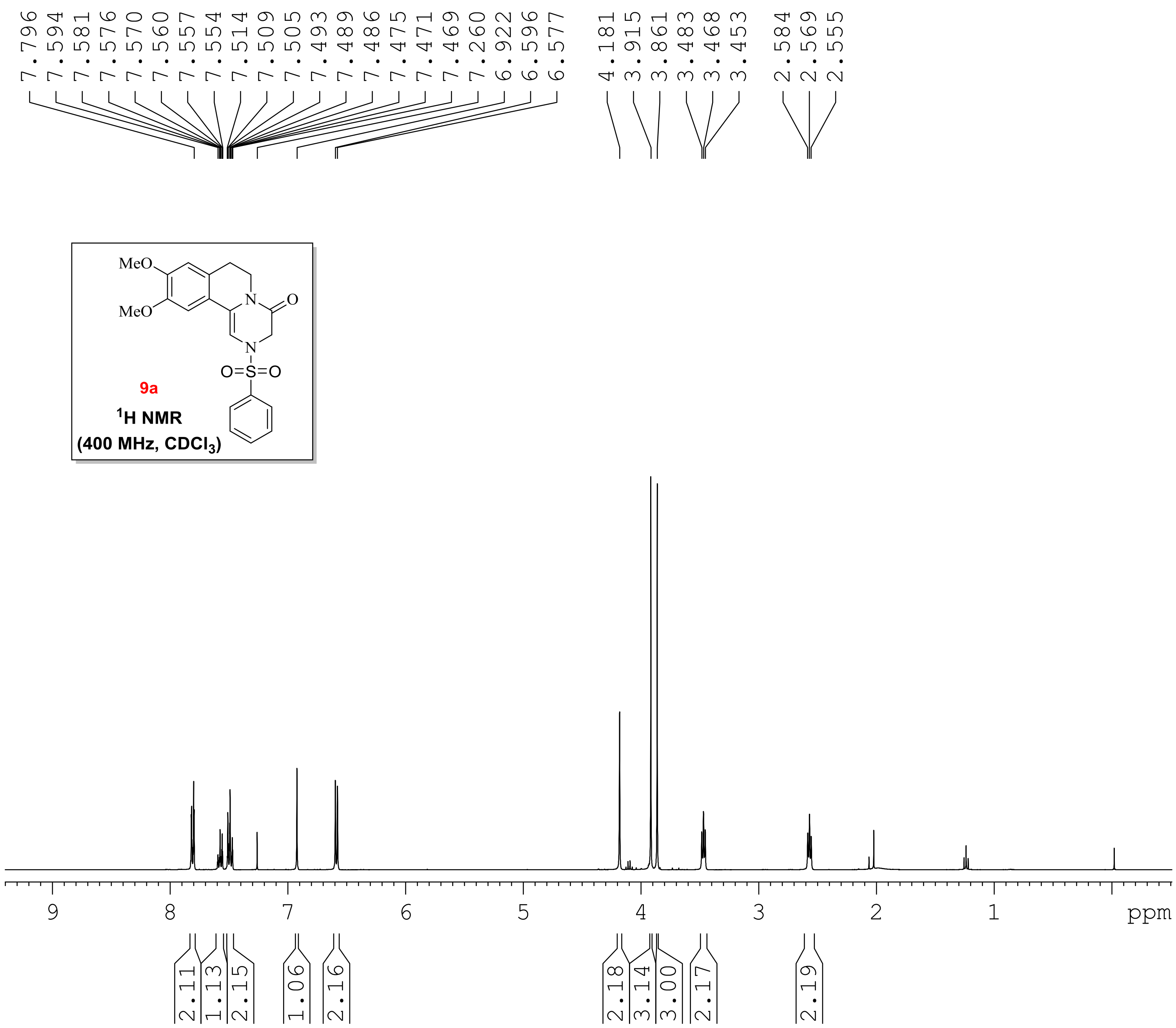


```
Current Data Parameters
NAME       RS-I-PZQ-IM-CY-A
EXPNO                     1
PROCNO                    1

F2 - Acquisition Parameters
Date_              20130711
Time                  14.48
INSTRUM               spect
PROBHD    5 mm BBO BB-1H
PULPROG                zg30
TD                    65536
SOLVENT               CDCl3
NS                       16
DS                        2
SWH              8223.685 Hz
FIDRES           0.125483 Hz
AQ              3.9846387 sec
RG                      181
DW                  60.800 usec
DE                    6.00 usec
TE                   296.8 K
D1             1.00000000 sec
TD0                       1

======== CHANNEL f1 ========
NUC1                     1H
P1                   14.50 usec
PL1                  -0.90 dB
SFO1           400.1324710 MHz

F2 - Processing parameters
SI                    32768
SF             400.1300056 MHz
WDW                      EM
SSB                       0
LB                     0.30 Hz
GB                        0
PC                     1.00
```

C13CPD CDCl3 {D:\CRR} KOPAL 1

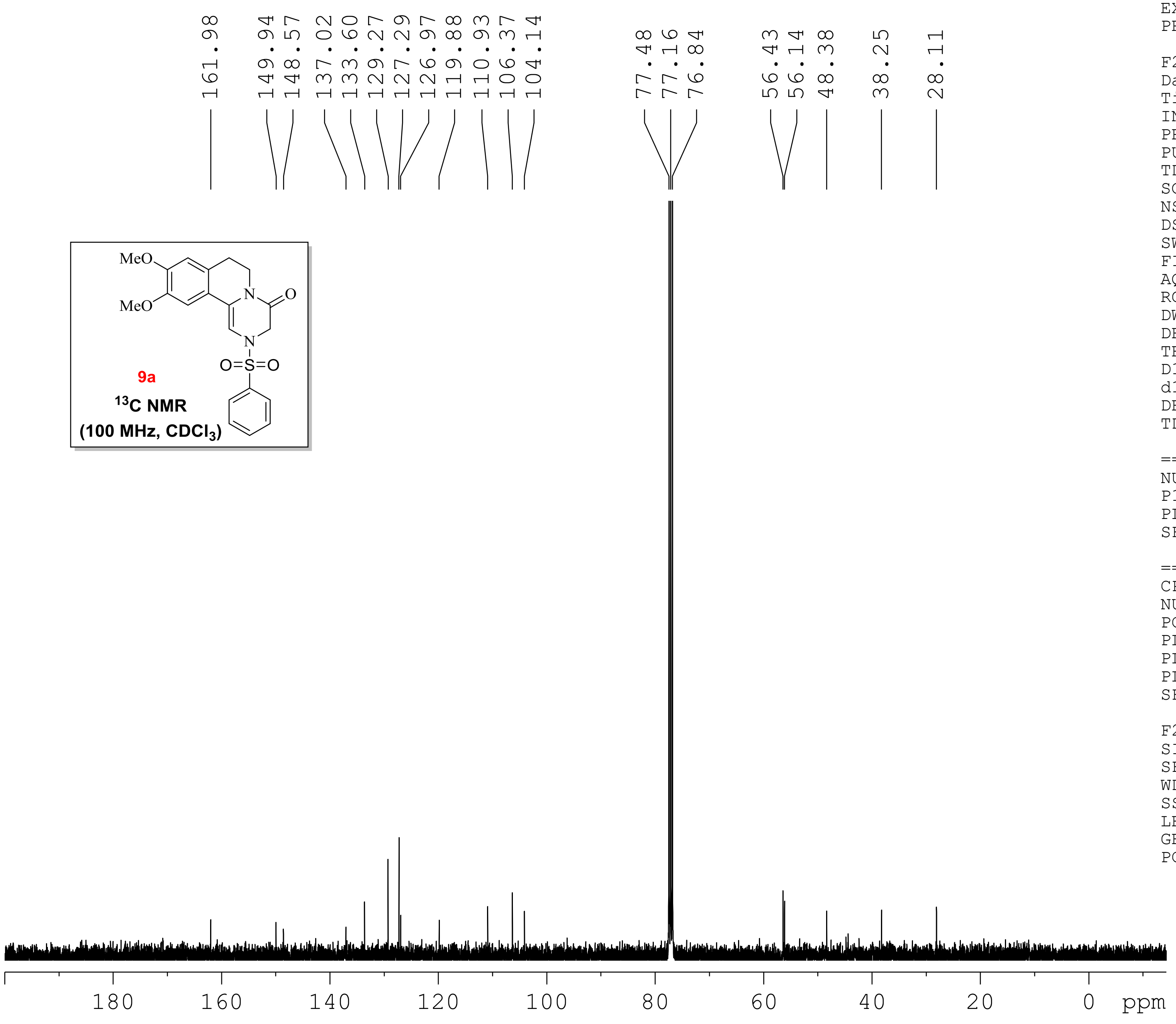


```
Current Data Parameters
NAME        RS-I-3,4-BS-PZQ-XY
EXPNO                2
PROCNO               1

F2 - Acquisition Parameters
Date_          20151008
Time              13.13
INSTRUM           spect
PROBHD   5 mm DUL 13C-1
PULPROG          zgpg30
TD                65536
SOLVENT           CDCl3
NS                  256
DS                    4
SWH           24038.461 Hz
FIDRES         0.366798 Hz
AQ            1.3631988 sec
RG                   57
DW               20.800 usec
DE                 6.00 usec
TE                296.5 K
D1           2.00000000 sec
d11          0.03000000 sec
DELTA        1.89999998 sec
TD0                   1

======== CHANNEL f1 ========
NUC1                13C
P1                 9.15 usec
PL1                0.00 dB
SFO1        100.6228298 MHz

======== CHANNEL f2 ========
CPDPRG2         waltz16
NUC2                 1H
PCPD2             90.00 usec
PL12              14.90 dB
PL13              14.90 dB
PL2               -3.00 dB
SFO2        400.1316005 MHz

F2 - Processing parameters
SI                32768
SF          100.6127535 MHz
WDW                  EM
SSB                   0
LB                 1.00 Hz
GB                    0
PC                 1.40
```

PROTON CDCl3 {D:\CRR} KOPAL 1

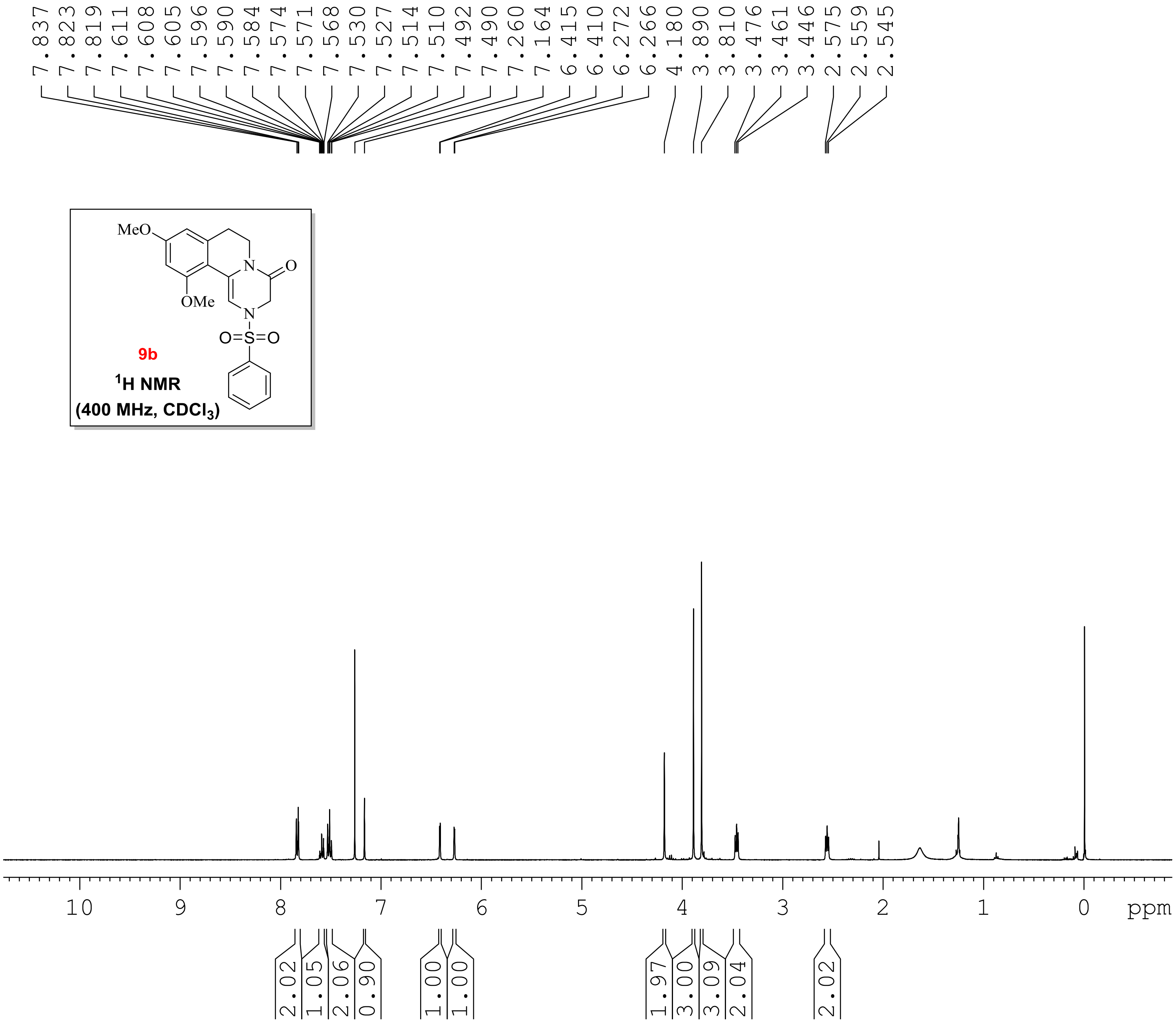

Current Data Parameters
NAME RS-I-3,5-PZQ-CY
EXPNO 1
PROCNO 1

F2 - Acquisition Parameters
Date_ 20130913
Time 15.42
INSTRUM spect
PROBHD 5 mm BBO BB-1H
PULPROG zg30
TD 65536
SOLVENT CDCl3
NS 16
DS 2
SWH 8223.685 Hz
FIDRES 0.125483 Hz
AQ 3.9846387 sec
RG 912
DW 60.800 usec
DE 6.00 usec
TE 293.5 K
D1 1.00000000 sec
TD0 1

======== CHANNEL f1 ========
NUC1 1H
P1 14.50 usec
PL1 -0.90 dB
SFO1 400.1324710 MHz

F2 - Processing parameters
SI 32768
SF 400.1300058 MHz
WDW EM
SSB 0
LB 0.30 Hz
GB 0
PC 1.00

C13CPD CDCl3 {D:\CRR} KOPAL 1

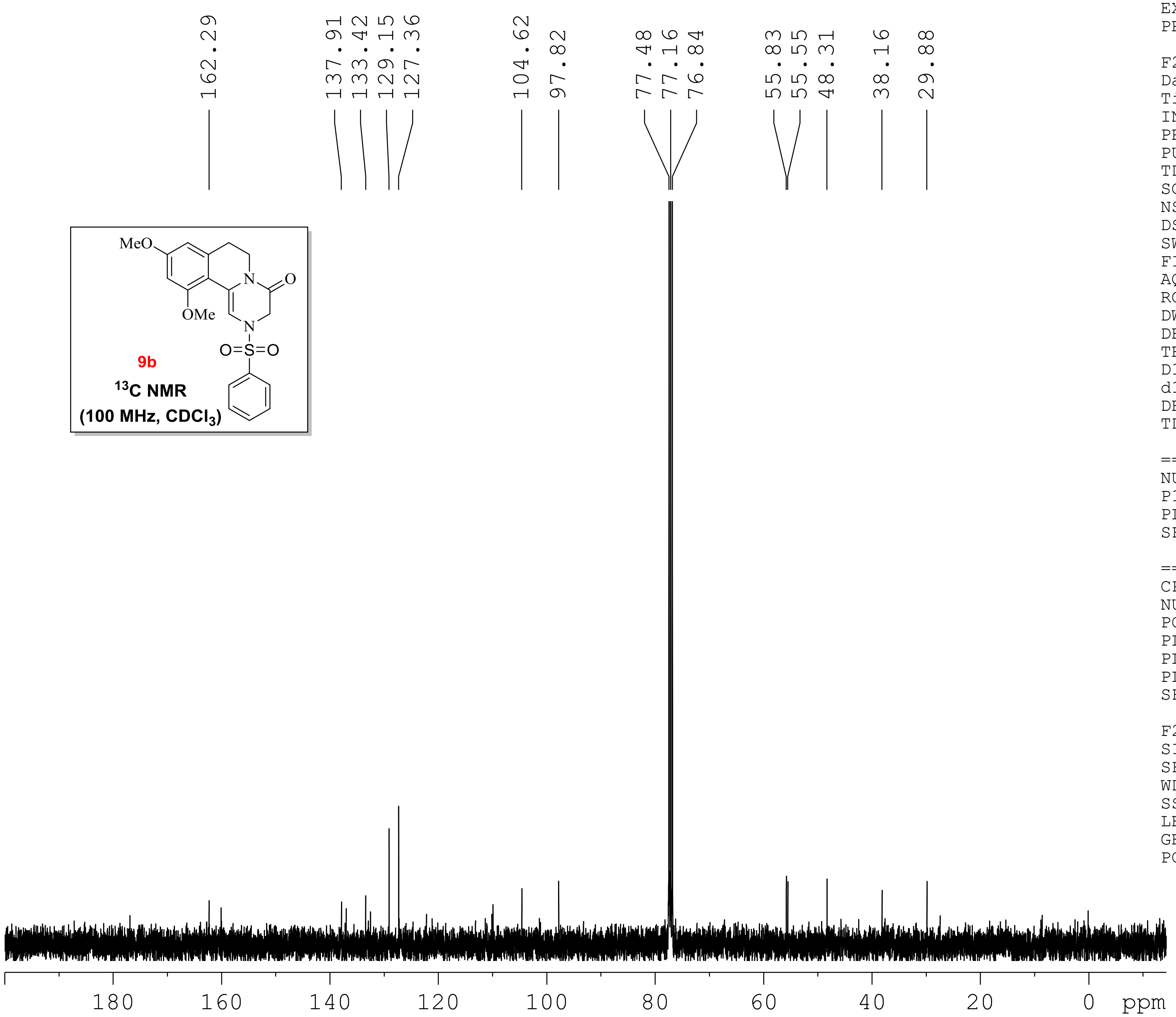

Current Data Parameters

NAME	RS-I-3,5-PZQ-CY	
EXPNO	2	
PROCNO	1	

F2 - Acquisition Parameters

Date_	20130913	
Time	15.47	
INSTRUM	spect	
PROBHD	5 mm BBO BB-1H	
PULPROG	zgpg30	
TD	65536	
SOLVENT	CDCl3	
NS	256	
DS	4	
SWH	24038.461	Hz
FIDRES	0.366798	Hz
AQ	1.3631988	sec
RG	645	
DW	20.800	usec
DE	6.00	usec
TE	294.1	K
D1	2.00000000	sec
d11	0.03000000	sec
DELTA	1.89999998	sec
TD0	1	

======== CHANNEL f1 ========

NUC1	13C	
P1	9.50	usec
PL1	-0.60	dB
SFO1	100.6228298	MHz

======== CHANNEL f2 ========

CPDPRG2	waltz16	
NUC2	1H	
PCPD2	90.00	usec
PL12	14.96	dB
PL13	15.60	dB
PL2	-0.90	dB
SFO2	400.1316005	MHz

F2 - Processing parameters

SI	32768	
SF	100.6127544	MHz
WDW	EM	
SSB	0	
LB	1.00	Hz
GB	0	
PC	1.40	

PROTON CDCl3 {D:\CRR} guest 1

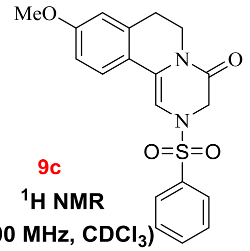

9c

^{1}H NMR

(400 MHz, $CDCl_3$)

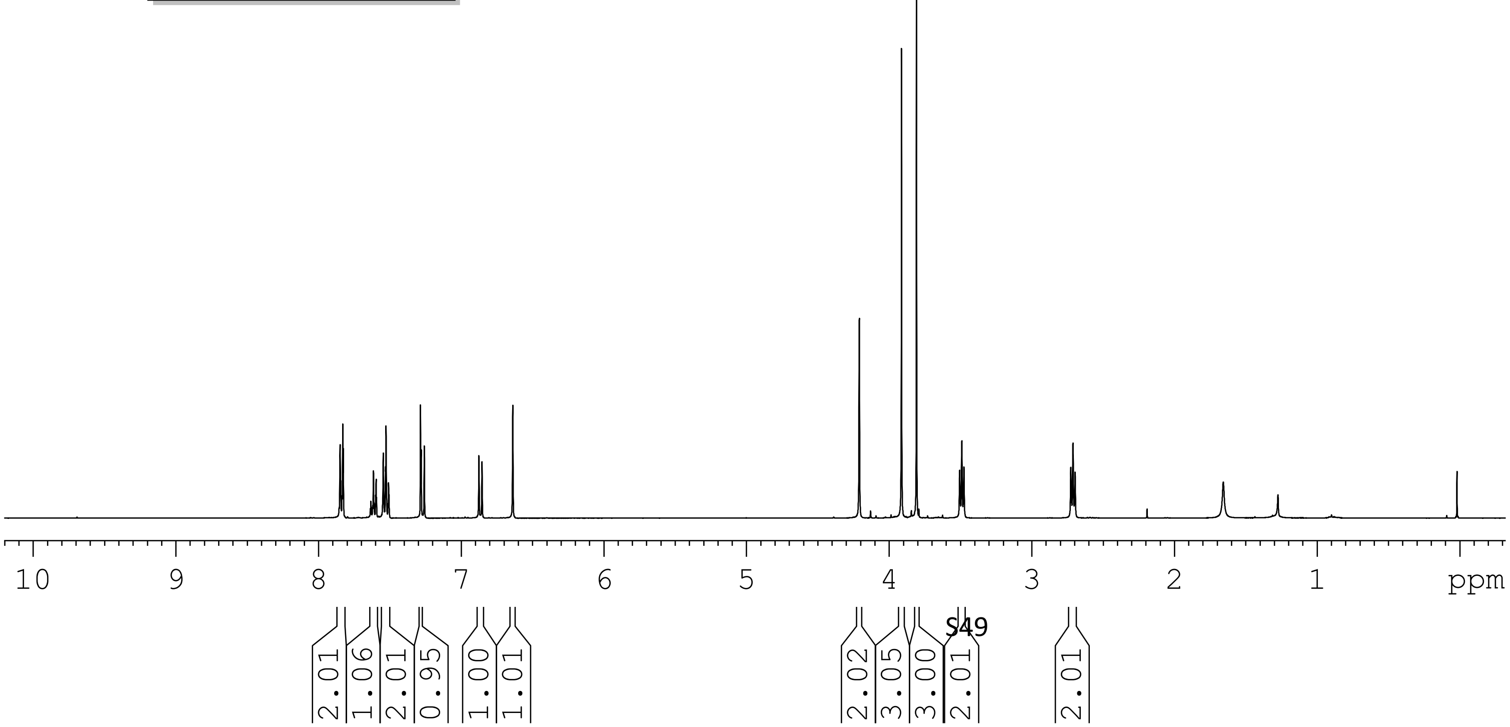


```
Current Data Parameters
NAME       RS-I-3-OMe-PzZQ-CY1
EXPNO                        1
PROCNO                       1

F2 - Acquisition Parameters
Date_                 20150222
Time                     22.55
INSTRUM                  spect
PROBHD      5 mm DUL 13C-1
PULPROG                   zg30
TD                       65536
SOLVENT                  CDCl3
NS                          16
DS                           2
SWH                   8223.685 Hz
FIDRES                0.125483 Hz
AQ                   3.9846387 sec
RG                         181
DW                      60.800 usec
DE                        6.00 usec
TE                       295.1 K
D1                  1.00000000 sec
TD0                          1

======== CHANNEL f1 ========
NUC1                        1H
P1                       11.42 usec
PL1                      -3.00 dB
SFO1               400.1324710 MHz

F2 - Processing parameters
SI                       32768
SF                 400.1299944 MHz
WDW                         EM
SSB                          0
LB                        0.30 Hz
GB                           0
PC                        1.00
```

C13CPD CDCl3 {D:\CRR} guest 1

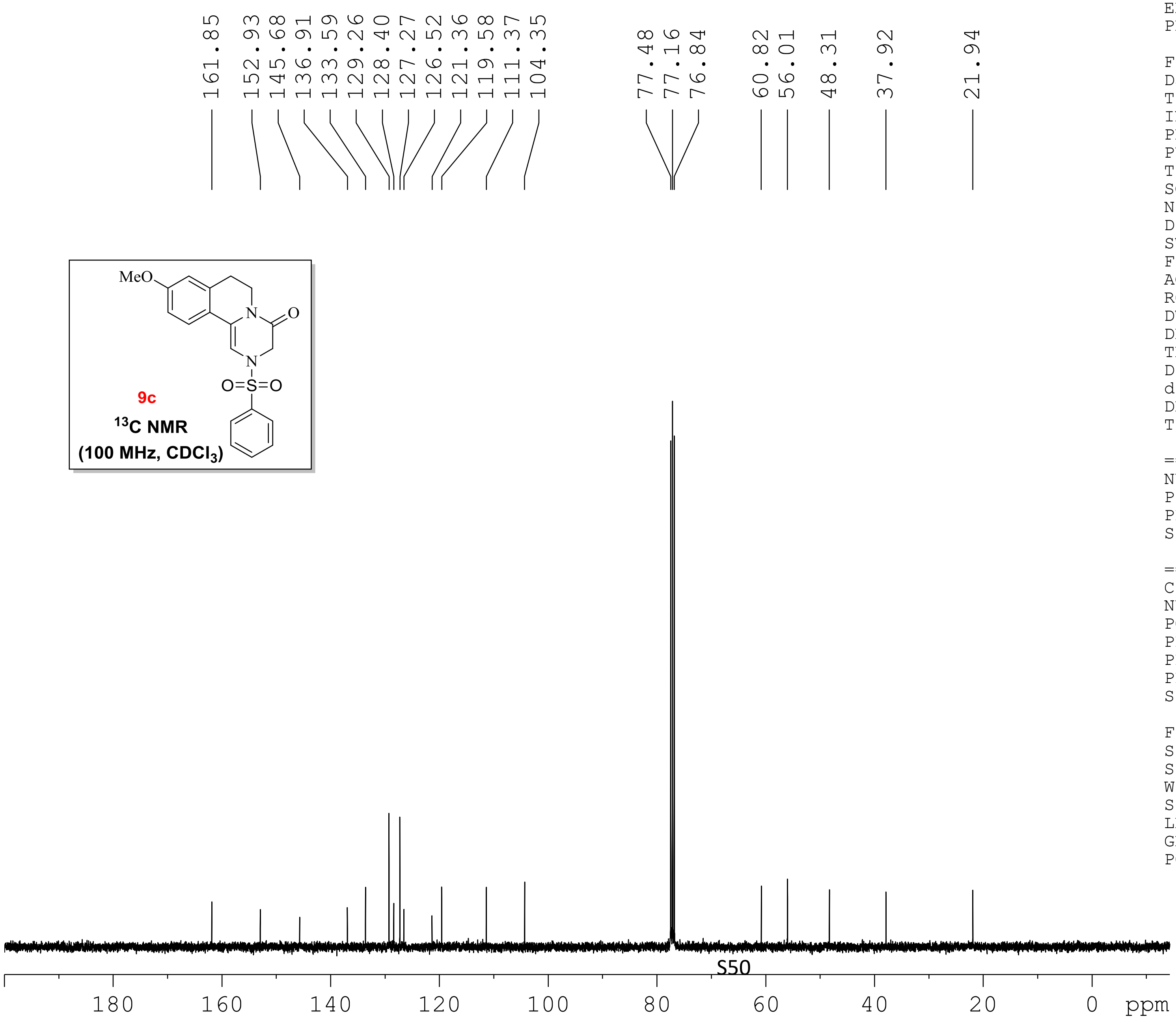

9c

^{13}C NMR

(100 MHz, $CDCl_3$)

```
Current Data Parameters
NAME        RS-I-3-OMe-PzZQ-CY1
EXPNO                     2
PROCNO                    1

F2 - Acquisition Parameters
Date_              20150222
Time                  23.06
INSTRUM               spect
PROBHD     5 mm DUL 13C-1
PULPROG             zgpg30
TD                    65536
SOLVENT               CDCl3
NS                      178
DS                        4
SWH              24038.461 Hz
FIDRES            0.366798 Hz
AQ               1.3631988 sec
RG                     45.2
DW                   20.800 usec
DE                     6.00 usec
TE                    295.7 K
D1               2.00000000 sec
d11              0.03000000 sec
DELTA            1.89999998 sec
TD0                       1

======== CHANNEL f1 ========
NUC1                    13C
P1                     9.15 usec
PL1                    0.00 dB
SFO1            100.6228298 MHz

======== CHANNEL f2 ========
CPDPRG2             waltz16
NUC2                     1H
PCPD2                 90.00 usec
PL12                  14.90 dB
PL13                  14.90 dB
PL2                   -3.00 dB
SFO2            400.1316005 MHz

F2 - Processing parameters
SI                    32768
SF              100.6127553 MHz
WDW                      EM
SSB                       0
LB                     1.00 Hz
GB                        0
PC                     1.40
```

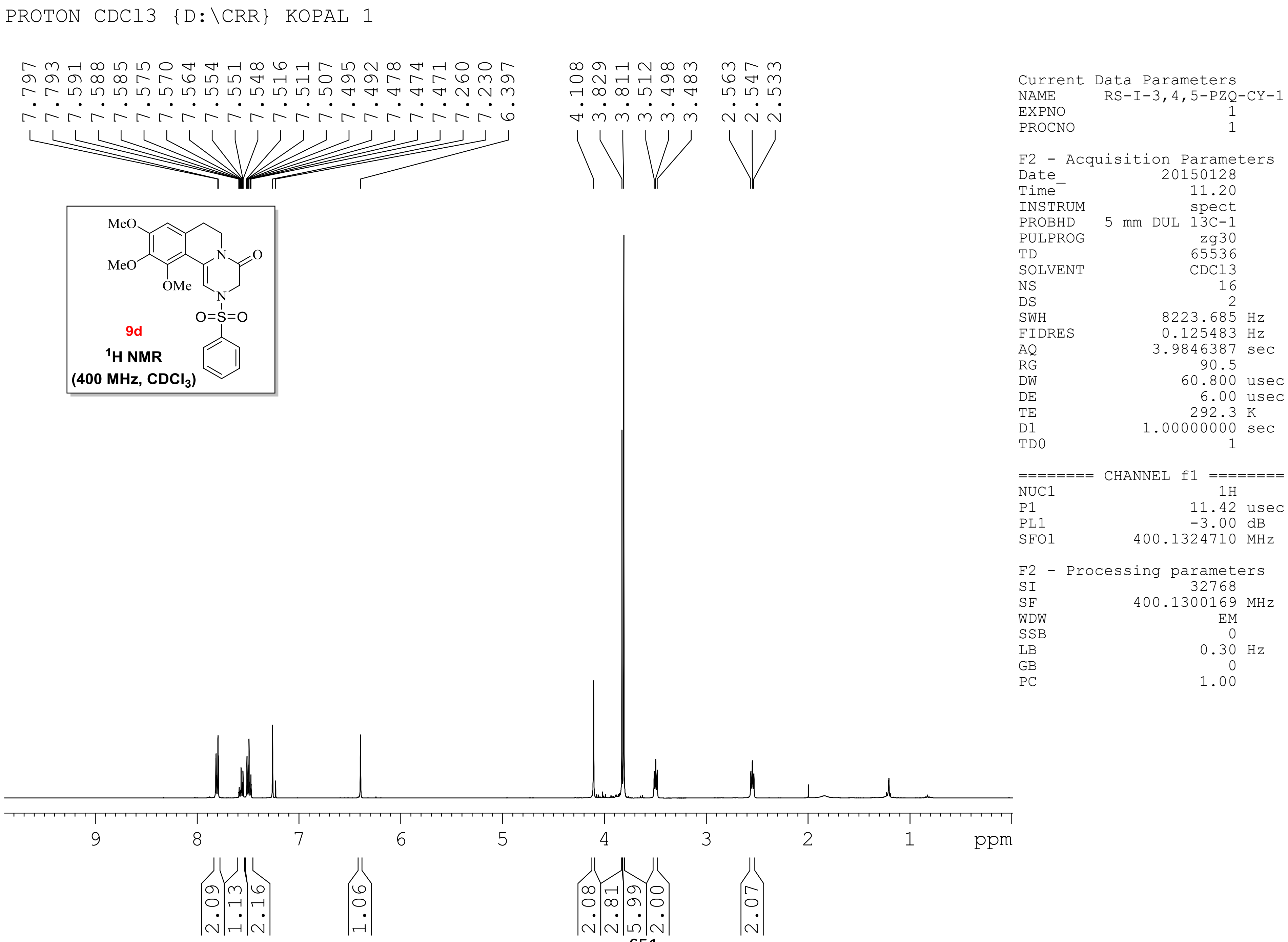
PROTON CDCl3 {D:\CRR} KOPAL 1
7.797 7.793 7.591 7.588 7.585 7.575 7.570 7.564 7.554 7.551 7.548 7.516 7.511 7.507 7.495 7.492 7.478 7.474 7.471 7.260 7.230 6.397
4.108 3.829 3.811 3.512 3.498 3.483 2.563 2.547 2.533
MeO
MeO
OMe
N
O
N
O=S=O
9d
1H NMR
(400 MHz, CDCl3)
Current Data Parameters
NAME RS-I-3,4,5-PZQ-CY-1
EXPNO 1
PROCNO 1
F2 - Acquisition Parameters
Date_ 20150128
Time 11.20
INSTRUM spect
PROBHD 5 mm DUL 13C-1
PULPROG zg30
TD 65536
SOLVENT CDCl3
NS 16
DS 2
SWH 8223.685 Hz
FIDRES 0.125483 Hz
AQ 3.9846387 sec
RG 90.5
DW 60.800 usec
DE 6.00 usec
TE 292.3 K
D1 1.00000000 sec
TD0 1
======== CHANNEL f1 ========
NUC1 1H
P1 11.42 usec
PL1 -3.00 dB
SFO1 400.1324710 MHz
F2 - Processing parameters
SI 32768
SF 400.1300169 MHz
WDW EM
SSB 0
LB 0.30 Hz
GB 0
PC 1.00
9 8 7 6 5 4 3 2 1 ppm
2.09 1.13 2.16 1.06 2.08 2.81 5.99 2.00 2.07

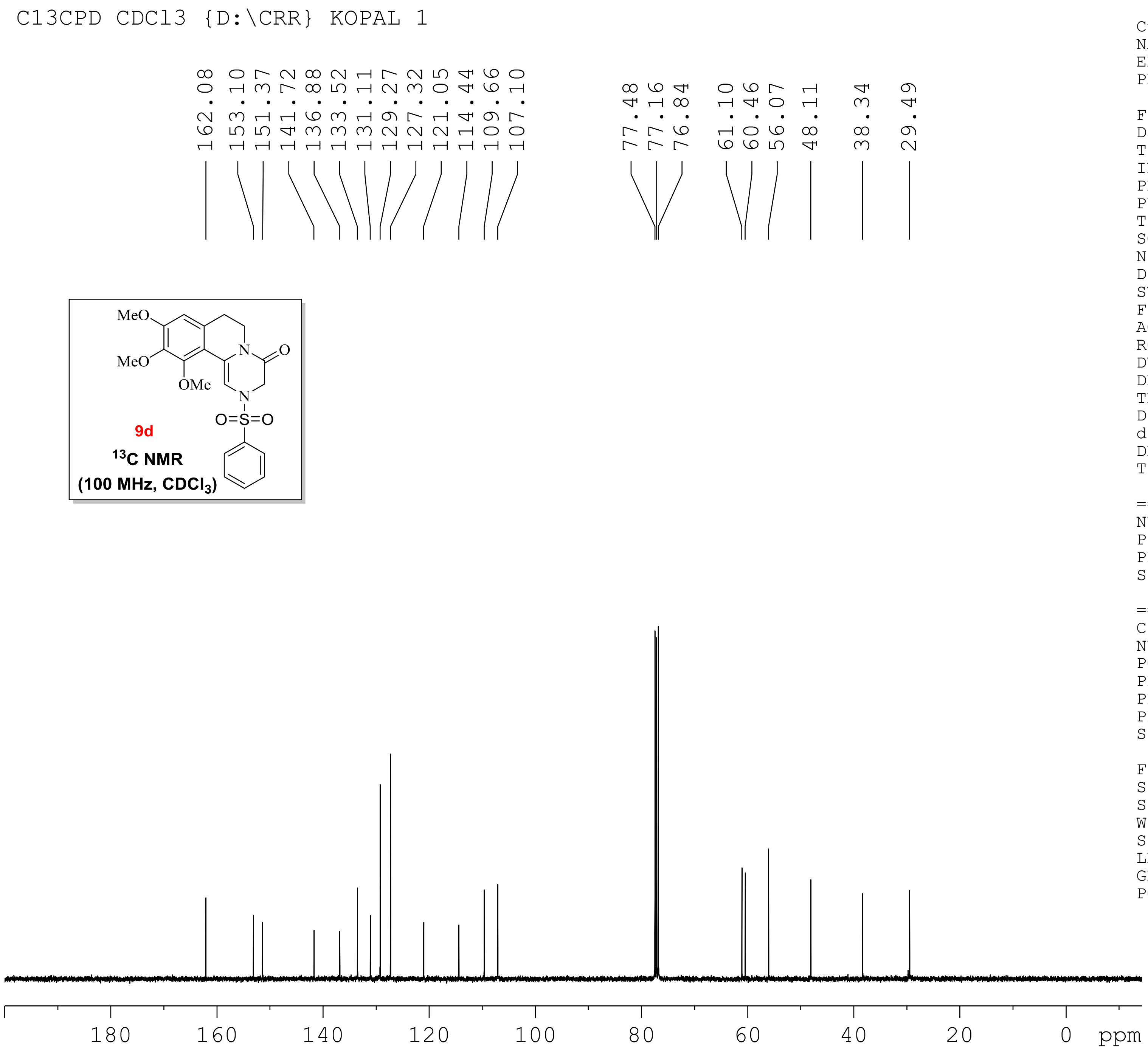

Current Data Parameters
NAME RS-I-3,4,5-PZQ-CY-1
EXPNO 2
PROCNO 1

F2 - Acquisition Parameters
Date_ 20150128
Time 11.30
INSTRUM spect
PROBHD 5 mm DUL 13C-1
PULPROG zgpg30
TD 65536
SOLVENT CDCl3
NS 256
DS 4
SWH 24038.461 Hz
FIDRES 0.366798 Hz
AQ 1.3631988 sec
RG 64
DW 20.800 usec
DE 6.00 usec
TE 293.5 K
D1 2.00000000 sec
d11 0.03000000 sec
DELTA 1.89999998 sec
TD0 1

======== CHANNEL f1 ========
NUC1 13C
P1 9.15 usec
PL1 0.00 dB
SFO1 100.6228298 MHz

======== CHANNEL f2 ========
CPDPRG2 waltz16
NUC2 1H
PCPD2 90.00 usec
PL12 14.90 dB
PL13 14.90 dB
PL2 -3.00 dB
SFO2 400.1316005 MHz

F2 - Processing parameters
SI 32768
SF 100.6127595 MHz
WDW EM
SSB 0
LB 1.00 Hz
GB 0
PC 1.40

PROTON CDCl3 {D:\CRR} KOPAL 1

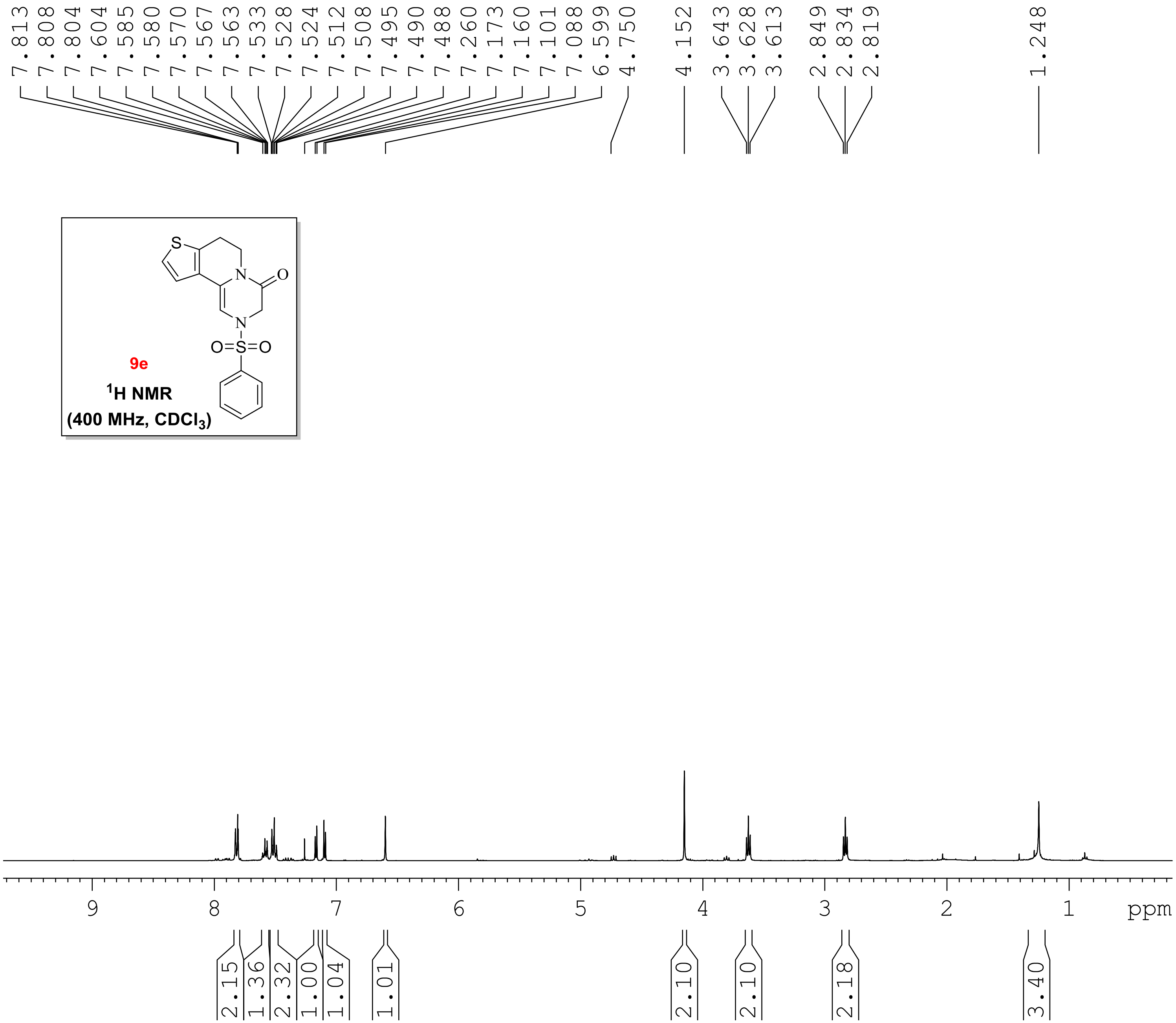

Current Data Parameters
NAME RS-I-THIO-PZQ-CY-1
EXPNO 1
PROCNO 1

F2 - Acquisition Parameters
Date_ 20150226
Time 15.24
INSTRUM spect
PROBHD 5 mm DUL 13C-1
PULPROG zg30
TD 65536
SOLVENT CDCl3
NS 16
DS 2
SWH 8223.685 Hz
FIDRES 0.125483 Hz
AQ 3.9846387 sec
RG 128
DW 60.800 usec
DE 6.00 usec
TE 295.5 K
D1 1.00000000 sec
TD0 1

======== CHANNEL f1 ========
NUC1 1H
P1 11.42 usec
PL1 -3.00 dB
SFO1 400.1324710 MHz

F2 - Processing parameters
SI 32768
SF 400.1300051 MHz
WDW EM
SSB 0
LB 0.30 Hz
GB 0
PC 1.00

C13CPD CDCl3 {D:\CRR} KOPAL 1

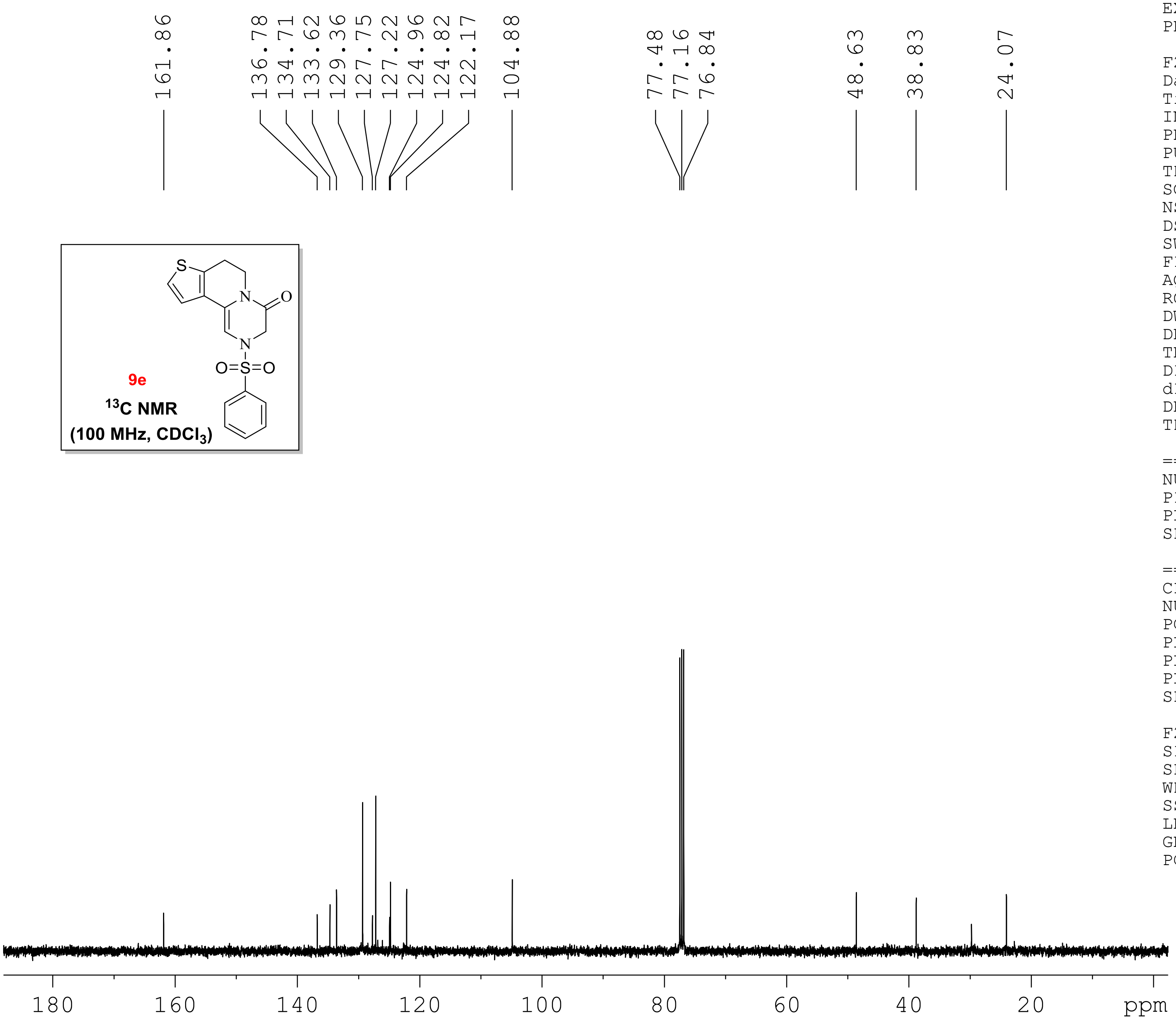

Current Data Parameters
NAME RS-I-THIO-PZQ-CY-1
EXPNO 2
PROCNO 1

F2 - Acquisition Parameters
Date_ 20150226
Time 15.27
INSTRUM spect
PROBHD 5 mm DUL 13C-1
PULPROG zgpg30
TD 65536
SOLVENT CDCl3
NS 63
DS 4
SWH 24038.461 Hz
FIDRES 0.366798 Hz
AQ 1.3631988 sec
RG 32
DW 20.800 usec
DE 6.00 usec
TE 296.1 K
D1 2.00000000 sec
d11 0.03000000 sec
DELTA 1.89999998 sec
TD0 1

======== CHANNEL f1 ========
NUC1 13C
P1 9.15 usec
PL1 0.00 dB
SFO1 100.6228298 MHz

======== CHANNEL f2 ========
CPDPRG2 waltz16
NUC2 1H
PCPD2 90.00 usec
PL12 14.90 dB
PL13 14.90 dB
PL2 -3.00 dB
SFO2 400.1316005 MHz

F2 - Processing parameters
SI 32768
SF 100.6127574 MHz
WDW EM
SSB 0
LB 1.00 Hz
GB 0
PC 1.40

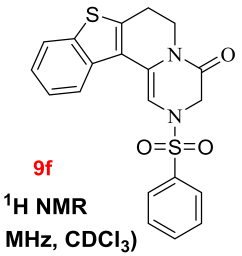

9f

^{1}H NMR

(400 MHz, $CDCl_3$)

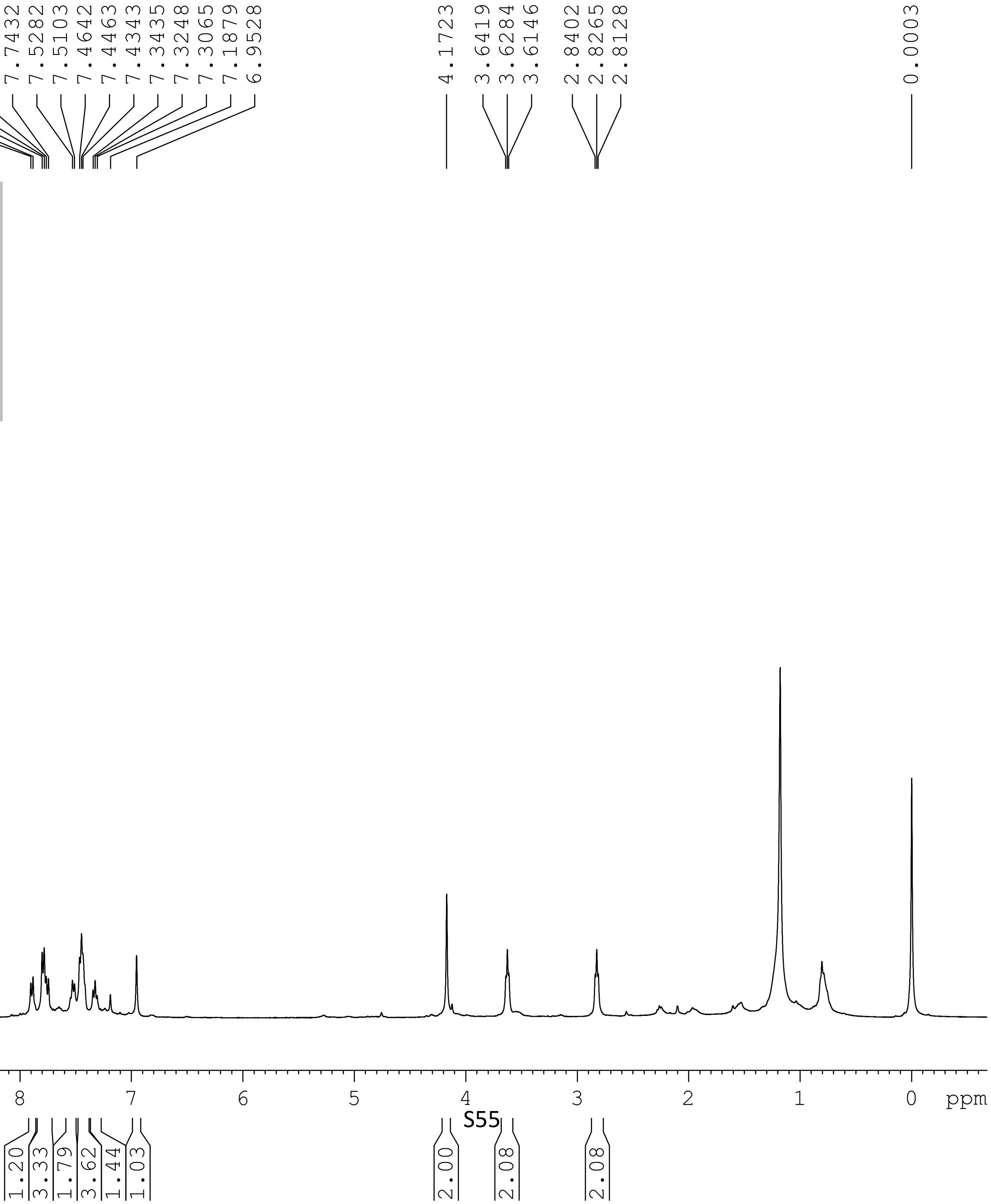

Current Data Parameters	
NAME	RS-I-BT-PZQ-CY-1
EXPNO	1
PROCNO	1
F2 - Acquisition Parameters	
Date_	20150726
Time	13.07
INSTRUM	spect
PROBHD	5 mm DUL 13C-1
PULPROG	zg30
TD	65536
SOLVENT	CDCl3
NS	16
DS	2
SWH	8223.685 Hz
FIDRES	0.125483 Hz
AQ	3.9846387 sec
RG	114
DW	60.800 usec
DE	6.00 usec
TE	294.0 K
D1	1.00000000 sec
TD0	1
======== CHANNEL f1 ========	
NUC1	1H
P1	11.42 usec
PL1	-3.00 dB
SFO1	400.1324710 MHz
F2 - Processing parameters	
SI	32768
SF	400.1300338 MHz
WDW	EM
SSB	0
LB	0.30 Hz
GB	0
PC	1.00

C13CPD CDCl3 {D:\CRR} KOPAL 1

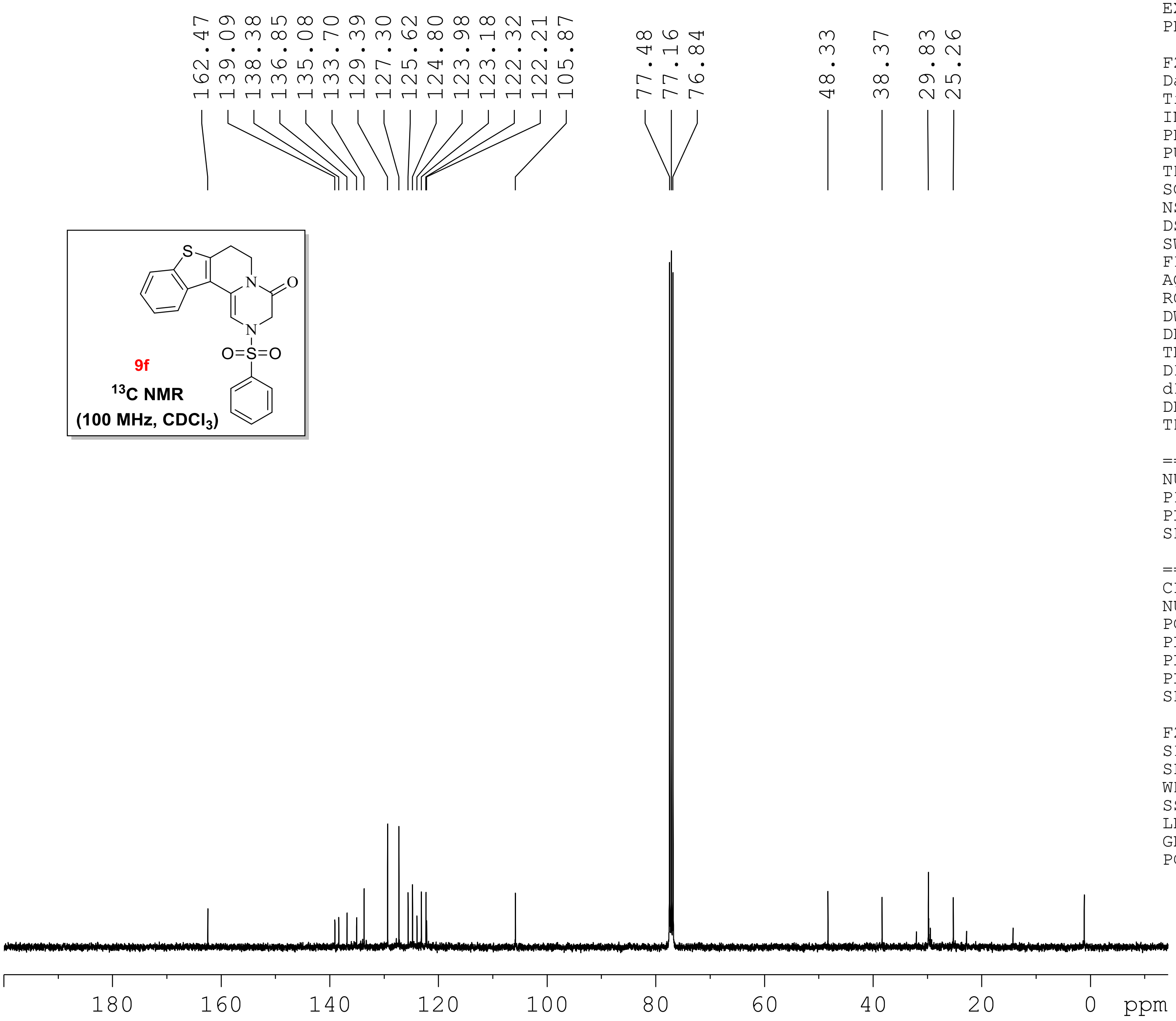

Current Data Parameters
NAME RS-I-BT-PZQ-CY-1
EXPNO 2
PROCNO 1

F2 - Acquisition Parameters
Date_ 20150726
Time 13.14
INSTRUM spect
PROBHD 5 mm DUL 13C-1
PULPROG zgpg30
TD 65536
SOLVENT CDCl3
NS 1024
DS 4
SWH 24038.461 Hz
FIDRES 0.366798 Hz
AQ 1.3631988 sec
RG 57
DW 20.800 usec
DE 6.00 usec
TE 294.7 K
D1 2.00000000 sec
d11 0.03000000 sec
DELTA 1.89999998 sec
TD0 1

======== CHANNEL f1 ========
NUC1 13C
P1 9.15 usec
PL1 0.00 dB
SFO1 100.6228298 MHz

======== CHANNEL f2 ========
CPDPRG2 waltz16
NUC2 1H
PCPD2 90.00 usec
PL12 14.90 dB
PL13 14.90 dB
PL2 -3.00 dB
SFO2 400.1316005 MHz

F2 - Processing parameters
SI 32768
SF 100.6127560 MHz
WDW EM
SSB 0
LB 1.00 Hz
GB 0
PC 1.40

PROTON CDCl3 {D:\CRR} KOPAL 1

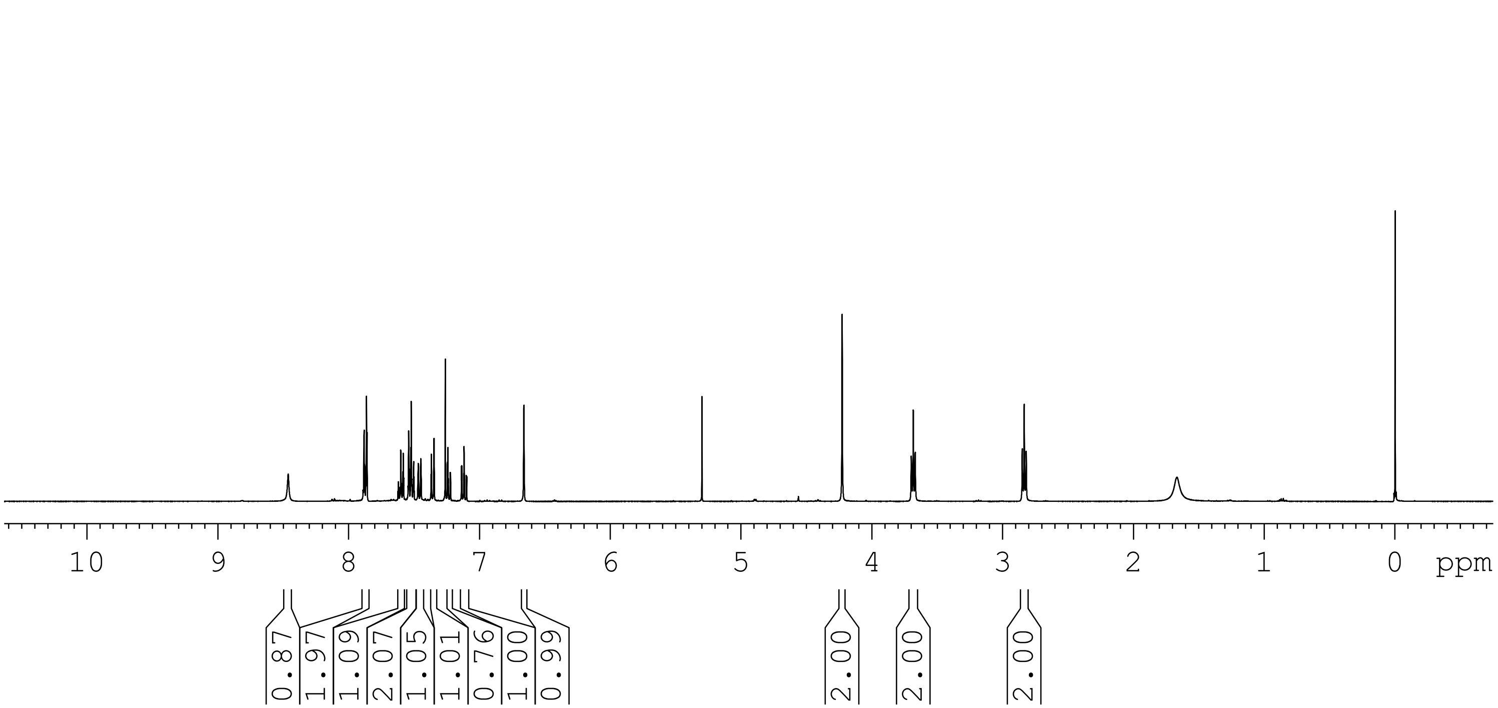

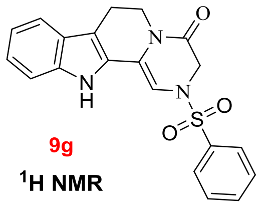

9g

^{1}H NMR

(400 MHz, $CDCl_3$)

Current Data Parameters
NAME RS-I-IN-PZQ-CY
EXPNO 1
PROCNO 1

F2 - Acquisition Parameters
Date_ 20150106
Time 15.00
INSTRUM spect
PROBHD 5 mm DUL 13C-1
PULPROG zg30
TD 65536
SOLVENT CDCl3
NS 16
DS 2
SWH 8223.685 Hz
FIDRES 0.125483 Hz
AQ 3.9846387 sec
RG 256
DW 60.800 usec
DE 6.00 usec
TE 296.8 K
D1 1.00000000 sec
TD0 1

======== CHANNEL f1 ========
NUC1 1H
P1 11.42 usec
PL1 -3.00 dB
SFO1 400.1324710 MHz

F2 - Processing parameters
SI 32768
SF 400.1300051 MHz
WDW EM
SSB 0
LB 0.30 Hz
GB 0
PC 1.00

C13CPD DMSO {D:\CRR} KOPAL 1

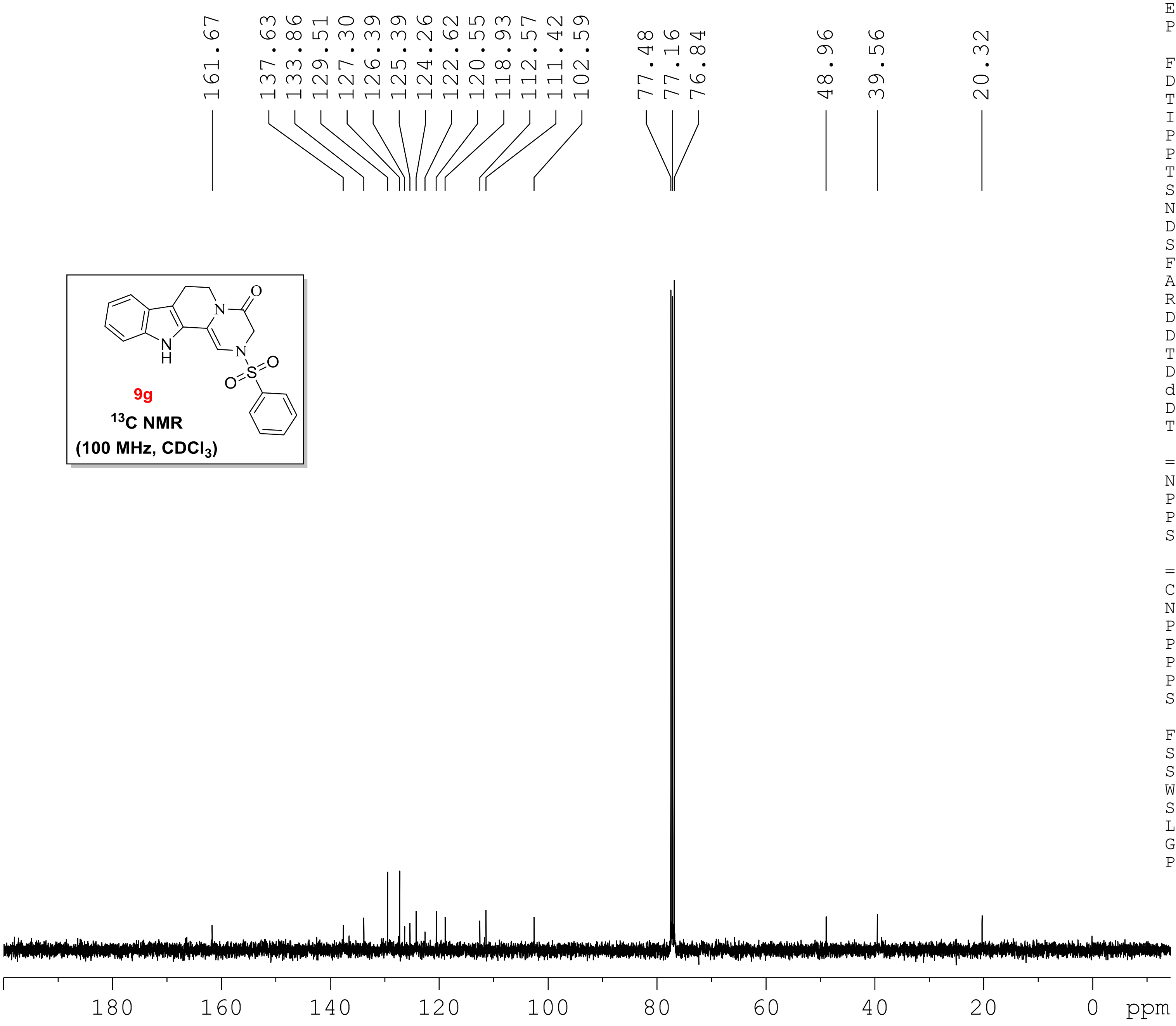

Current Data Parameters
NAME RS-I-IN-PZQ-CY
EXPNO 2
PROCNO 1

F2 - Acquisition Parameters
Date_ 20150106
Time 16.52
INSTRUM spect
PROBHD 5 mm DUL 13C-1
PULPROG zgpg30
TD 65536
SOLVENT DMSO
NS 217
DS 4
SWH 24038.461 Hz
FIDRES 0.366798 Hz
AQ 1.3631988 sec
RG 50.8
DW 20.800 usec
DE 6.00 usec
TE 298.0 K
D1 2.00000000 sec
d11 0.03000000 sec
DELTA 1.89999998 sec
TD0 1

======== CHANNEL f1 ========
NUC1 13C
P1 9.15 usec
PL1 0.00 dB
SFO1 100.6228298 MHz

======== CHANNEL f2 ========
CPDPRG2 waltz16
NUC2 1H
PCPD2 90.00 usec
PL12 14.90 dB
PL13 14.90 dB
PL2 -3.00 dB
SFO2 400.1316005 MHz

F2 - Processing parameters
SI 32768
SF 100.6122739 MHz
WDW EM
SSB 0
LB 1.00 Hz
GB 0
PC 1.40

PROTON CDCl3 {D:\CRR} KOPAL 1

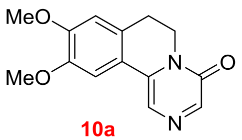

10a

^{1}H NMR

(400 MHz, $CDCl_3$)

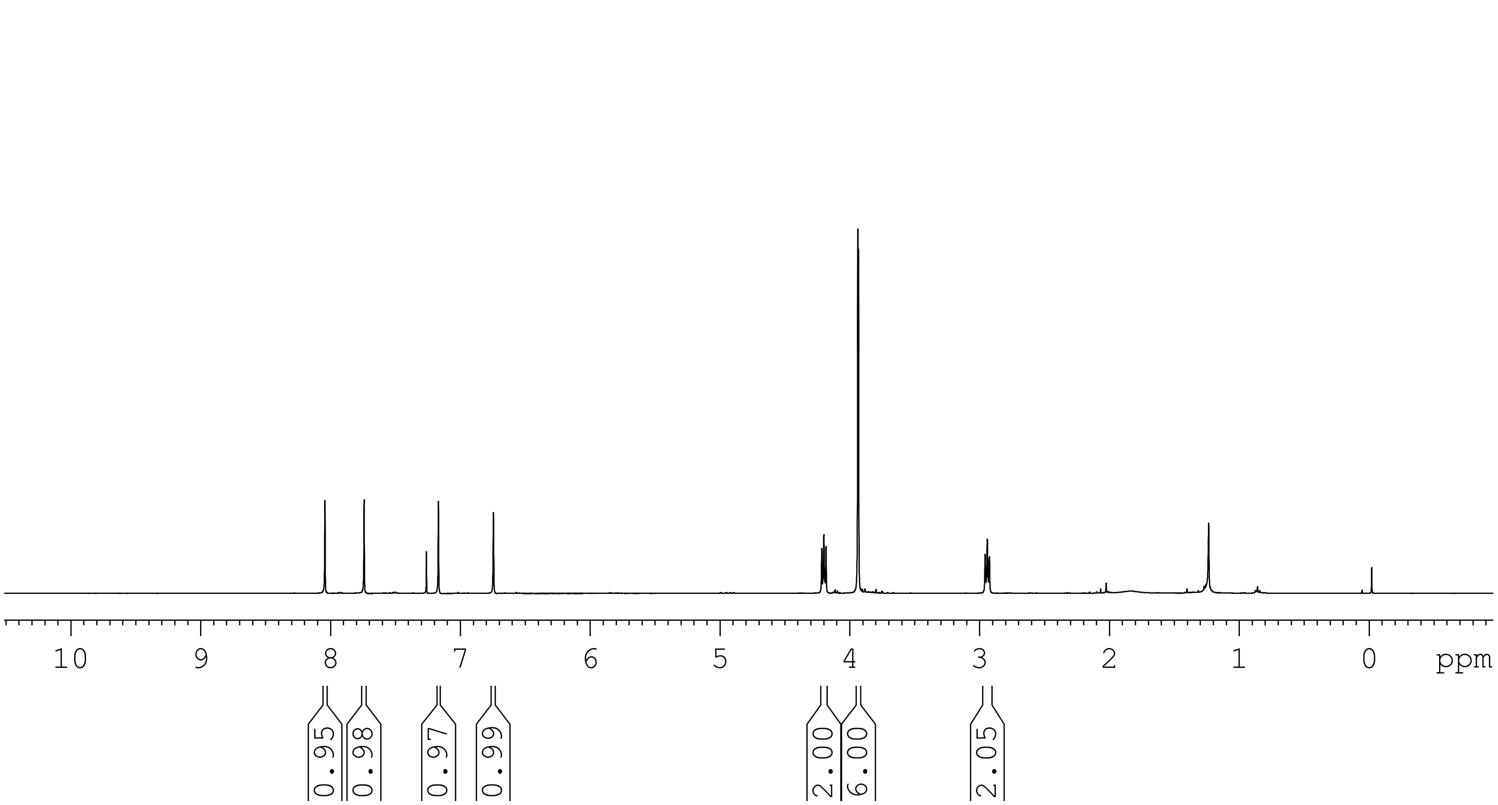


```
Current Data Parameters
NAME       RS-I-PZQ-IM-CY-B
EXPNO                     1
PROCNO                    1

F2 - Acquisition Parameters
Date_              20130711
Time                  12.51
INSTRUM               spect
PROBHD     5 mm BBO BB-1H
PULPROG                zg30
TD                    65536
SOLVENT               CDCl3
NS                       16
DS                        2
SWH                8223.685 Hz
FIDRES             0.125483 Hz
AQ                3.9846387 sec
RG                      256
DW                   60.800 usec
DE                     6.00 usec
TE                    296.9 K
D1               1.00000000 sec
TD0                       1

======== CHANNEL f1 ========
NUC1                     1H
P1                    14.50 usec
PL1                   -0.90 dB
SFO1            400.1324710 MHz

F2 - Processing parameters
SI                    32768
SF              400.1300056 MHz
WDW                      EM
SSB                       0
LB                     0.30 Hz
GB                        0
PC                     1.00
```

C13CPD CDCl3 {D:\CRR} KOPAL 1

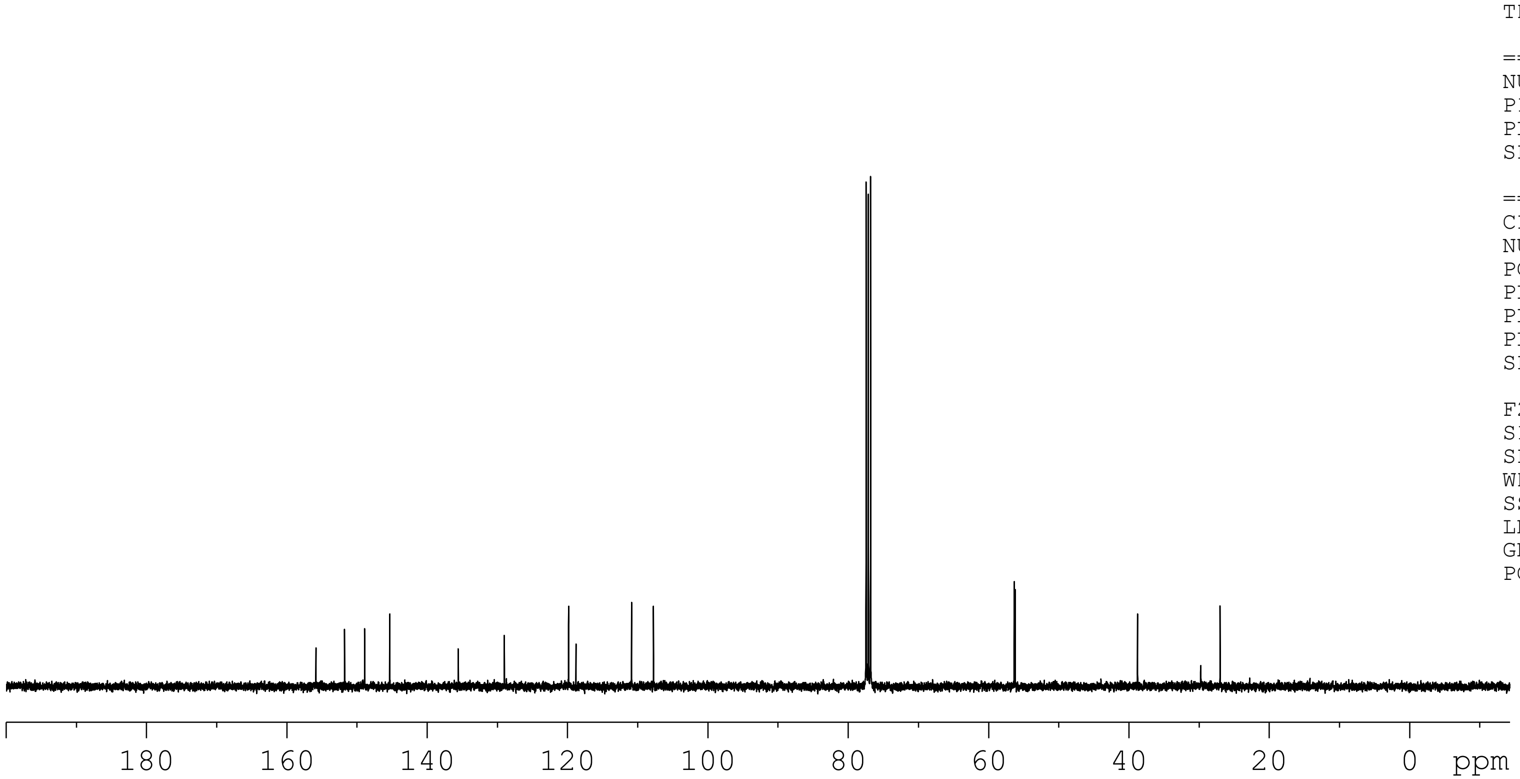

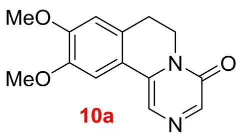

10a

^{13}C NMR

(100 MHz, $CDCl_3$)

```
Current Data Parameters
NAME      RS-I-PZQ-IM-CY-B
EXPNO                    2
PROCNO                   1

F2 - Acquisition Parameters
Date_             20130711
Time                 13.04
INSTRUM              spect
PROBHD   5 mm BBO BB-1H
PULPROG            zgpg30
TD                   65536
SOLVENT              CDCl3
NS                     211
DS                       4
SWH          24038.461 Hz
FIDRES        0.366798 Hz
AQ          1.3631988 sec
RG                     724
DW             20.800 usec
DE               6.00 usec
TE               297.3 K
D1         2.00000000 sec
d11        0.03000000 sec
DELTA      1.89999998 sec
TD0                      1

======== CHANNEL f1 ========
NUC1                   13C
P1               9.50 usec
PL1               -0.60 dB
SFO1       100.6228298 MHz

======== CHANNEL f2 ========
CPDPRG2            waltz16
NUC2                    1H
PCPD2           90.00 usec
PL12              14.96 dB
PL13              15.60 dB
PL2               -0.90 dB
SFO2       400.1316005 MHz

F2 - Processing parameters
SI                   32768
SF         100.6127564 MHz
WDW                     EM
SSB                      0
LB                 1.00 Hz
GB                       0
PC                    1.40
```

PROTON CDCl3 {D:\CRR} KOPAL 1

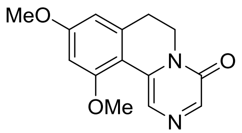

10b

^{1}H NMR

(400 MHz, $CDCl_3$)

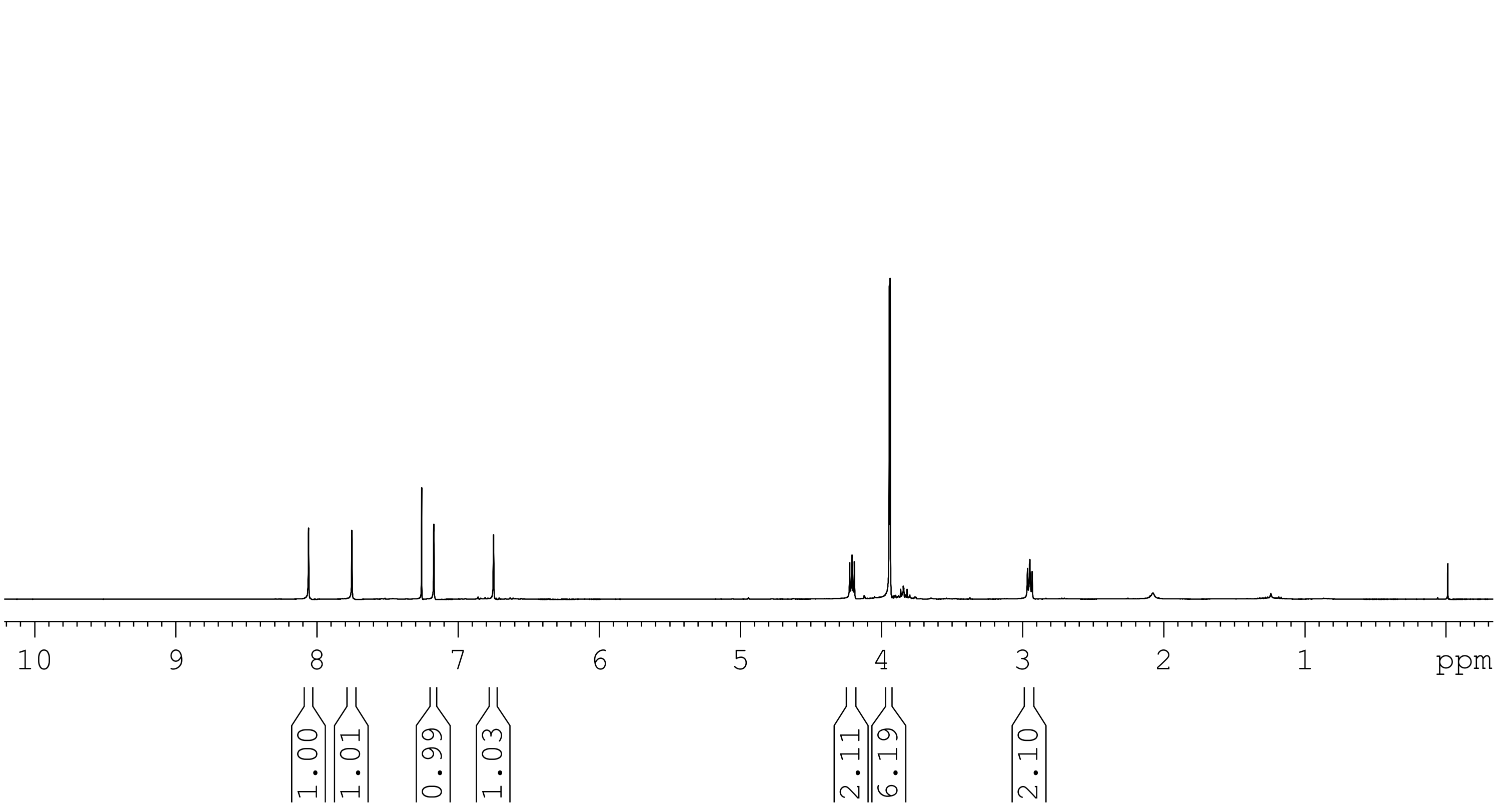


```
Current Data Parameters
NAME       RS-I-3,5-PZQ-CY-2
EXPNO                      2
PROCNO                     1

F2 - Acquisition Parameters
Date_               20150204
Time                   15.56
INSTRUM                spect
PROBHD     5 mm DUL 13C-1
PULPROG                 zg30
TD                     65536
SOLVENT                CDCl3
NS                        16
DS                         2
SWH                 8223.685 Hz
FIDRES              0.125483 Hz
AQ                 3.9846387 sec
RG                       181
DW                    60.800 usec
DE                      6.00 usec
TE                     294.7 K
D1                1.00000000 sec
TD0                        1

======== CHANNEL f1 ========
NUC1                      1H
P1                     11.42 usec
PL1                    -3.00 dB
SFO1             400.1324710 MHz

F2 - Processing parameters
SI                     32768
SF               400.1300051 MHz
WDW                       EM
SSB                        0
LB                      0.30 Hz
GB                         0
PC                      1.00
```

C13CPD CDCl3 {D:\CRR} KOPAL 1

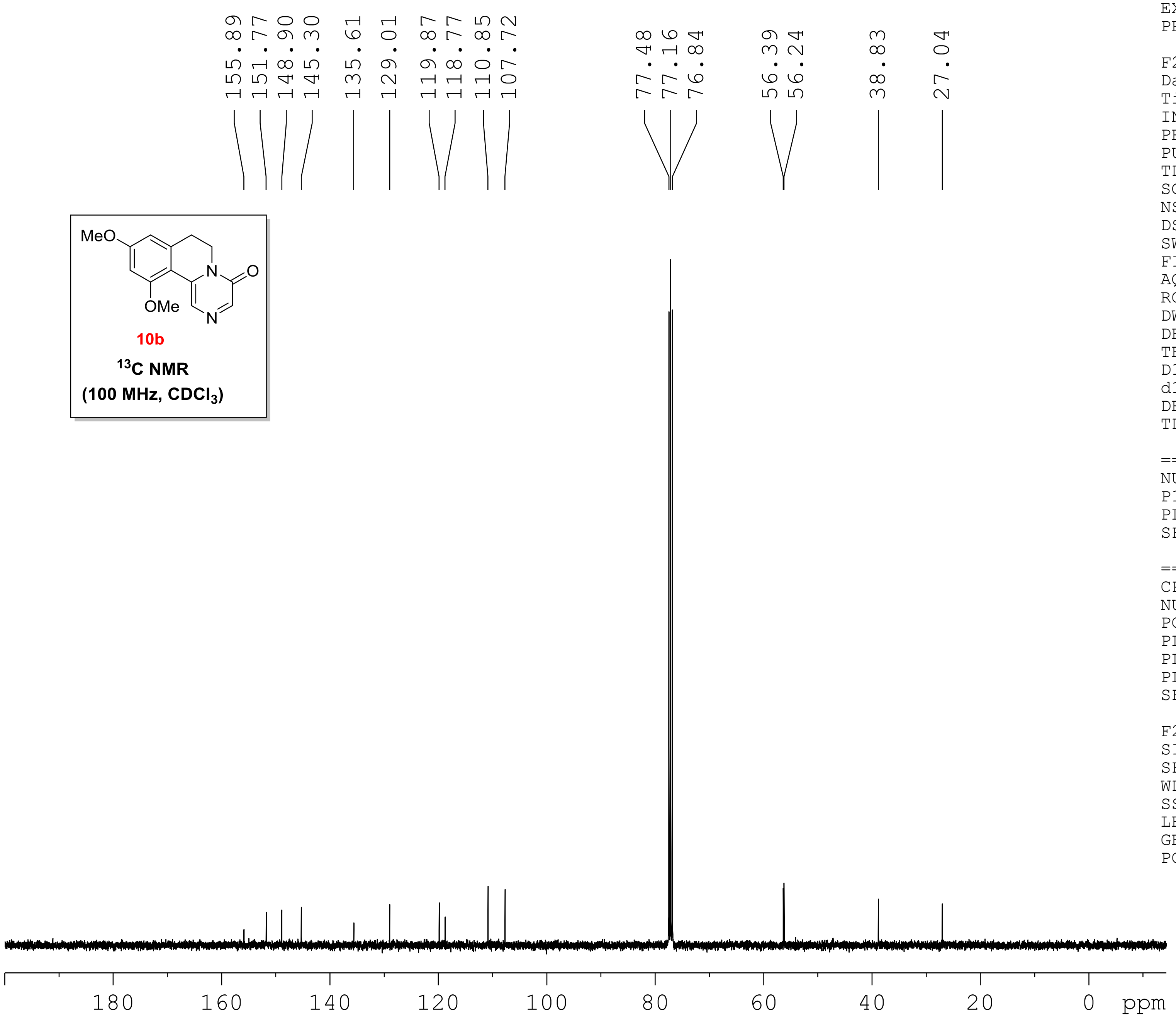


```
Current Data Parameters
NAME      RS-I-3,5-PZQ-CY-2
EXPNO                     3
PROCNO                    1

F2 - Acquisition Parameters
Date_              20150204
Time                  16.10
INSTRUM               spect
PROBHD    5 mm DUL 13C-1
PULPROG              zgpg30
TD                    65536
SOLVENT               CDCl3
NS                      256
DS                        4
SWH              24038.461 Hz
FIDRES            0.366798 Hz
AQ               1.3631988 sec
RG                       32
DW                   20.800 usec
DE                     6.00 usec
TE                    295.0 K
D1               2.00000000 sec
d11              0.03000000 sec
DELTA            1.89999998 sec
TD0                       1

======== CHANNEL f1 ========
NUC1                    13C
P1                     9.15 usec
PL1                    0.00 dB
SFO1            100.6228298 MHz

======== CHANNEL f2 ========
CPDPRG2             waltz16
NUC2                     1H
PCPD2                 90.00 usec
PL12                  14.90 dB
PL13                  14.90 dB
PL2                   -3.00 dB
SFO2            400.1316005 MHz

F2 - Processing parameters
SI                    32768
SF              100.6127553 MHz
WDW                      EM
SSB                       0
LB                     1.00 Hz
GB                        0
PC                     1.40
```

PROTON CDCl3 {D:\CRR} KOPAL 1

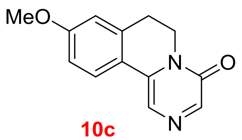

10c

^{1}H NMR

(400 MHz, $CDCl_3$)

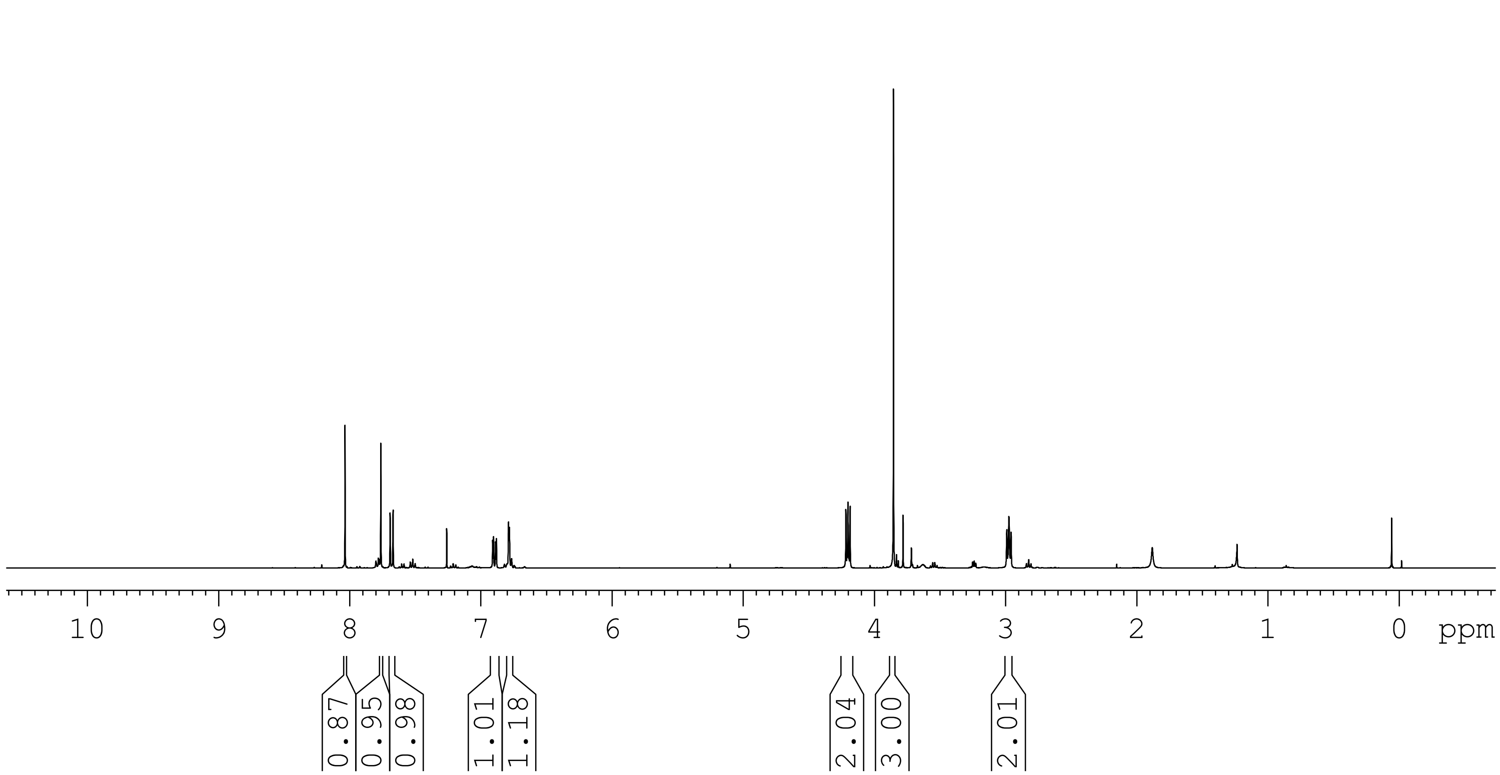


```
Current Data Parameters
NAME     RS-I-2,3-ome-PZ-QCY-2
EXPNO                        1
PROCNO                       1

F2 - Acquisition Parameters
Date_               20150223
Time                   16.40
INSTRUM                spect
PROBHD     5 mm DUL 13C-1
PULPROG                 zg30
TD                     65536
SOLVENT                CDCl3
NS                        16
DS                         2
SWH                 8223.685 Hz
FIDRES              0.125483 Hz
AQ                 3.9846387 sec
RG                       144
DW                    60.800 usec
DE                      6.00 usec
TE                     296.0 K
D1                1.00000000 sec
TD0                        1

======== CHANNEL f1 ========
NUC1                      1H
P1                     11.42 usec
PL1                    -3.00 dB
SFO1             400.1324710 MHz

F2 - Processing parameters
SI                     32768
SF               400.1300051 MHz
WDW                       EM
SSB                        0
LB                      0.30 Hz
GB                         0
PC                      1.00
```

C13CPD CDCl3 {D:\CRR} KOPAL 1

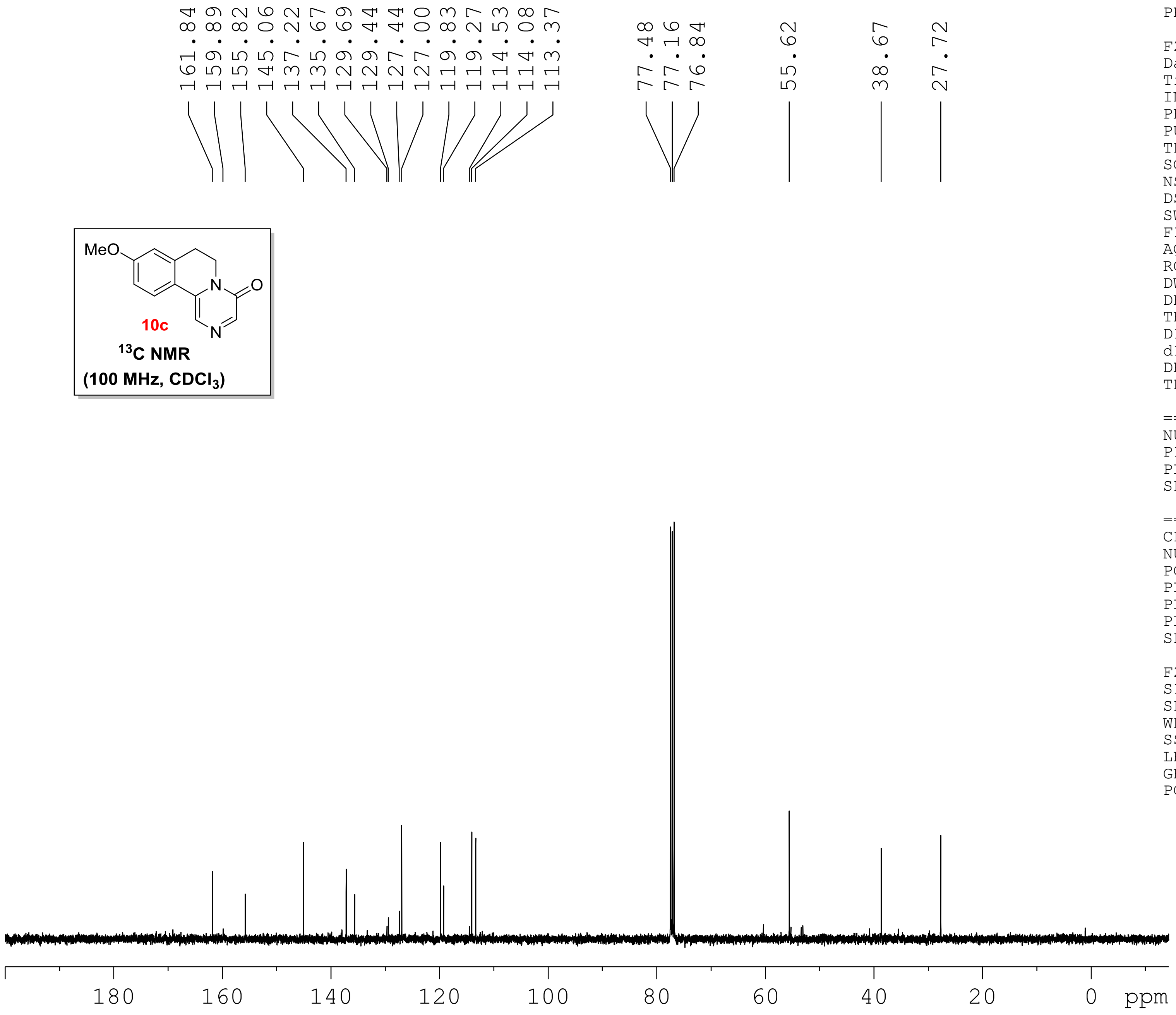

10c

^{13}C NMR

(100 MHz, $CDCl_3$)

Current Data Parameters
NAME RS-I-2,3-ome-PZ-QCY-2
EXPNO 2
PROCNO 1

F2 - Acquisition Parameters
Date_ 20150223
Time 16.47
INSTRUM spect
PROBHD 5 mm DUL 13C-1
PULPROG zgpg30
TD 65536
SOLVENT CDCl3
NS 130
DS 4
SWH 24038.461 Hz
FIDRES 0.366798 Hz
AQ 1.3631988 sec
RG 57
DW 20.800 usec
DE 6.00 usec
TE 296.7 K
D1 2.00000000 sec
d11 0.03000000 sec
DELTA 1.89999998 sec
TD0 1

======== CHANNEL f1 ========
NUC1 13C
P1 9.15 usec
PL1 0.00 dB
SFO1 100.6228298 MHz

======== CHANNEL f2 ========
CPDPRG2 waltz16
NUC2 1H
PCPD2 90.00 usec
PL12 14.90 dB
PL13 14.90 dB
PL2 -3.00 dB
SFO2 400.1316005 MHz

F2 - Processing parameters
SI 32768
SF 100.6127575 MHz
WDW EM
SSB 0
LB 1.00 Hz
GB 0
PC 1.40

PROTON CDCl3 {D:\CRR} KOPAL 1

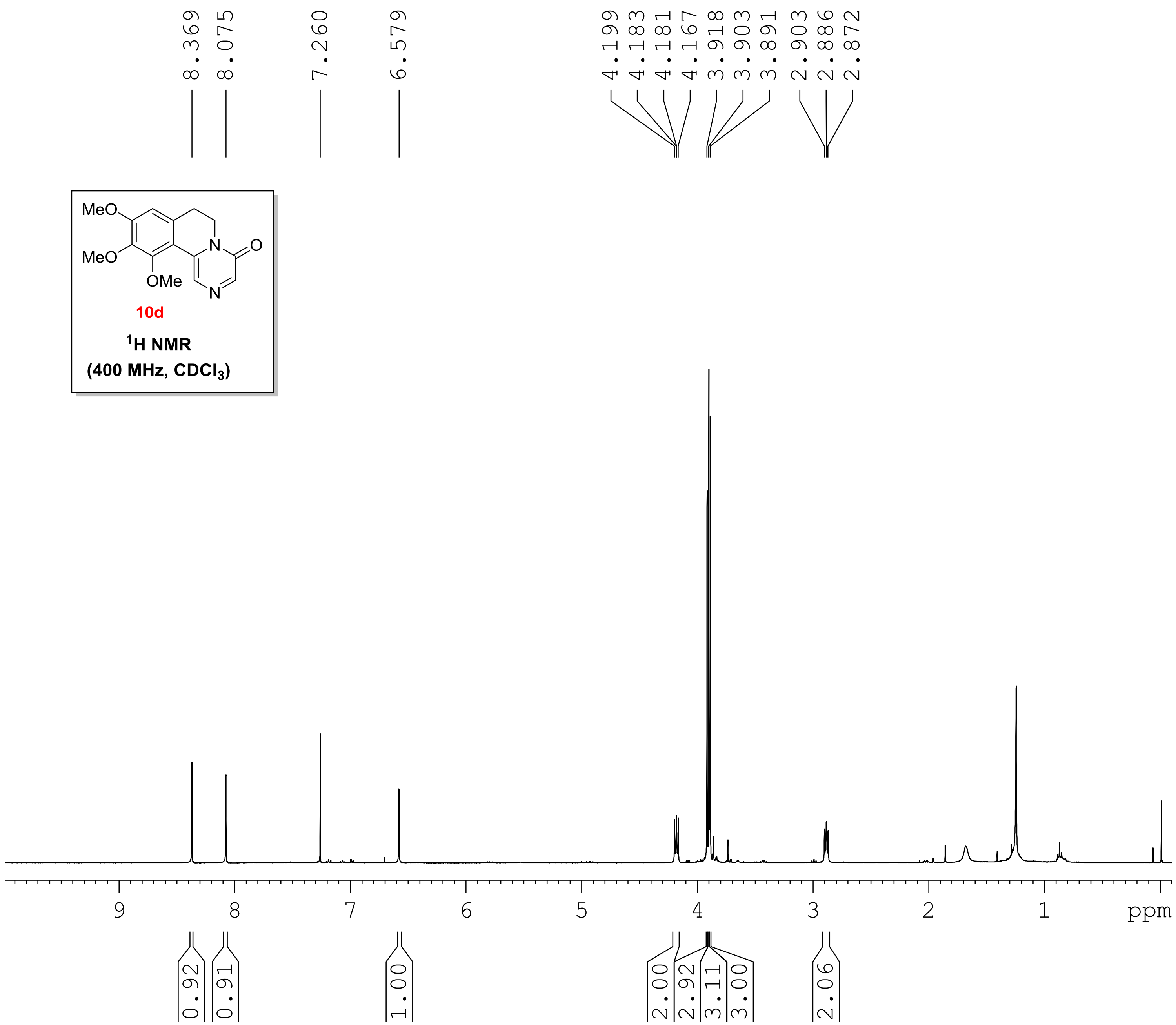

Current Data Parameters
NAME RS-I-3,4,5-PZQ-CY-2
EXPNO 3
PROCNO 1

F2 - Acquisition Parameters
Date_ 20150203
Time 10.57
INSTRUM spect
PROBHD 5 mm DUL 13C-1
PULPROG zg30
TD 65536
SOLVENT CDCl3
NS 16
DS 2
SWH 8223.685 Hz
FIDRES 0.125483 Hz
AQ 3.9846387 sec
RG 203
DW 60.800 usec
DE 6.00 usec
TE 296.1 K
D1 1.00000000 sec
TD0 1

======== CHANNEL f1 ========
NUC1 1H
P1 11.42 usec
PL1 -3.00 dB
SFO1 400.1324710 MHz

F2 - Processing parameters
SI 32768
SF 400.1300051 MHz
WDW EM
SSB 0
LB 0.30 Hz
GB 0
PC 1.00

C13CPD CDCl3 {D:\CRR} KOPAL 1

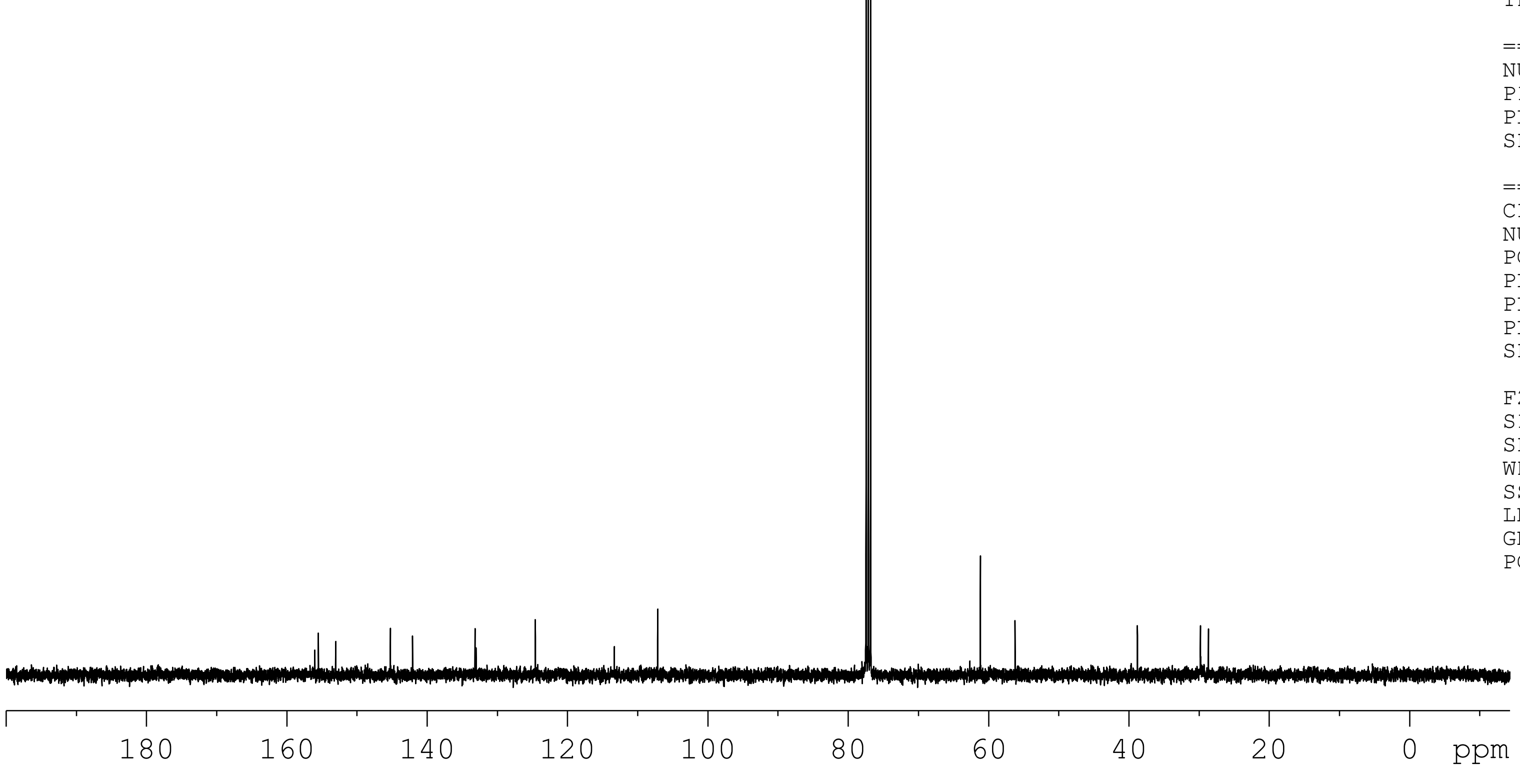

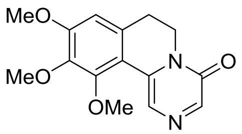

10d

^{13}C NMR

(100 MHz, $CDCl_3$)

Current Data Parameters

NAME	RS-I-3,4,5-PZQ-CY-2
EXPNO	4
PROCNO	1

F2 - Acquisition Parameters

Date_	20150203
Time	10.58
INSTRUM	spect
PROBHD	5 mm DUL 13C-1
PULPROG	zgpg30
TD	65536
SOLVENT	CDCl3
NS	163
DS	4
SWH	24038.461 Hz
FIDRES	0.366798 Hz
AQ	1.3631988 sec
RG	45.2
DW	20.800 usec
DE	6.00 usec
TE	296.7 K
D1	2.00000000 sec
d11	0.03000000 sec
DELTA	1.89999998 sec
TD0	1

======== CHANNEL f1 ========

NUC1	13C
P1	9.15 usec
PL1	0.00 dB
SFO1	100.6228298 MHz

======== CHANNEL f2 ========

CPDPRG2	waltz16
NUC2	1H
PCPD2	90.00 usec
PL12	14.90 dB
PL13	14.90 dB
PL2	-3.00 dB
SFO2	400.1316005 MHz

F2 - Processing parameters

SI	32768
SF	100.6127542 MHz
WDW	EM
SSB	0
LB	1.00 Hz
GB	0
PC	1.40

PROTON CDCl3 {D:\CRR} KOPAL 1

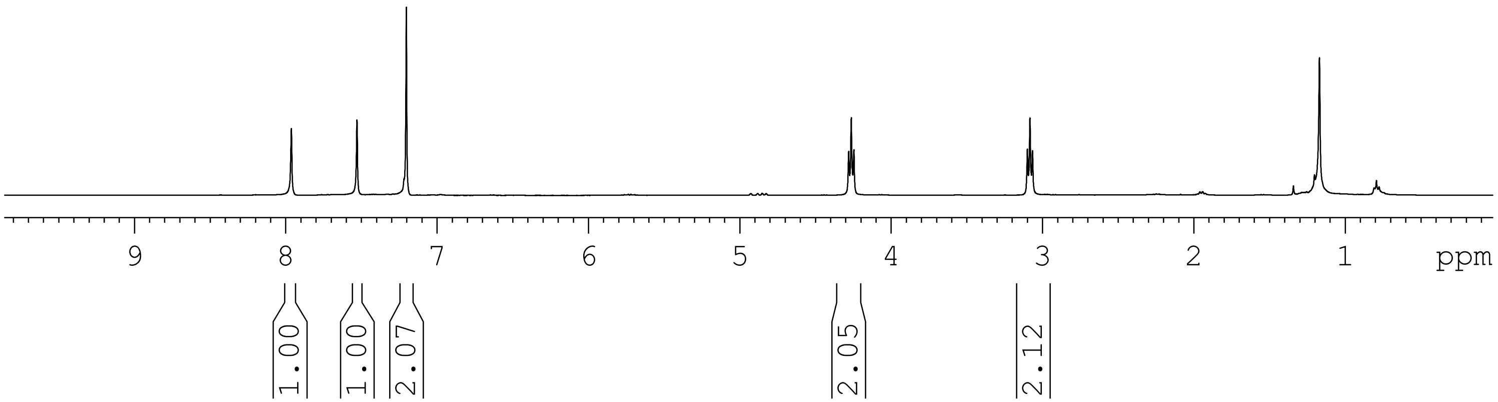

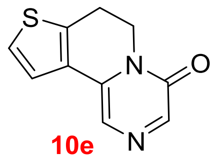

10e

^{1}H NMR
(400 MHz, $CDCl_3$)

```
Current Data Parameters
NAME      RS-I-THIO-PZQ-CY-2
EXPNO                      1
PROCNO                     1

F2 - Acquisition Parameters
Date_              20160227
Time                  15.29
INSTRUM               spect
PROBHD    5 mm DUL 13C-1
PULPROG                zg30
TD                    65536
SOLVENT               CDCl3
NS                       16
DS                        2
SWH                8223.685 Hz
FIDRES             0.125483 Hz
AQ                3.9846387 sec
RG                     80.6
DW                   60.800 usec
DE                     6.00 usec
TE                    295.1 K
D1               1.00000000 sec
TD0                       1

======== CHANNEL f1 ========
NUC1                     1H
P1                    11.42 usec
PL1                   -3.00 dB
SFO1            400.1324710 MHz

F2 - Processing parameters
SI                    32768
SF              400.1300212 MHz
WDW                      EM
SSB                       0
LB                     0.30 Hz
GB                        0
PC                     1.00
```

C13CPD CDCl3 {D:\CRR} KOPAL 1

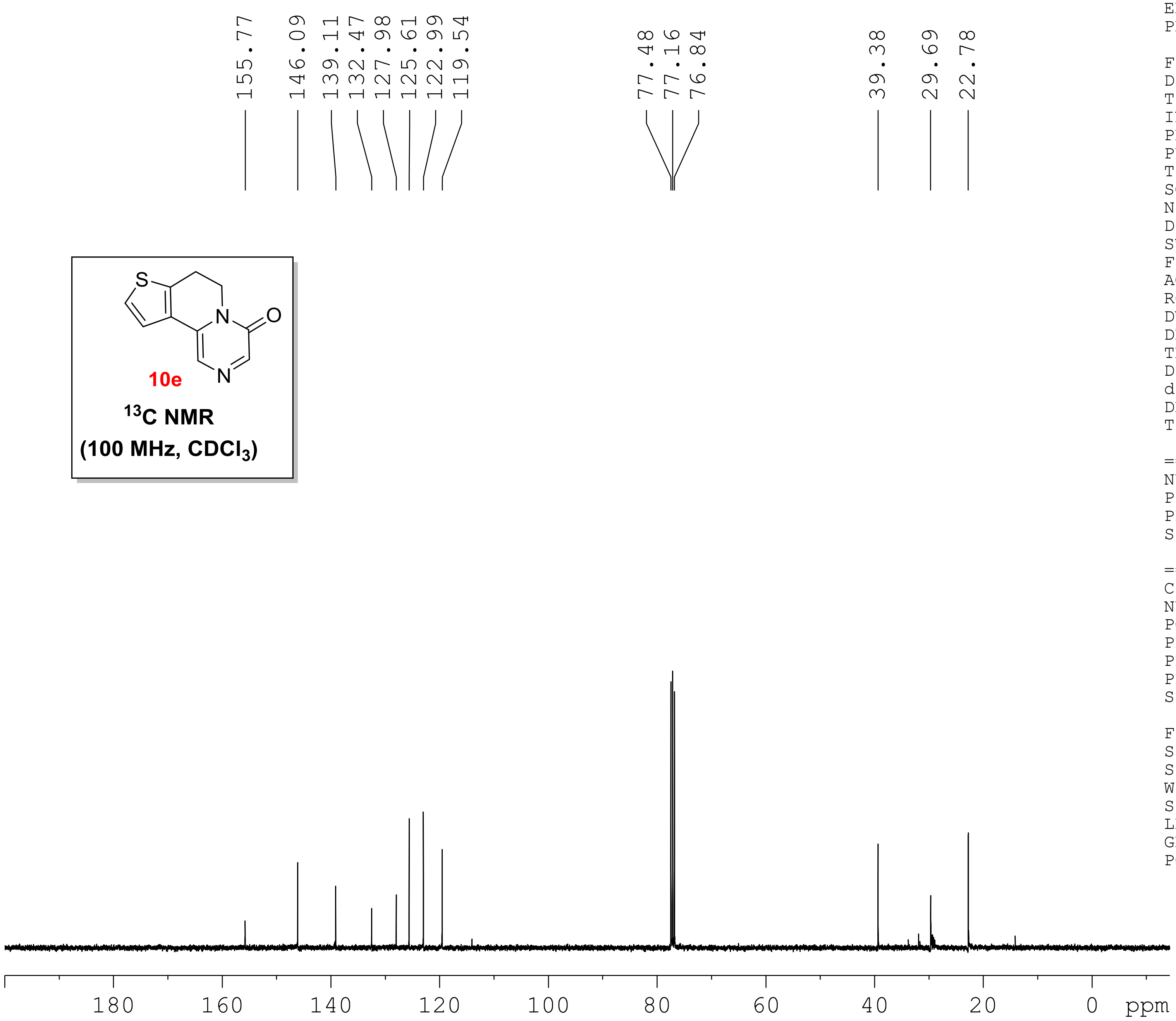

10e

^{13}C NMR

(100 MHz, $CDCl_3$)

```
Current Data Parameters
NAME        RS-I-THIO-PZQ-CY-2
EXPNO                        2
PROCNO                       1

F2 - Acquisition Parameters
Date_                 20160227
Time                     15.33
INSTRUM                  spect
PROBHD      5 mm DUL 13C-1
PULPROG                zgpg30
TD                       65536
SOLVENT                  CDCl3
NS                          73
DS                           4
SWH                  24038.461 Hz
FIDRES                0.366798 Hz
AQ                   1.3631988 sec
RG                          64
DW                      20.800 usec
DE                        6.00 usec
TE                       295.6 K
D1                  2.00000000 sec
d11                 0.03000000 sec
DELTA               1.89999998 sec
TD0                          1

======== CHANNEL f1 ========
NUC1                       13C
P1                        9.15 usec
PL1                       0.00 dB
SFO1               100.6228298 MHz

======== CHANNEL f2 ========
CPDPRG2                waltz16
NUC2                        1H
PCPD2                    90.00 usec
PL12                     14.90 dB
PL13                     14.90 dB
PL2                      -3.00 dB
SFO2               400.1316005 MHz

F2 - Processing parameters
SI                       32768
SF                 100.6127649 MHz
WDW                         EM
SSB                          0
LB                        1.00 Hz
GB                           0
PC                        1.40
```

PROTON CDCl3 {D:\CRR} KOPAL 1

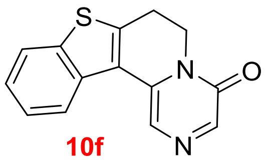

10f

^{1}H NMR

(400 MHz, $CDCl_3$)

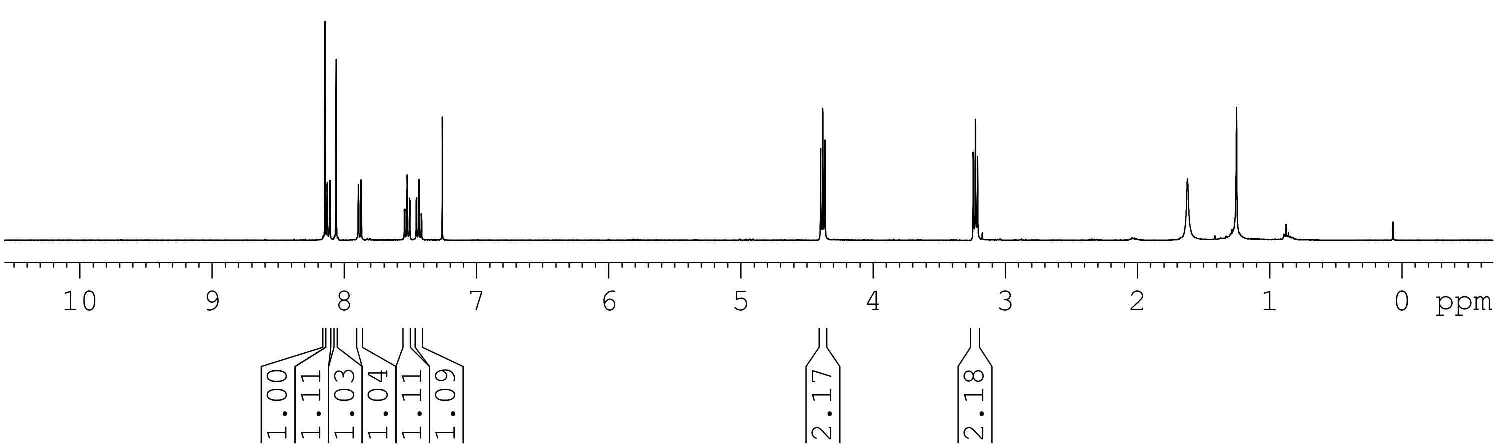

Current Data Parameters
NAME RS-I-BT-PZQ-CY-2
EXPNO 1
PROCNO 1

F2 - Acquisition Parameters
Date_ 20150727
Time 15.36
INSTRUM spect
PROBHD 5 mm DUL 13C-1
PULPROG zg30
TD 65536
SOLVENT CDCl3
NS 12
DS 2
SWH 8223.685 Hz
FIDRES 0.125483 Hz
AQ 3.9846387 sec
RG 256
DW 60.800 usec
DE 6.00 usec
TE 296.5 K
D1 1.00000000 sec
TD0 1

======== CHANNEL f1 ========
NUC1 1H
P1 11.42 usec
PL1 -3.00 dB
SFO1 400.1324710 MHz

F2 - Processing parameters
SI 32768
SF 400.1300051 MHz
WDW EM
SSB 0
LB 0.30 Hz
GB 0
PC 1.00

C13CPD CDCl3 {D:\CRR} KOPAL 1

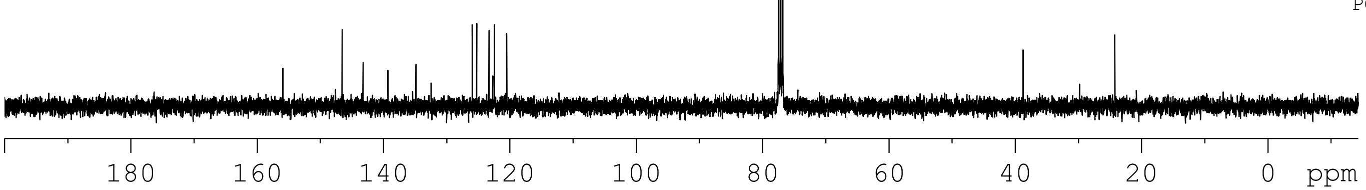

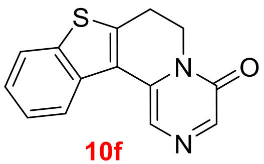

10f

^{13}C NMR

(100 MHz, $CDCl_3$)

```
Current Data Parameters
NAME     RS-I-BT-PZQ-CY-2
EXPNO                   2
PROCNO                  1

F2 - Acquisition Parameters
Date_            20150727
Time                15.40
INSTRUM             spect
PROBHD   5 mm DUL 13C-1
PULPROG            zgpg30
TD                  65536
SOLVENT             CDCl3
NS                    256
DS                      4
SWH             24038.461 Hz
FIDRES           0.366798 Hz
AQ              1.3631988 sec
RG                   50.8
DW                 20.800 usec
DE                   6.00 usec
TE                  297.2 K
D1             2.00000000 sec
d11            0.03000000 sec
DELTA          1.89999998 sec
TD0                     1

======== CHANNEL f1 ========
NUC1                  13C
P1                   9.15 usec
PL1                  0.00 dB
SFO1          100.6228298 MHz

======== CHANNEL f2 ========
CPDPRG2           waltz16
NUC2                   1H
PCPD2               90.00 usec
PL12                14.90 dB
PL13                14.90 dB
PL2                 -3.00 dB
SFO2          400.1316005 MHz

F2 - Processing parameters
SI                  32768
SF            100.6127537 MHz
WDW                    EM
SSB                     0
LB                   1.00 Hz
GB                      0
PC                   1.40
```

PROTON CDCl3 {D:\CRR} KOPAL 1

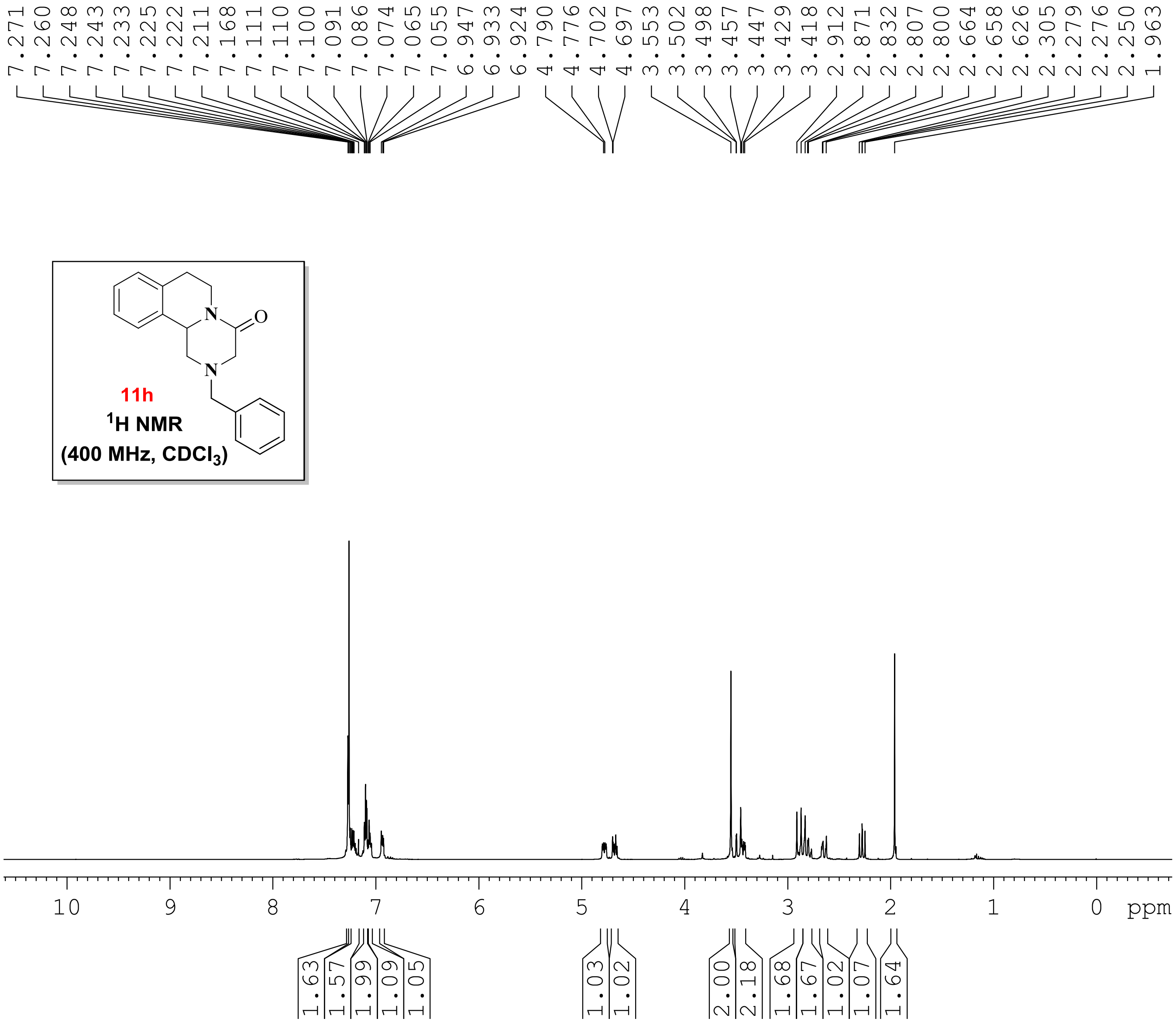

11h
^{1}H NMR
(400 MHz, $CDCl_3$)

```
Current Data Parameters
NAME        RS-I-Un-SUB-PZQ-CY-Bn
EXPNO                     1
PROCNO                    1

F2 - Acquisition Parameters
Date_              20150901
Time                  15.32
INSTRUM               spect
PROBHD    5 mm DUL 13C-1
PULPROG                zg30
TD                    65536
SOLVENT               CDCl3
NS                       16
DS                        2
SWH                8223.685 Hz
FIDRES             0.125483 Hz
AQ                3.9846387 sec
RG                       57
DW                   60.800 usec
DE                     6.00 usec
TE                    296.5 K
D1               1.00000000 sec
TD0                       1

======== CHANNEL f1 ========
NUC1                     1H
P1                    11.42 usec
PL1                   -3.00 dB
SFO1            400.1324710 MHz

F2 - Processing parameters
SI                    32768
SF              400.1300417 MHz
WDW                      EM
SSB                       0
LB                     0.30 Hz
GB                        0
PC                     1.00
```

C13CPD CDCl3 {D:\CRR} KOPAL 1

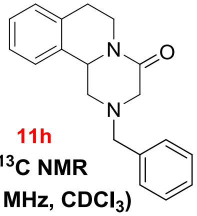

11h

^{13}C NMR

(100 MHz, $CDCl_3$)

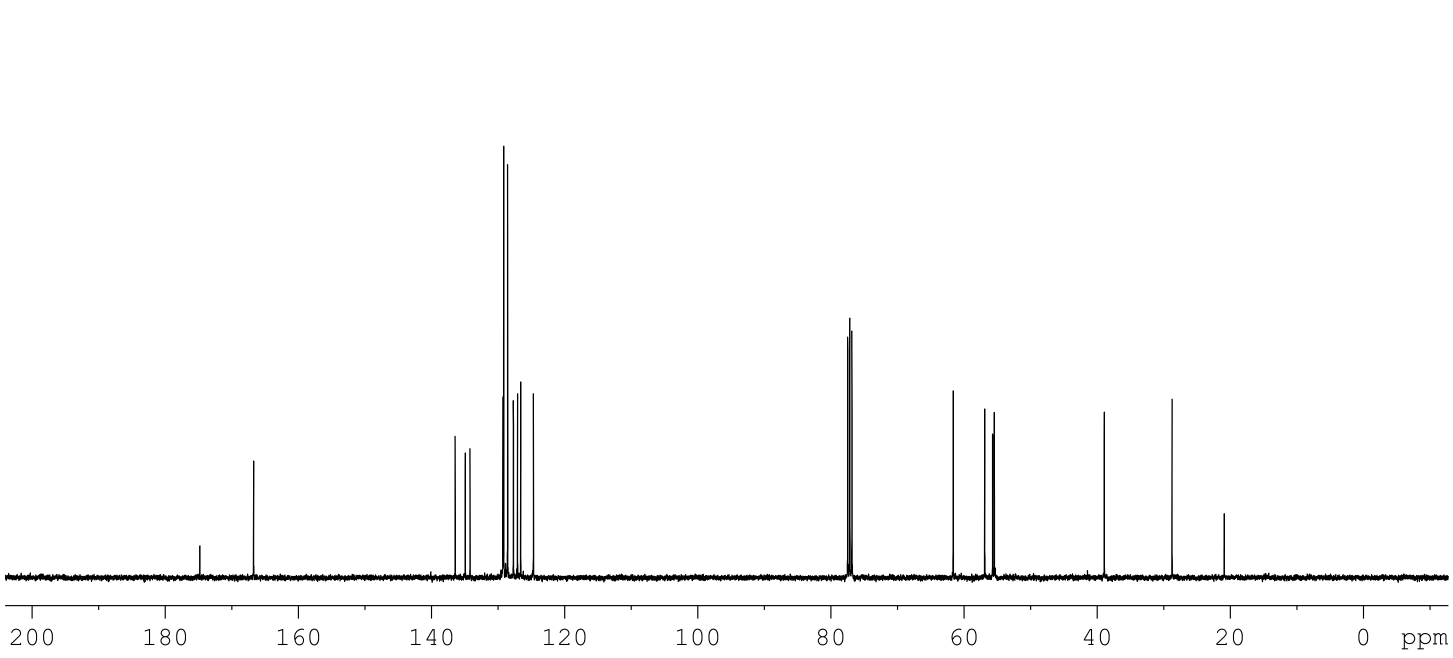

Current Data Parameters
NAME RS-I-Un-SUB-PZQ-CY-Bn
EXPNO 2
PROCNO 1

F2 - Acquisition Parameters
Date_ 20150901
Time 15.43
INSTRUM spect
PROBHD 5 mm DUL 13C-1
PULPROG zgpg30
TD 65536
SOLVENT CDCl3
NS 168
DS 4
SWH 24038.461 Hz
FIDRES 0.366798 Hz
AQ 1.3631988 sec
RG 64
DW 20.800 usec
DE 6.00 usec
TE 297.3 K
D1 2.00000000 sec
d11 0.03000000 sec
DELTA 1.89999998 sec
TD0 1

======== CHANNEL f1 ========
NUC1 13C
P1 9.15 usec
PL1 0.00 dB
SFO1 100.6228298 MHz

======== CHANNEL f2 ========
CPDPRG2 waltz16
NUC2 1H
PCPD2 90.00 usec
PL12 14.90 dB
PL13 14.90 dB
PL2 -3.00 dB
SFO2 400.1316005 MHz

F2 - Processing parameters
SI 32768
SF 100.6127684 MHz
WDW EM
SSB 0
LB 1.00 Hz
GB 0
PC 1.40

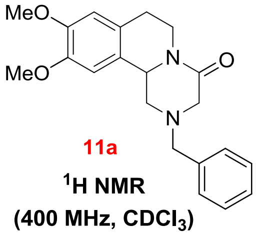

11a

^{1}H NMR

(400 MHz, $CDCl_3$)

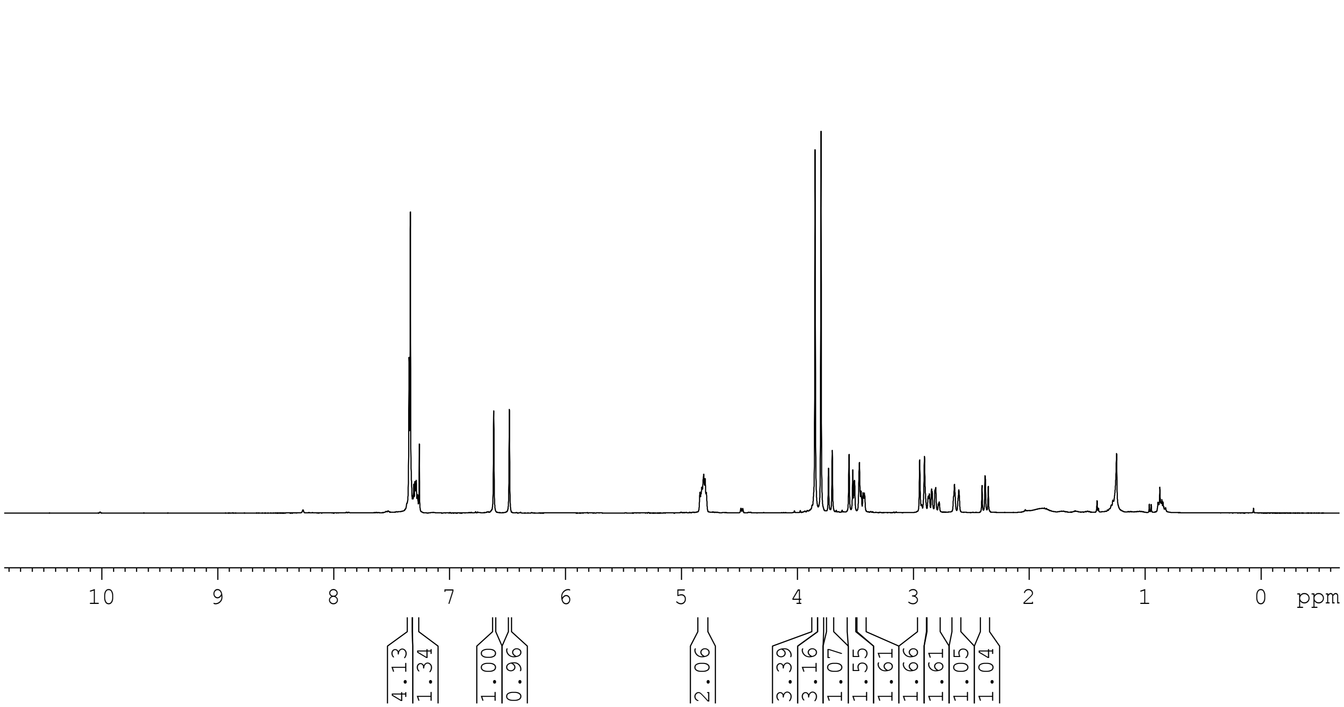


```
Current Data Parameters
NAME      RS-I-3,4-BN-PZQ-CY
EXPNO                  1
PROCNO                 1

F2 - Acquisition Parameters
Date_           20150924
Time               14.17
INSTRUM            spect
PROBHD   5 mm DUL 13C-1
PULPROG             zg30
TD                 65536
SOLVENT            CDCl3
NS                    16
DS                     2
SWH             8223.685 Hz
FIDRES          0.125483 Hz
AQ             3.9846387 sec
RG                   114
DW                60.800 usec
DE                  6.00 usec
TE                 294.7 K
D1            1.00000000 sec
TD0                    1

======== CHANNEL f1 ========
NUC1                  1H
P1                 11.42 usec
PL1                -3.00 dB
SFO1         400.1324710 MHz

F2 - Processing parameters
SI                 32768
SF           400.1300050 MHz
WDW                   EM
SSB                    0
LB                  0.30 Hz
GB                     0
PC                  1.00
```

C13CPD CDCl3 {D:\CRR} KOPAL 1

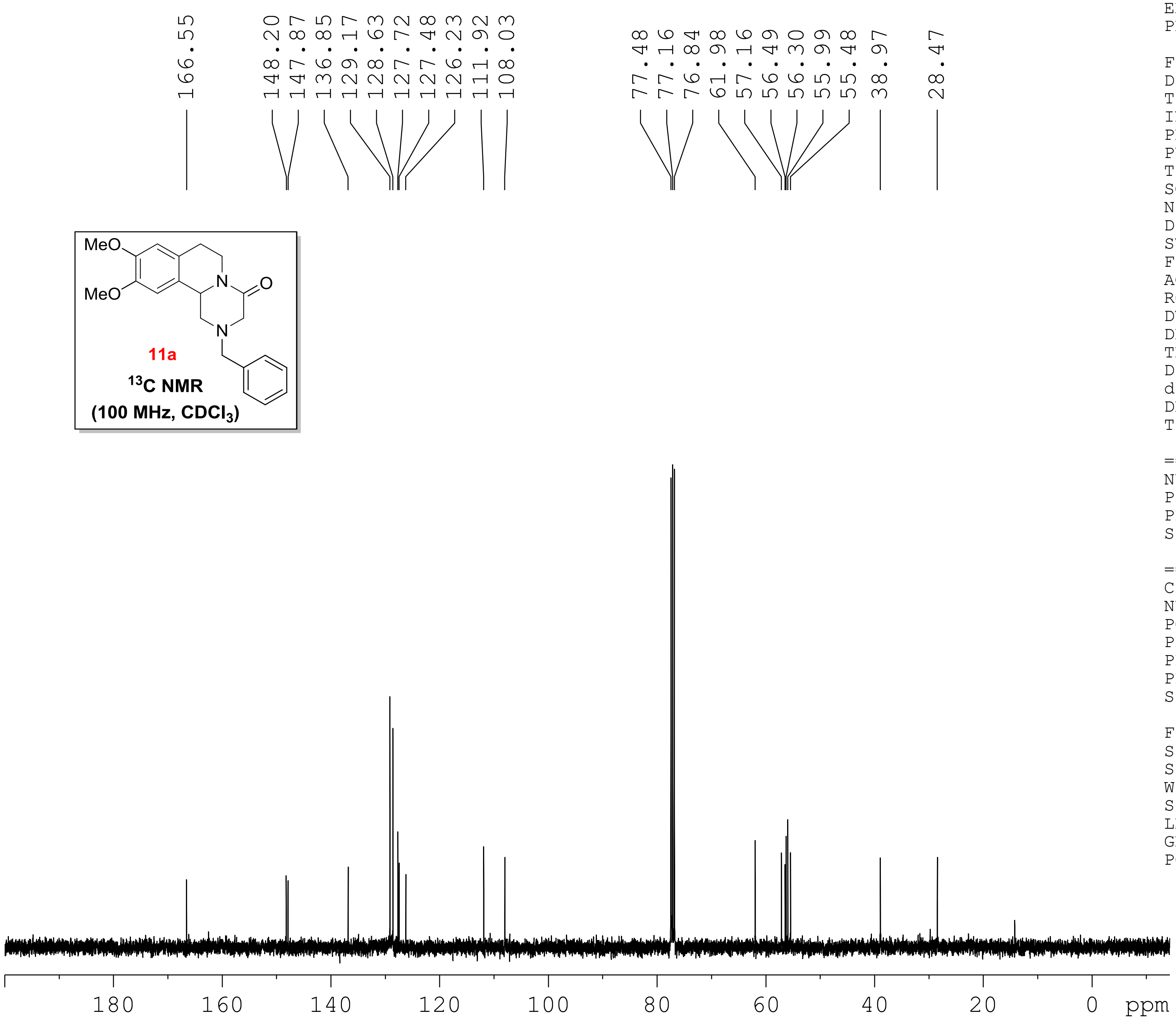


```
Current Data Parameters
NAME       RS-I-3,4-BN-PZQ-CY
EXPNO                       2
PROCNO                      1

F2 - Acquisition Parameters
Date_                20150924
Time                    14.36
INSTRUM                 spect
PROBHD     5 mm DUL 13C-1
PULPROG               zgpg30
TD                      65536
SOLVENT                 CDCl3
NS                         65
DS                          4
SWH                 24038.461 Hz
FIDRES               0.366798 Hz
AQ                  1.3631988 sec
RG                         57
DW                     20.800 usec
DE                       6.00 usec
TE                      295.5 K
D1                 2.00000000 sec
d11                0.03000000 sec
DELTA              1.89999998 sec
TD0                         1

======== CHANNEL f1 ========
NUC1                      13C
P1                       9.15 usec
PL1                      0.00 dB
SFO1              100.6228298 MHz

======== CHANNEL f2 ========
CPDPRG2               waltz16
NUC2                       1H
PCPD2                   90.00 usec
PL12                    14.90 dB
PL13                    14.90 dB
PL2                     -3.00 dB
SFO2              400.1316005 MHz

F2 - Processing parameters
SI                      32768
SF                100.6127580 MHz
WDW                        EM
SSB                         0
LB                       1.00 Hz
GB                          0
PC                       1.40
```

PROTON CDCl3 {D:\CRR} KOPAL 1

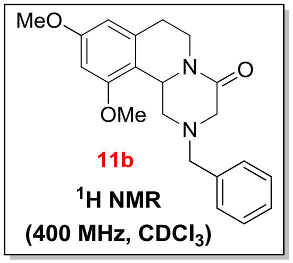

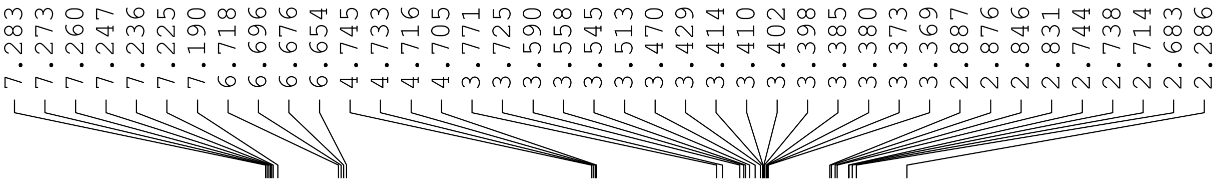

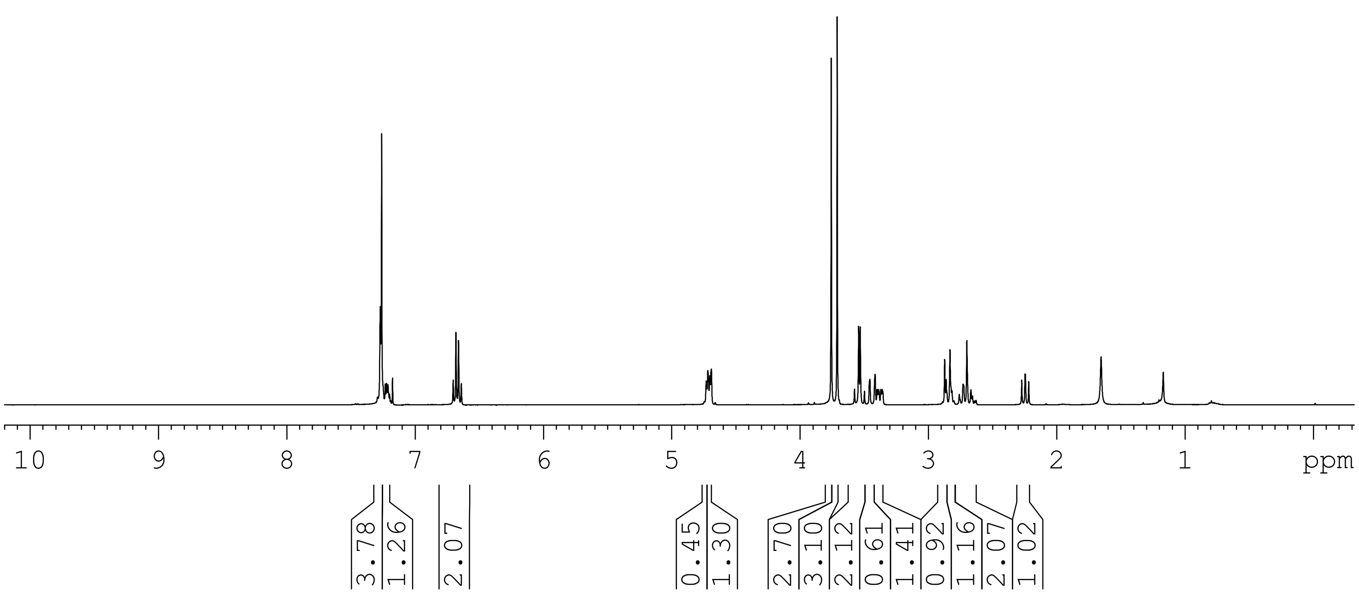

Current Data Parameters
NAME RS-I-3,5-BN-PZQ-CY
EXPNO 1
PROCNO 1

F2 - Acquisition Parameters
Date_ 20151006
Time 22.05
INSTRUM spect
PROBHD 5 mm DUL 13C-1
PULPROG zg30
TD 65536
SOLVENT CDCl3
NS 16
DS 2
SWH 8223.685 Hz
FIDRES 0.125483 Hz
AQ 3.9846387 sec
RG 161
DW 60.800 usec
DE 6.00 usec
TE 296.4 K
D1 1.00000000 sec
TD0 1

======== CHANNEL f1 ========
NUC1 1H
P1 11.42 usec
PL1 -3.00 dB
SFO1 400.1324710 MHz

F2 - Processing parameters
SI 32768
SF 400.1300382 MHz
WDW EM
SSB 0
LB 0.30 Hz
GB 0
PC 1.00

C13CPD CDCl3 {D:\CRR} KOPAL 1

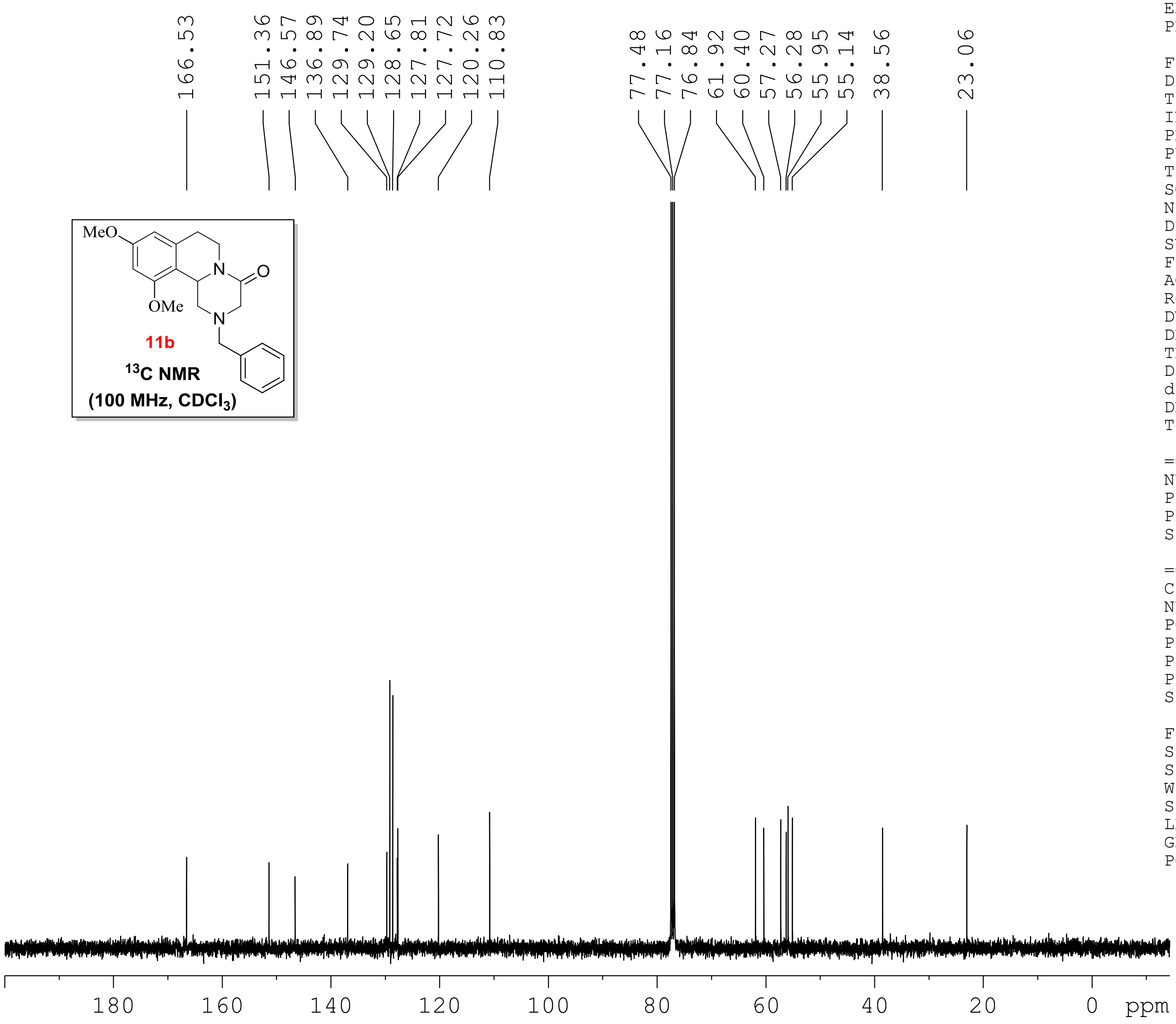

Current Data Parameters
NAME RS-I-3,5-BN-PZQ-CY
EXPNO 2
PROCNO 1

F2 - Acquisition Parameters
Date_ 20151006
Time 22.15
INSTRUM spect
PROBHD 5 mm DUL 13C-1
PULPROG zgpg30
TD 65536
SOLVENT CDCl3
NS 198
DS 4
SWH 24038.461 Hz
FIDRES 0.366798 Hz
AQ 1.3631988 sec
RG 71.8
DW 20.800 usec
DE 6.00 usec
TE 297.0 K
D1 2.00000000 sec
d11 0.03000000 sec
DELTA 1.89999998 sec
TD0 1

======== CHANNEL f1 ========
NUC1 13C
P1 9.15 usec
PL1 0.00 dB
SFO1 100.6228298 MHz

======== CHANNEL f2 ========
CPDPRG2 waltz16
NUC2 1H
PCPD2 90.00 usec
PL12 14.90 dB
PL13 14.90 dB
PL2 -3.00 dB
SFO2 400.1316005 MHz

F2 - Processing parameters
SI 32768
SF 100.6127558 MHz
WDW EM
SSB 0
LB 1.00 Hz
GB 0
PC 1.40

PROTON CDCl3 {D:\CRR} KOPAL 1

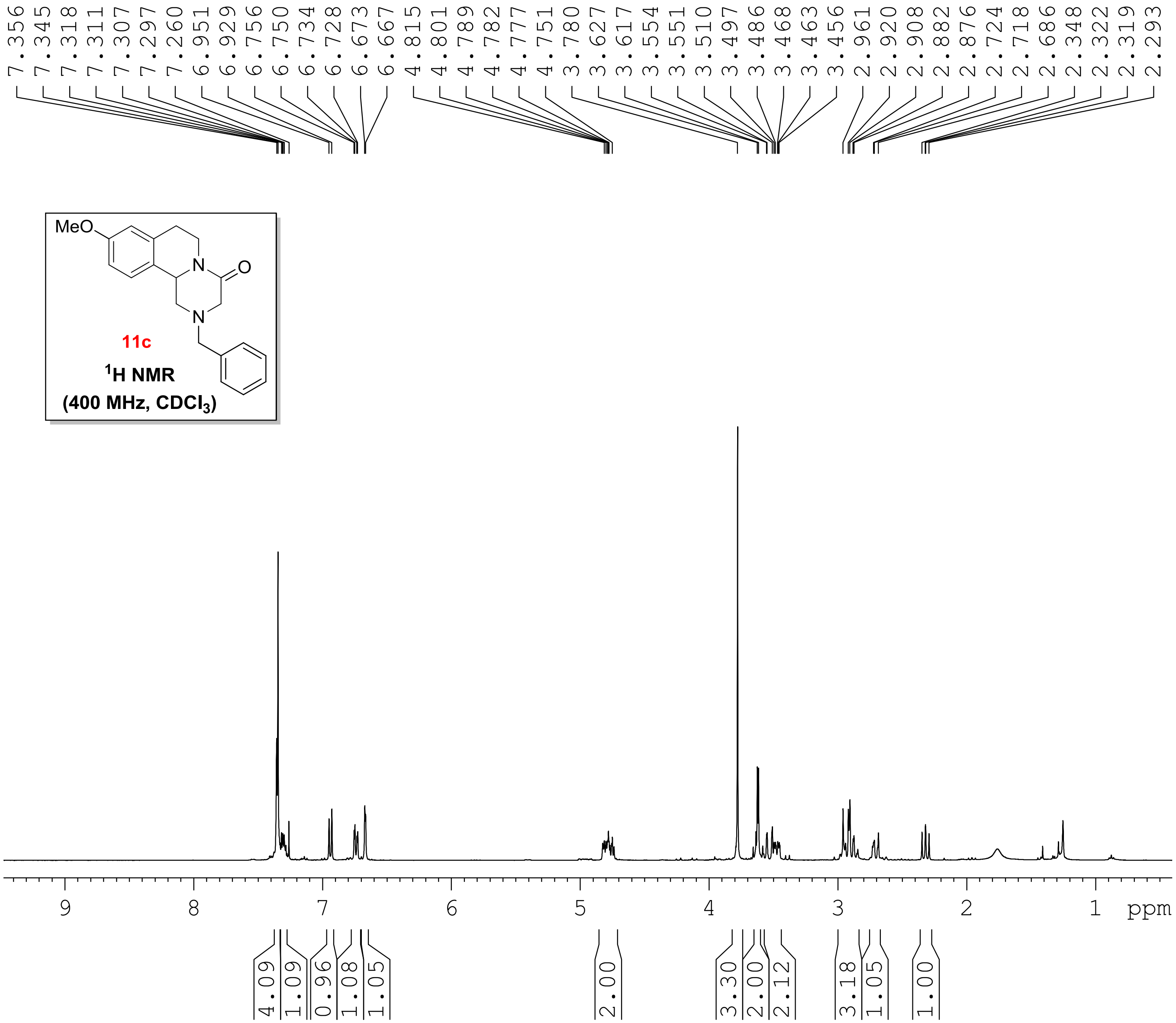

Current Data Parameters
NAME RS-I-3-BN-PZQ-CY
EXPNO 3
PROCNO 1

F2 - Acquisition Parameters
Date_ 20150927
Time 15.18
INSTRUM spect
PROBHD 5 mm DUL 13C-1
PULPROG zg30
TD 65536
SOLVENT CDCl3
NS 16
DS 2
SWH 8223.685 Hz
FIDRES 0.125483 Hz
AQ 3.9846387 sec
RG 181
DW 60.800 usec
DE 6.00 usec
TE 294.5 K
D1 1.00000000 sec
TD0 1

======== CHANNEL f1 ========
NUC1 1H
P1 11.42 usec
PL1 -3.00 dB
SFO1 400.1324710 MHz

F2 - Processing parameters
SI 32768
SF 400.1300050 MHz
WDW EM
SSB 0
LB 0.30 Hz
GB 0
PC 1.00

C13CPD CDCl3 {D:\CRR} KOPAL 1

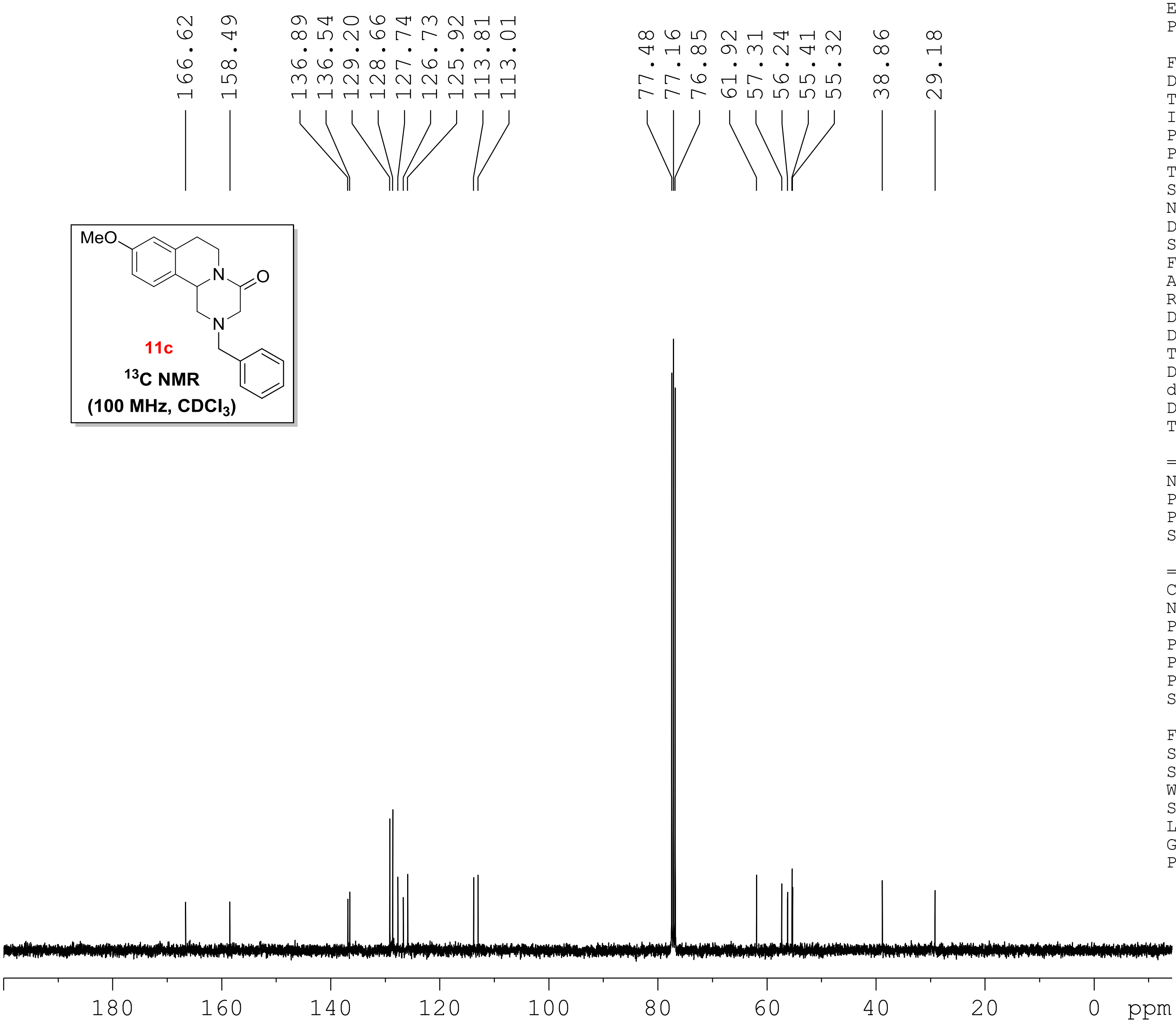


```
Current Data Parameters
NAME        RS-I-3-BN-PZQ-CY
EXPNO                      4
PROCNO                     1

F2 - Acquisition Parameters
Date_               20150927
Time                   15.26
INSTRUM                spect
PROBHD     5 mm DUL 13C-1
PULPROG              zgpg30
TD                     65536
SOLVENT                CDCl3
NS                       137
DS                         4
SWH               24038.461 Hz
FIDRES             0.366798 Hz
AQ                1.3631988 sec
RG                        64
DW                    20.800 usec
DE                      6.00 usec
TE                     295.2 K
D1               2.00000000 sec
d11              0.03000000 sec
DELTA            1.89999998 sec
TD0                        1

======== CHANNEL f1 ========
NUC1                     13C
P1                      9.15 usec
PL1                     0.00 dB
SFO1             100.6228298 MHz

======== CHANNEL f2 ========
CPDPRG2              waltz16
NUC2                      1H
PCPD2                  90.00 usec
PL12                   14.90 dB
PL13                   14.90 dB
PL2                    -3.00 dB
SFO2             400.1316005 MHz

F2 - Processing parameters
SI                     32768
SF               100.6127551 MHz
WDW                       EM
SSB                        0
LB                      1.00 Hz
GB                         0
PC                      1.40
```

PROTON CDCl3 {D:\CRR} KOPAL 1

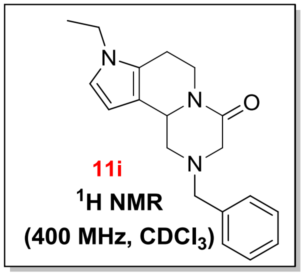

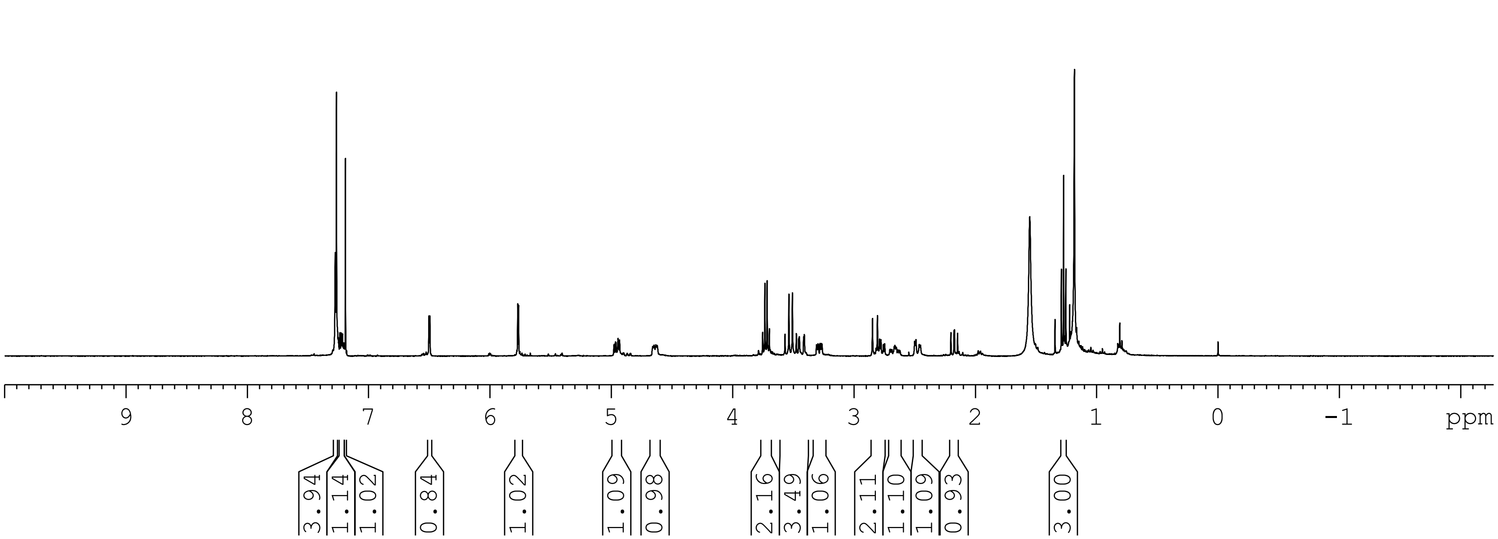

Current Data Parameters
NAME RS-I-PY-CY
EXPNO 2
PROCNO 1

F2 - Acquisition Parameters
Date_ 20151002
Time 17.59
INSTRUM spect
PROBHD 5 mm DUL 13C-1
PULPROG zg30
TD 65536
SOLVENT CDCl3
NS 16
DS 2
SWH 8223.685 Hz
FIDRES 0.125483 Hz
AQ 3.9846387 sec
RG 256
DW 60.800 usec
DE 6.00 usec
TE 294.6 K
D1 1.00000000 sec
TD0 1

======== CHANNEL f1 ========
NUC1 1H
P1 11.42 usec
PL1 -3.00 dB
SFO1 400.1324710 MHz

F2 - Processing parameters
SI 32768
SF 400.1300323 MHz
WDW EM
SSB 0
LB 0.30 Hz
GB 0
PC 1.00

C13CPD CDCl3 {D:\CRR} KOPAL 1

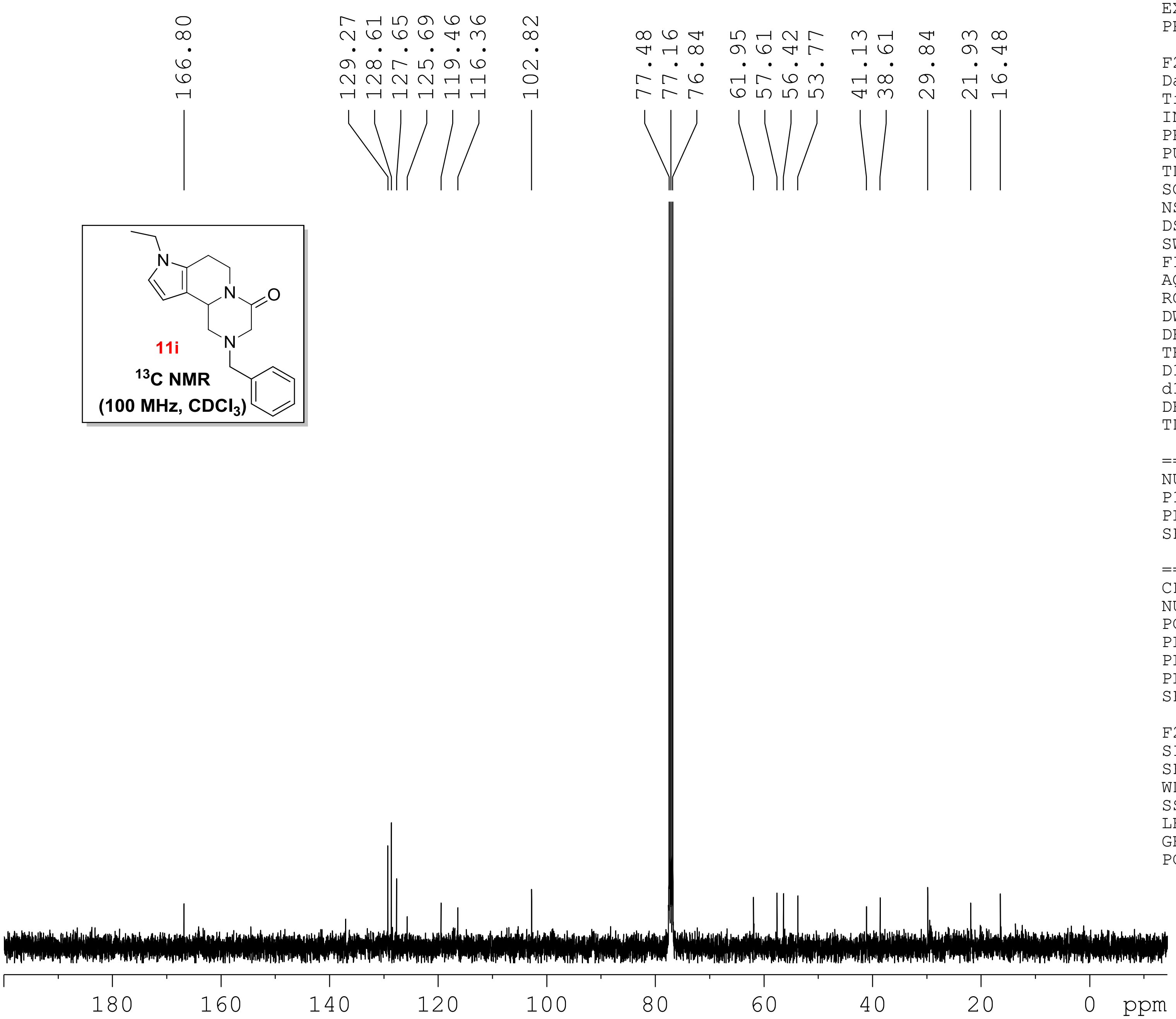


```
Current Data Parameters
NAME          RS-I-PY-CY
EXPNO                  3
PROCNO                 1

F2 - Acquisition Parameters
Date_           20151002
Time               18.29
INSTRUM            spect
PROBHD    5 mm DUL 13C-1
PULPROG          zgpg30
TD                65536
SOLVENT           CDCl3
NS                  512
DS                    4
SWH           24038.461 Hz
FIDRES         0.366798 Hz
AQ            1.3631988 sec
RG                   57
DW               20.800 usec
DE                 6.00 usec
TE                295.1 K
D1           2.00000000 sec
d11          0.03000000 sec
DELTA        1.89999998 sec
TD0                   1

======== CHANNEL f1 ========
NUC1                13C
P1                 9.15 usec
PL1                0.00 dB
SFO1        100.6228298 MHz

======== CHANNEL f2 ========
CPDPRG2         waltz16
NUC2                 1H
PCPD2             90.00 usec
PL12              14.90 dB
PL13              14.90 dB
PL2               -3.00 dB
SFO2        400.1316005 MHz

F2 - Processing parameters
SI                32768
SF          100.6127539 MHz
WDW                  EM
SSB                   0
LB                 1.00 Hz
GB                    0
PC                 1.40
```

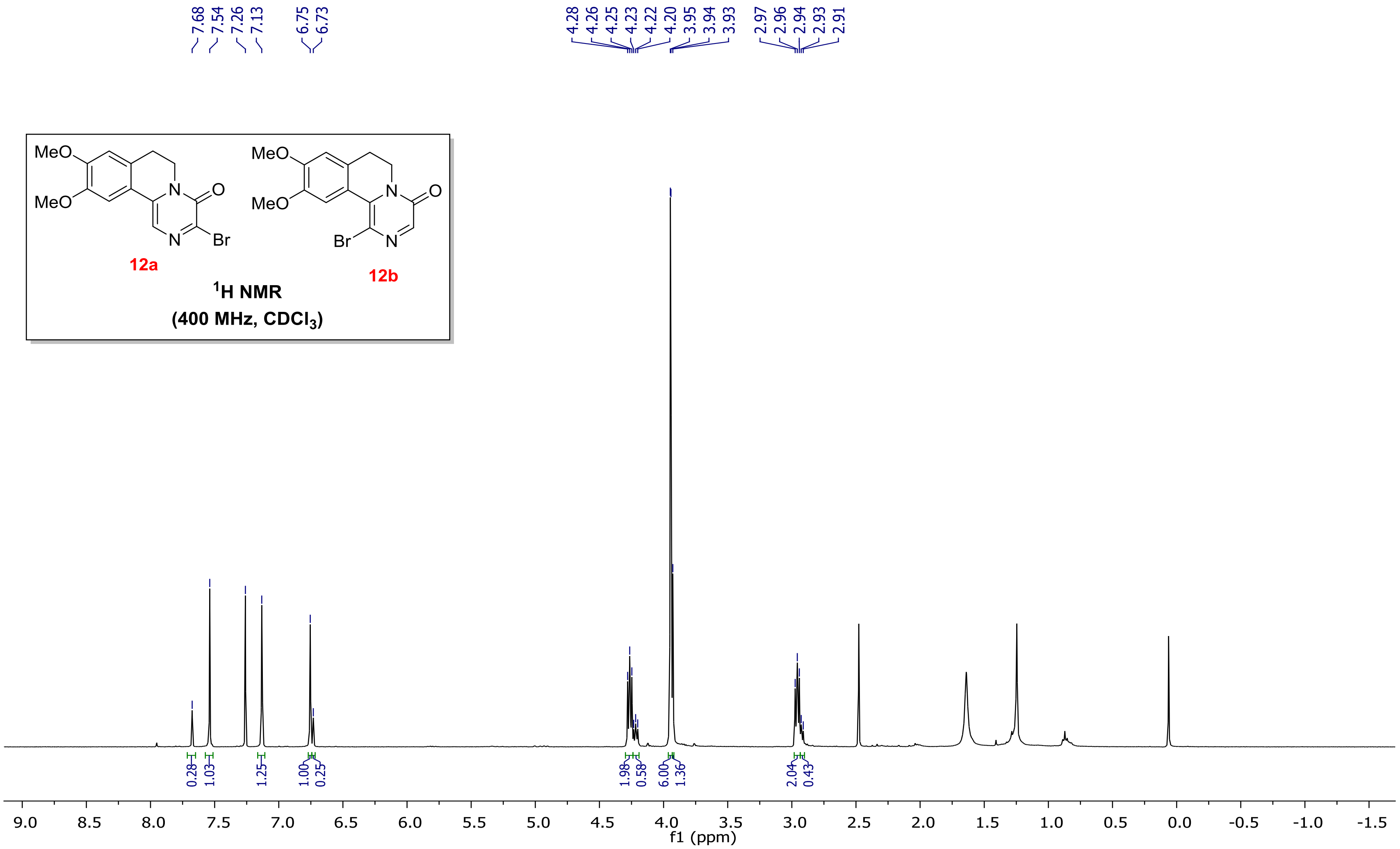
7.68
7.54
7.26
7.13
6.75
6.73
4.28
4.26
4.25
4.23
4.22
4.20
3.95
3.94
3.93
2.97
2.96
2.94
2.93
2.91
MeO
MeO
N
O
N
Br
12a
MeO
MeO
N
O
Br
N
12b
1H NMR
(400 MHz, CDCl3)
0.28
1.03
1.25
1.00
0.25
1.98
0.58
6.00
1.36
2.04
0.43
9.0
8.5
8.0
7.5
7.0
6.5
6.0
5.5
5.0
4.5
4.0
3.5
3.0
2.5
2.0
1.5
1.0
0.5
0.0
-0.5
-1.0
-1.5
f1 (ppm)

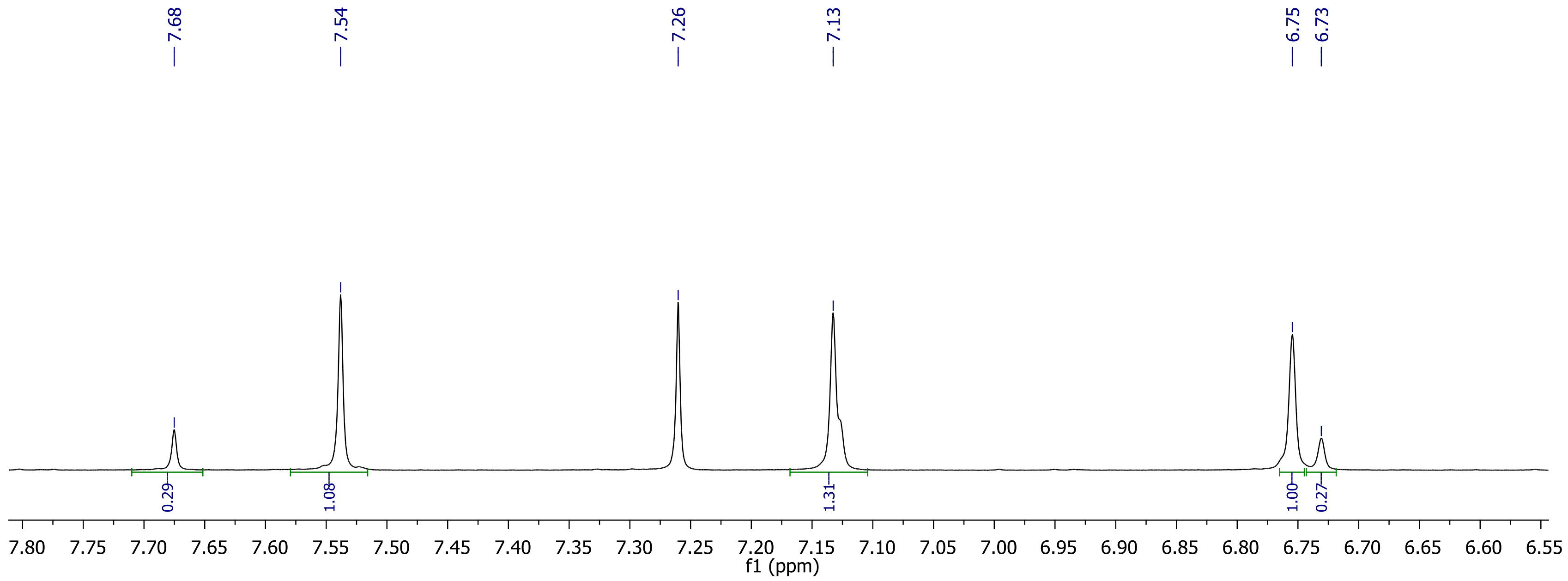
7.68
7.54
7.26
7.13
6.75
6.73
0.29
1.08
1.31
1.00
0.27
7.80 7.75 7.70 7.65 7.60 7.55 7.50 7.45 7.40 7.35 7.30 7.25 7.20 7.15 7.10 7.05 7.00 6.95 6.90 6.85 6.80 6.75 6.70 6.65 6.60 6.55
f1 (ppm)

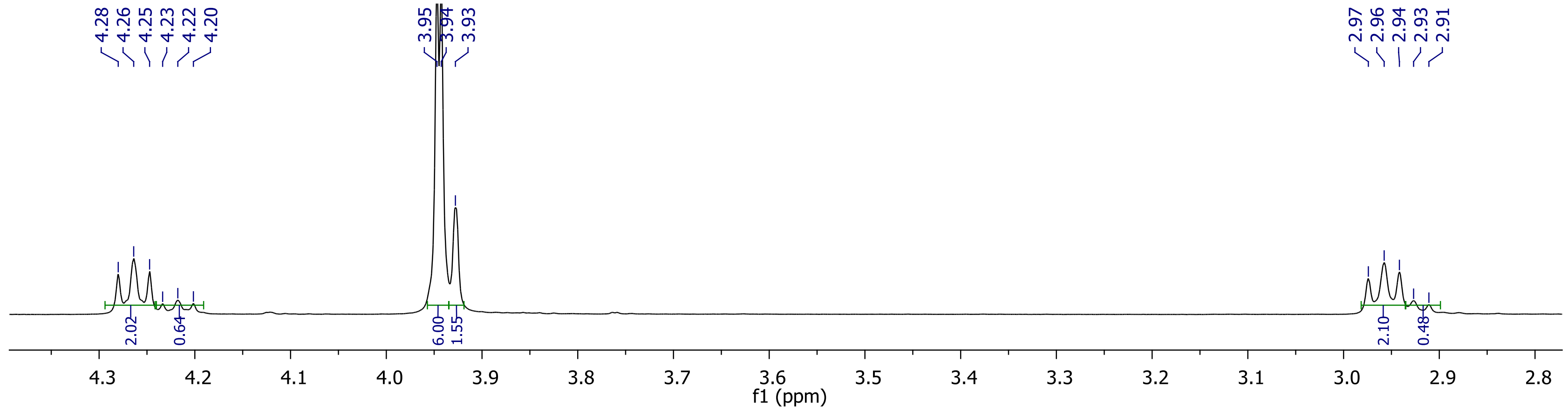
4.28
4.26
4.25
4.23
4.22
4.20
3.95
3.94
3.93
2.97
2.96
2.94
2.93
2.91
2.02
0.64
6.00
1.55
2.10
0.48
4.3 4.2 4.1 4.0 3.9 3.8 3.7 3.6 3.5 3.4 3.3 3.2 3.1 3.0 2.9 2.8
f1 (ppm)

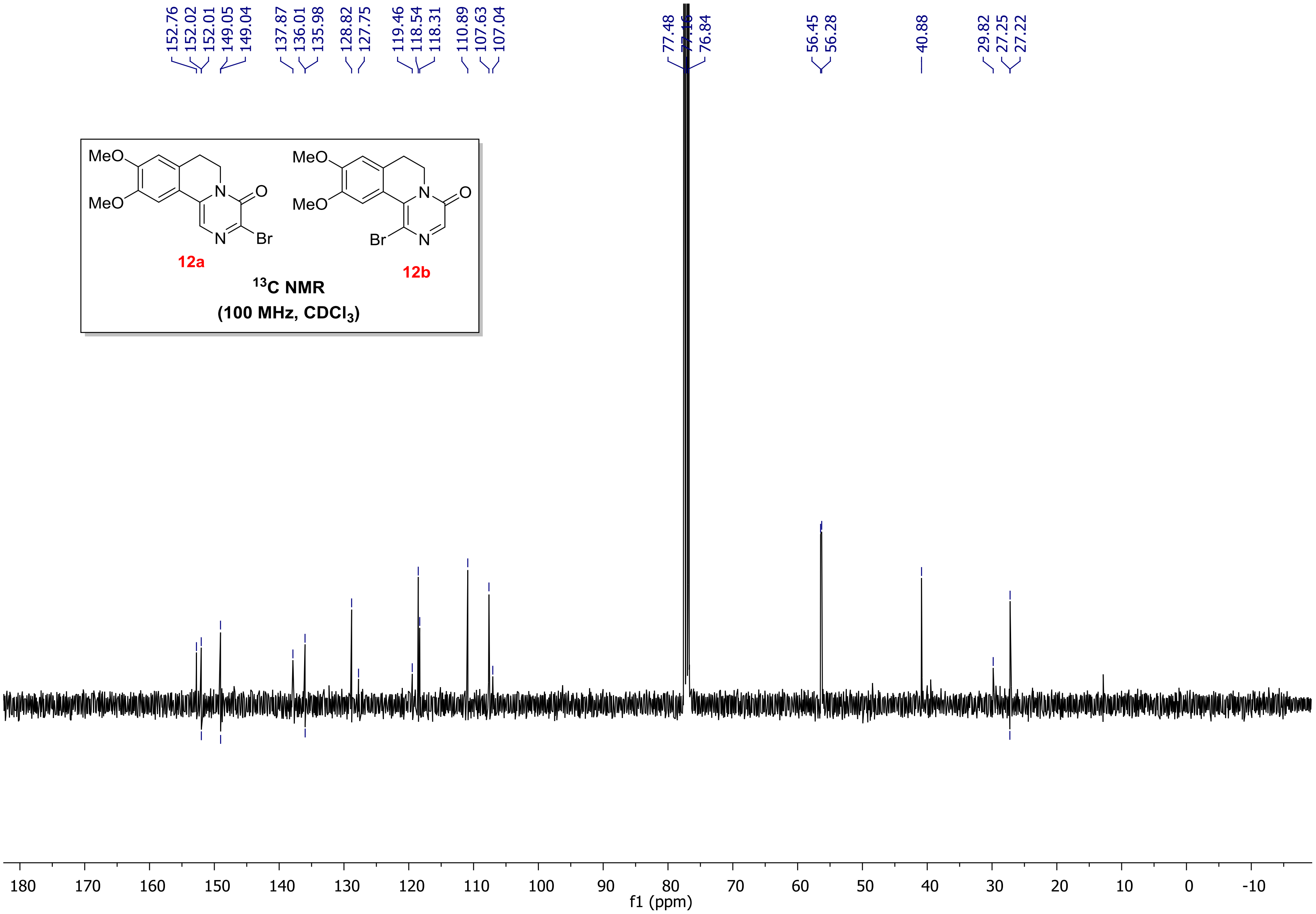
152.76
152.02
152.01
149.05
149.04
137.87
136.01
135.98
128.82
127.75
119.46
118.54
118.31
110.89
107.63
107.04
77.48
77.16
76.84
56.45
56.28
40.88
29.82
27.25
27.22
MeO
MeO
N
O
N
Br
12a
12b
^{13}C NMR
(100 MHz, $CDCl_3$)
f1 (ppm)

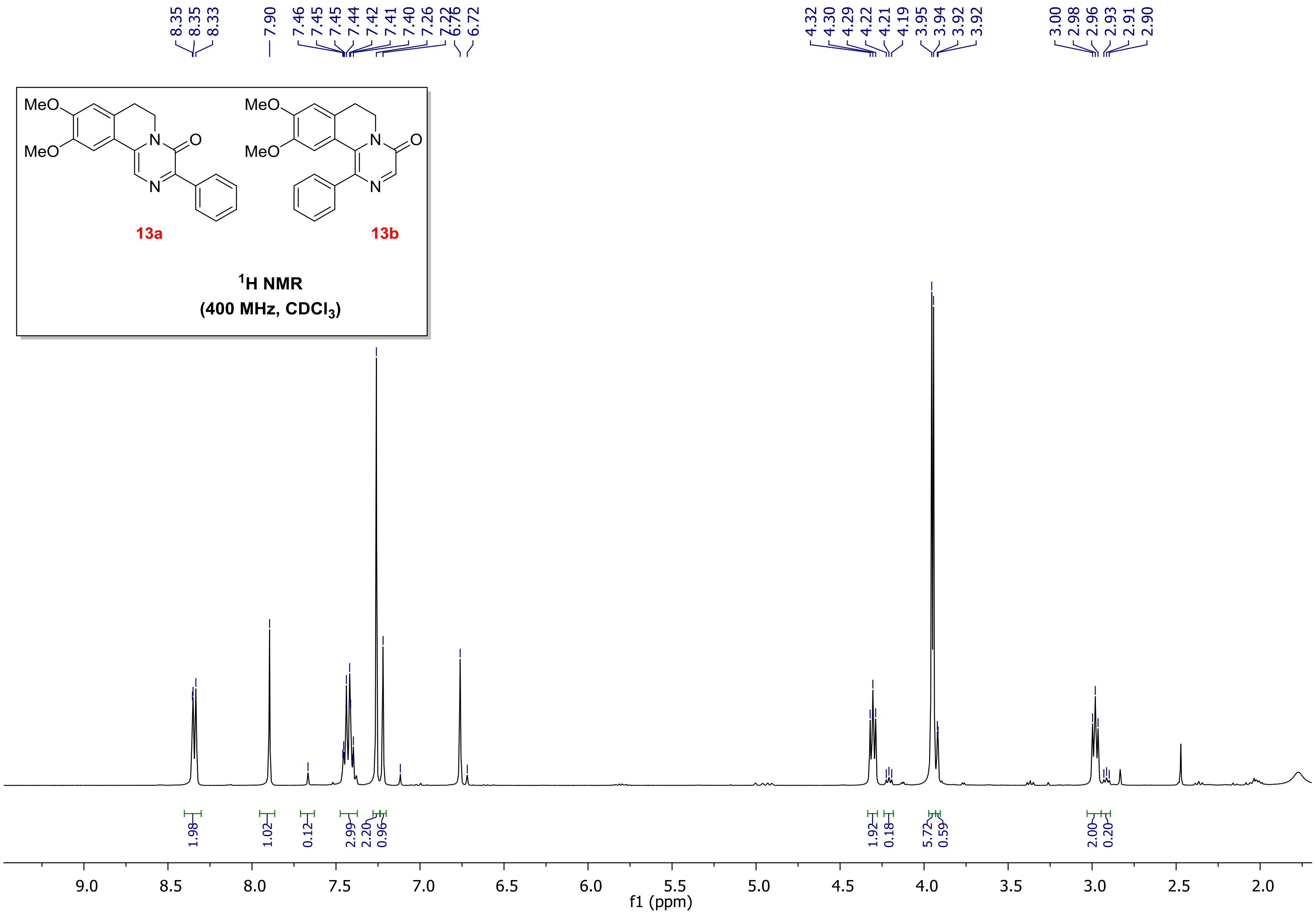
8.35 8.35 8.33
7.90
7.46 7.45 7.45 7.44 7.42 7.41 7.40 7.26 7.22 6.76 6.72
4.32 4.30 4.29 4.22 4.21 4.19 3.95 3.94 3.92 3.92
3.00 2.98 2.96 2.93 2.91 2.90
MeO
MeO
N
O
N
13a
13b
1H NMR
(400 MHz, CDCl3)
1.98
1.02
0.12
2.99
2.20
0.96
1.92
0.18
5.72
0.59
2.00
0.20
9.0 8.5 8.0 7.5 7.0 6.5 6.0 5.5 5.0 4.5 4.0 3.5 3.0 2.5 2.0
f1 (ppm)

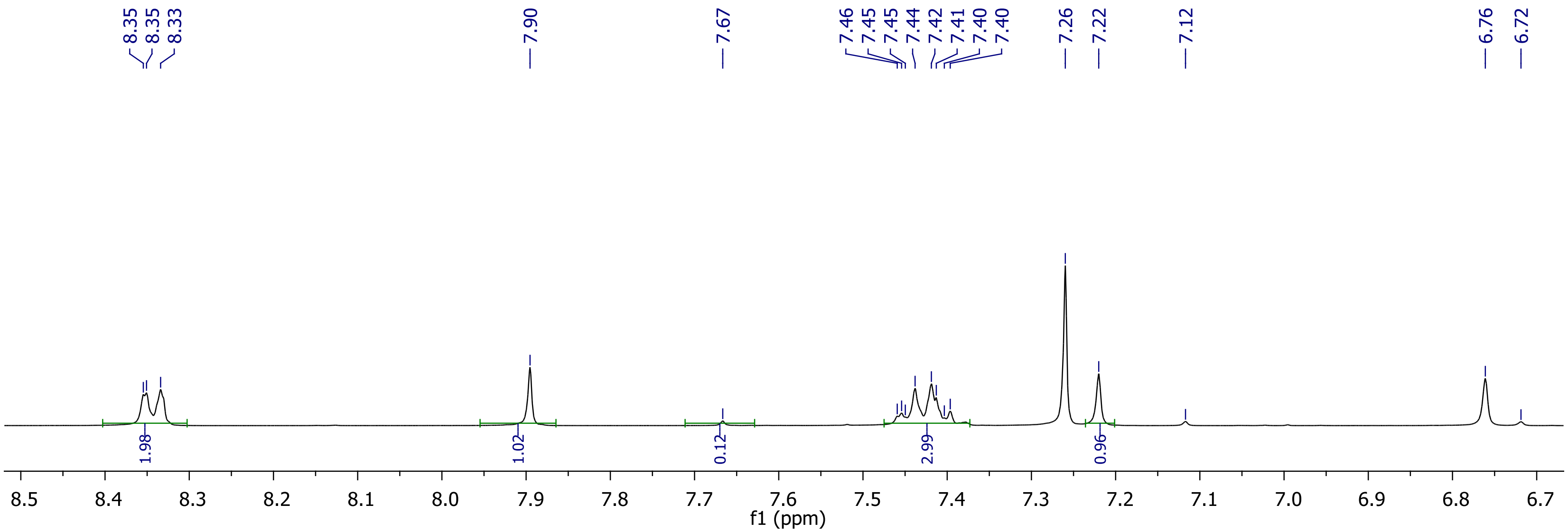
8.35
8.35
8.33
7.90
7.67
7.46
7.45
7.45
7.44
7.42
7.41
7.40
7.40
7.26
7.22
7.12
6.76
6.72
1.98
1.02
0.12
2.99
0.96
f1 (ppm)

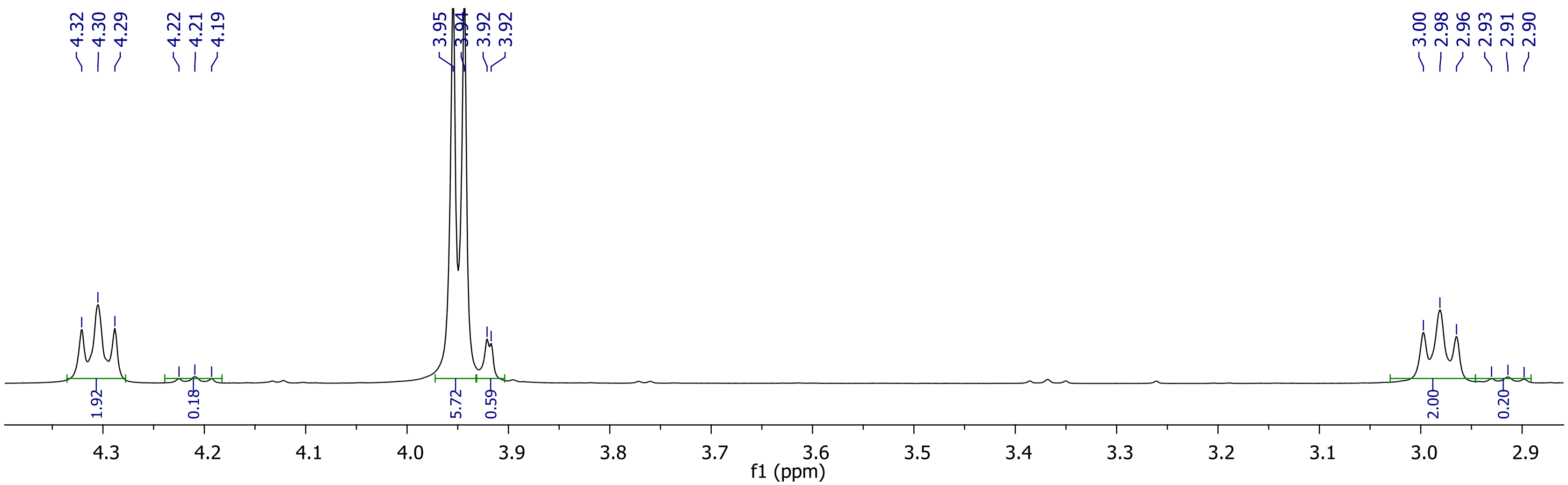
4.32
4.30
4.29
4.22
4.21
4.19
3.95
3.94
3.92
3.92
3.00
2.98
2.96
2.93
2.91
2.90
1.92
0.18
5.72
0.59
2.00
0.20
f1 (ppm)

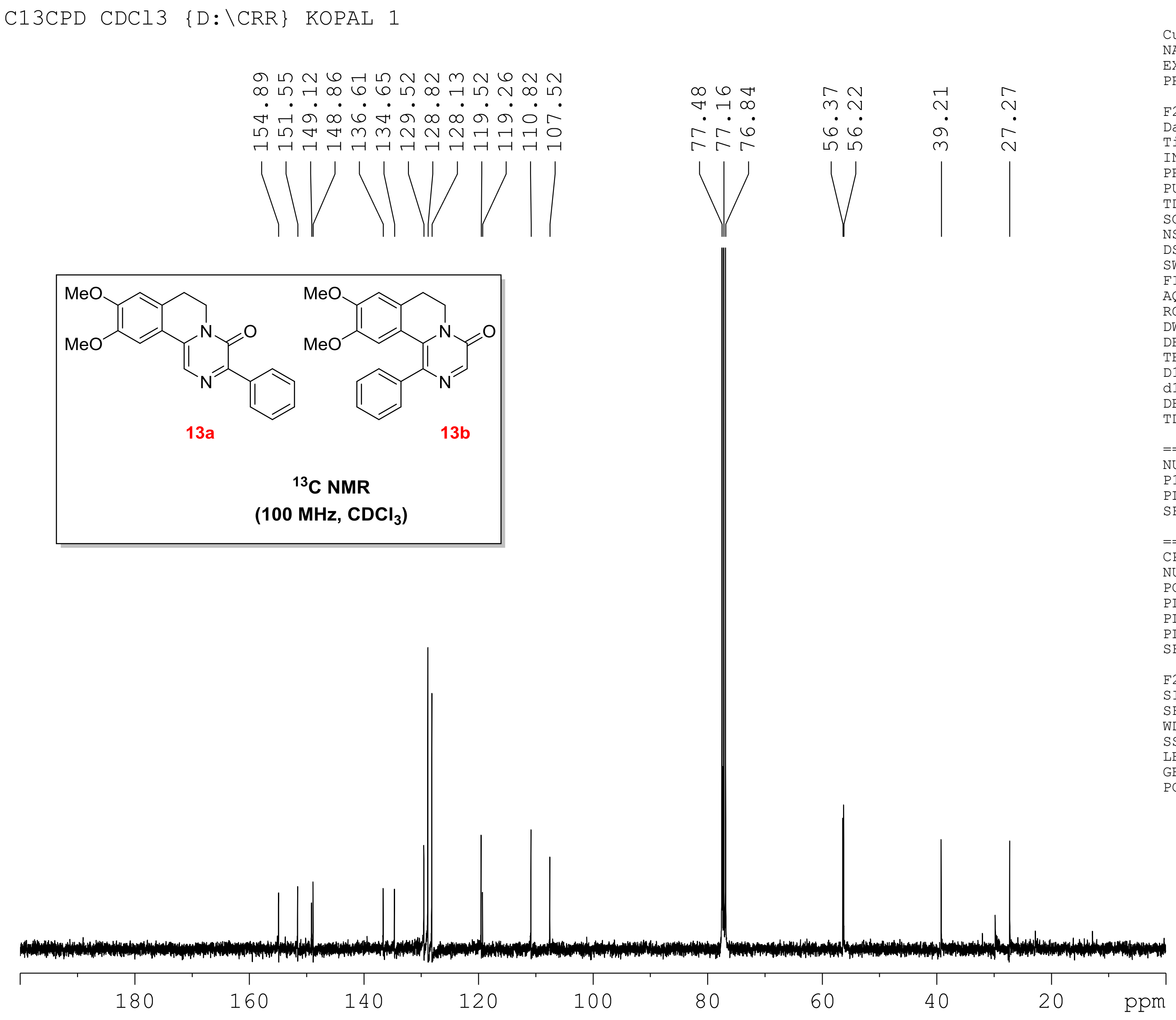


```
Current Data Parameters
NAME     RS-I-3,4-PZQ-CY-2-COUP
EXPNO                     2
PROCNO                    1

F2 - Acquisition Parameters
Date_              20160227
Time                  20.08
INSTRUM               spect
PROBHD   5 mm DUL 13C-1
PULPROG             zgpg30
TD                   65536
SOLVENT              CDCl3
NS                     264
DS                       4
SWH             24038.461 Hz
FIDRES           0.366798 Hz
AQ              1.3631988 sec
RG                    28.5
DW                  20.800 usec
DE                    6.00 usec
TE                   295.5 K
D1             2.00000000 sec
d11            0.03000000 sec
DELTA          1.89999998 sec
TD0                      1

======== CHANNEL f1 ========
NUC1                   13C
P1                    9.15 usec
PL1                   0.00 dB
SFO1           100.6228298 MHz

======== CHANNEL f2 ========
CPDPRG2            waltz16
NUC2                    1H
PCPD2                90.00 usec
PL12                 14.90 dB
PL13                 14.90 dB
PL2                  -3.00 dB
SFO2           400.1316005 MHz

F2 - Processing parameters
SI                   32768
SF             100.6127569 MHz
WDW                     EM
SSB                      0
LB                    1.00 Hz
GB                       0
PC                    1.40
```

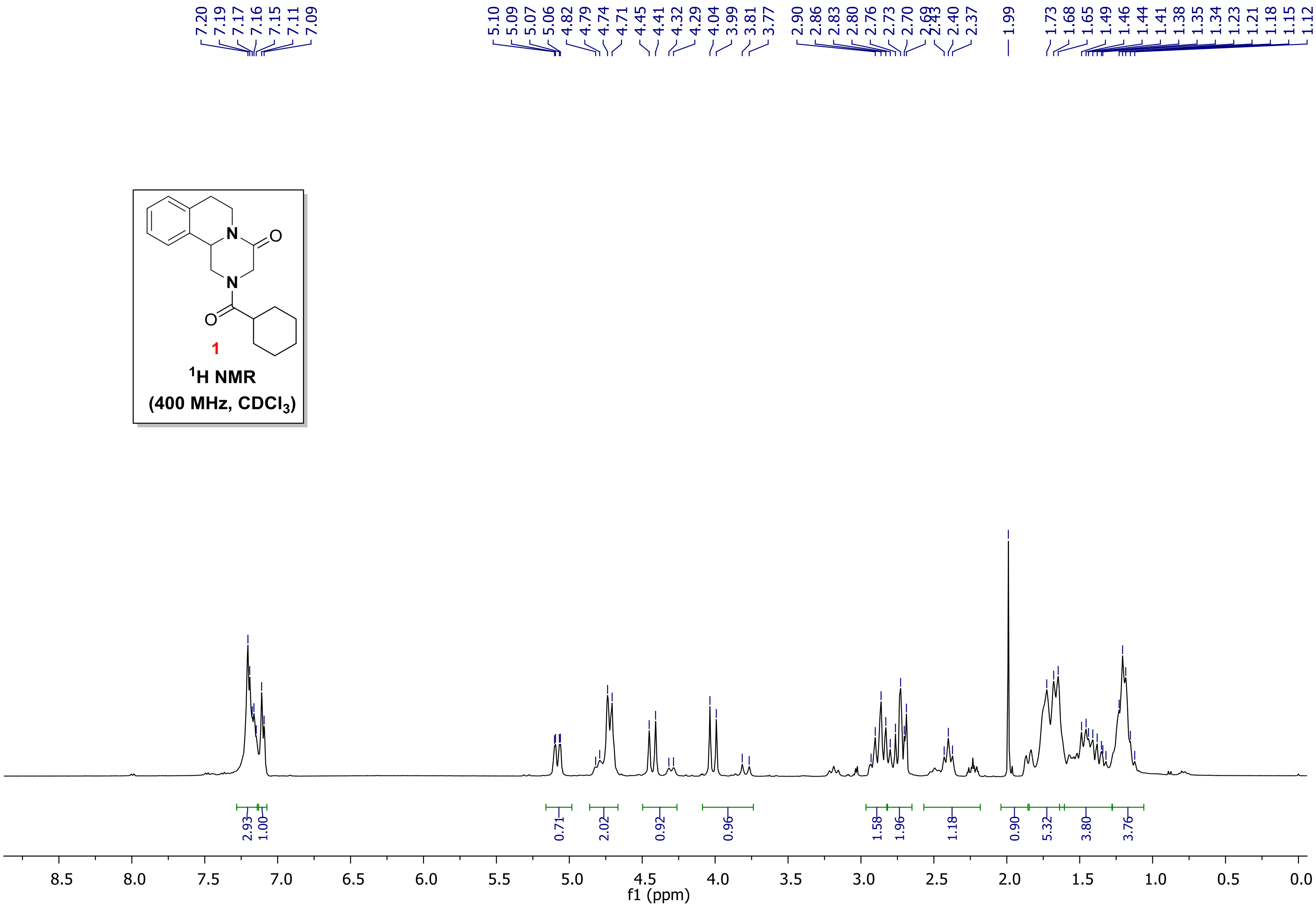
1
1H NMR
(400 MHz, CDCl3)
7.20 7.19 7.17 7.16 7.15 7.11 7.09
5.10 5.09 5.07 5.06 4.82 4.79 4.74 4.71 4.45 4.41 4.32 4.29 4.04 3.99 3.81 3.77
2.90 2.86 2.83 2.80 2.76 2.73 2.70 2.69 2.43 2.40 2.37
1.99
1.73 1.68 1.65 1.49 1.46 1.44 1.41 1.38 1.35 1.34 1.23 1.21 1.18 1.15 1.12
2.93 1.00 0.71 2.02 0.92 0.96 1.58 1.96 1.18 0.90 5.32 3.80 3.76
f1 (ppm)

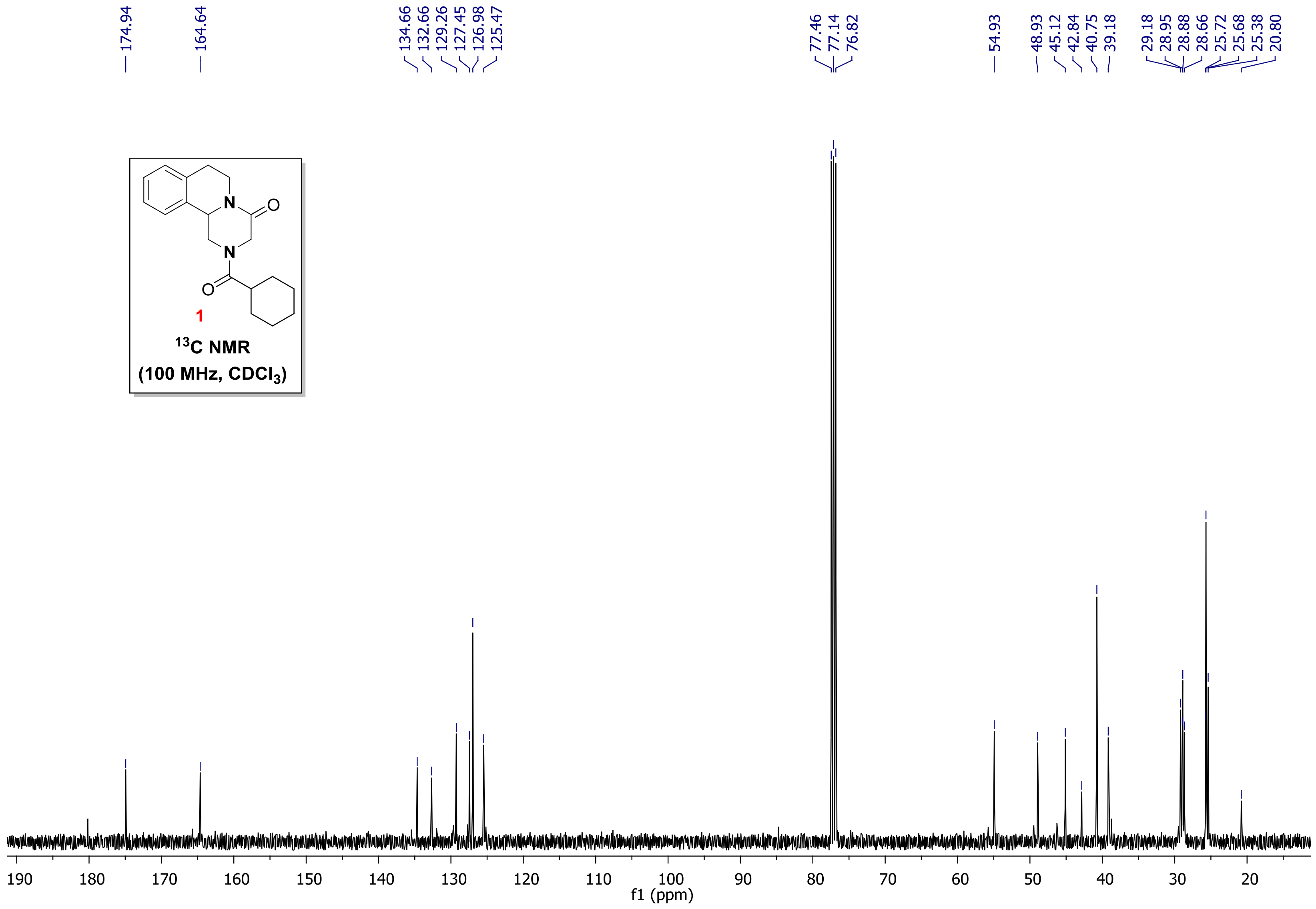
174.94
164.64
134.66
132.66
129.26
127.45
126.98
125.47
77.46
77.14
76.82
54.93
48.93
45.12
42.84
40.75
39.18
29.18
28.95
28.88
28.66
25.72
25.68
25.38
20.80
N
O
N
O
1
13C NMR
(100 MHz, CDCl3)
190
180
170
160
150
140
130
120
110
100
90
80
70
60
50
40
30
20
f1 (ppm)
